# Genetics of lineage diversification and the evolution of host usage in the economically important wheat curl mite, *Aceria tosichella* Keifer, 1969

**DOI:** 10.1186/s12862-018-1234-x

**Published:** 2018-08-07

**Authors:** Anna Skoracka, Luís Filipe Lopes, Maria Judite Alves, Adam Miller, Mariusz Lewandowski, Wiktoria Szydło, Agnieszka Majer, Elżbieta Różańska, Lechosław Kuczyński

**Affiliations:** 1 Population Ecology Lab, Faculty of Biology, Adam Mickiewicz University, Poznań, Umultowska 89, 61–614 Poznań, Poland; 2 Museu Nacional de História Natural e da Ciência & Centre for Ecology, Evolution and Environmental Changes (cE3c), University of Lisbon, Rua da Escola Politécnica 58, 1250-102 Lisbon, Portugal; 3 Deakin University, Geelong, Australia; 4 School of Life and Environmental Sciences, Centre for Integrative Ecology, Warrnambool, Vic 3280 Australia; 5 Department of Applied Entomology, Faculty of Horticulture, Biotechnology and Landscape Architecture, Warsaw University of Life Sciences – SGGW, Nowoursynowska 159, 02-776 Warsaw, Poland; 6 Department of Entomology, University of Nebraska-Lincoln, 103 Entomology Hall, Lincoln, NE 68583-0816 USA; 7 Department of Botany, Faculty of Agriculture and Biology, Warsaw University of Life Sciences – SGGW, Nowoursynowska 159, 02-776 Warsaw, Poland

**Keywords:** *Aceria tosichella*, Demographic history, Genetic diversity, Host-associations, Lineage diversification, Species delimitation

## Abstract

**Background:**

Understanding the mechanisms that underlie the diversification of herbivores through interactions with their hosts is important for their diversity assessment and identification of expansion events, particularly in a human-altered world where evolutionary processes can be exacerbated. We studied patterns of host usage and genetic structure in the wheat curl mite complex (WCM), *Aceria tosichella*, a major pest of the world’s grain industry, to identify the factors behind its extensive diversification.

**Results:**

We expanded on previous phylogenetic research, demonstrating deep lineage diversification within the taxon, a complex of distinctive host specialist and generalist lineages more diverse than previously assumed. Time-calibrated phylogenetic reconstruction inferred from mitochondrial DNA sequence data suggests that lineage diversification pre-dates the influence of agricultural practices, and lineages started to radiate in the mid Miocene when major radiations of C4 grasses is known to have occurred. Furthermore, we demonstrated that host specificity is not phylogenetically constrained, while host generalization appears to be a more derived trait coinciding with the expansion of the world’s grasslands. Demographic history of specialist lineages have been more stable when compared to generalists, and their expansion pre-dated all generalist lineages. The lack of host-associated genetic structure of generalists indicates gene flow between mite populations from different hosts.

**Conclusions:**

Our analyses demonstrated that WCM is an unexpectedly diverse complex of genetic lineages and its differentiation is likely associated with the time of diversification and expansion of its hosts. Signatures of demographic histories and expansion of generalists are consistent with the observed proliferation of the globally most common lineages. The apparent lack of constrains on host use, coupled with a high colonization potential, hinders mite management, which may be further compromised by host range expansion. This study provides a significant contribution to the growing literature on host-association and diversification in herbivorous invertebrates.

**Electronic supplementary material:**

The online version of this article (10.1186/s12862-018-1234-x) contains supplementary material, which is available to authorized users.

## Background

Niche breadth varies significantly among species, with life history traits, physical tolerances, and evolutionary adaptations directly influencing the range of habitats which species can successfully colonize and thrive within [1, 2]. Consequently, generalist and specialist species differ in traits and adaptations that facilitate the inhabitation of wide and narrow environmental ranges, respectively [3–5]. Species that have obligatory associations with animal or plant hosts, which function as their local habitat, provide ideal model systems for studying niche breadth and host-associated genetic differentiation [6–9]. Long-term host affiliation in the absence of gene flow across populations occupying different hosts can lead to their genetic divergence due to host specialization [6–8, 10]. Yet, these processes are not static, and host ranges can change dynamically through time with environmental changes, specialists giving rise to generalists and vice versa. This nature of host range expansion and contraction is thought to be largely responsible for the astounding diversity of parasite and herbivorous invertebrate taxa that exists today [10–12].

Host-associations can change both markedly and rapidly under different scenarios [11]. Populations of generalist species might restrict their host range as a result of diminishing host availability or costs of host adaptation [5]. Conversely, specialist species might expand their host range or transit from one exclusive host to another (host shift) as a consequence of environmental change, including anthropogenic influences [13, 14]. Numerous studies of host-associated differentiation have contributed greatly to our understanding of host shifts and host range evolution [15–20], with the sympatric host race formation in the apple maggot fly *Rhagoletis pomonella* being the most well-known example [21]. Yet there is still much to learn about mechanisms behind the diversification of herbivores and parasites through interactions with their hosts [22, 23].

The host specificity is among the most important life-history traits that influences population size and genetic structure [9, 24]. Studies on parasitic lice in birds [25] and nematodes in livestock [26] indicate that the degree of genetic structuring in host specialists will typically be higher and their genetic diversity will be reduced. With an exception of cases where hosts are highly prolific, gene flow in specialists is often more restricted due to reduced dispersal capacity, population size, or host availability [24]. Yet these patterns have been established with grounds on observations based on laboratory models thus far, and therefore it is still unclear how broadly this concept applies to natural systems.

Understanding patterns of genetic diversity and host ranges is particularly important for identifying risks associated with parasites and herbivorous pests, especially those with high invasive potential [27, 28]. The wheat curl mite *Aceria tosichella* Keifer (WCM hereafter) is an obligate plant-feeding pest in wheat and many other cereal crops, with a high colonization potential driven by an ability for long distance dispersal and reproduction by arhenotokous parthenogenesis [29]. Mite infestations can lead to yield losses through direct feeding and the transmission of several plant viruses [30, 31]. *Wheat streak mosaic virus* (WSMV) is a particularly damaging viral pathogen that primarily affects wheat in cropping regions of the Americas, Northern Africa, Asia, Europe and Australia, and can be responsible for annual yield losses of approximately 5%, with localized areas suffering complete yield loss [32–34]. Recent studies proved that the WCM is a complex of distinct mitochondrial lineages that differ in their host preference [35–38], with two globally distributed lineages (viz. MT-1 and MT-8) that appear to be responsible for global WSMV transmission [39, 40]. Recent sampling efforts suggest that lineage diversity within the WCM complex is currently understated, and further work is needed to characterize the true extent of genetic diversity and host ranges of the lineages [41, 42].

Of the WCM lineages described to date, all appear to be morphologically indistinguishable, but differ markedly in host-acceptance traits, including host specialists and generalists [37, 38, 42]. Thus, this is an ideal system to investigate host-association dynamics and associated evolutionary processes. In this study, we tested the hypotheses: (i) whether host specificity is a phylogenetically constrained trait, and if mite lineages are restricted to hosts of certain taxonomic groups, suggesting possible co-evolution; (ii) if there is an association between the timing of lineage diversification and the historical expansion of agriculture; and (iii) if there is host-associated structuring within generalist lineages, that may be indicative of incipient speciation. We drew on all available mitochondrial Cox1 and nuclear 28S D2 sequence data, representing mites from 25 host-plant species and undertook comprehensive phylogenetic and population genetic analyses to provide insight into the extent of lineage diversification within the WCM complex, and host ranges of respective lineages. We compared patterns of genetic structure between host specialists and generalists, and identified patterns of demographic expansion or stability of host specialists and generalists.

## Methods

### Sampling of mites

The cereal hosts (bread wheat *Triticum aestivum* L., triticale, ×*Triticosecale* Wittm. ex *A. Camus*, rye *Secale cereale* L., oat *Avena sativa* L., barley *Hordeum vulgare* L., maize *Zea mays* L.) and wild grass hosts (cockspur *Echinochloa crus-galli* (L.) Beauv, quackgrass *Elymus repens* (L.) Gould, tall oat-grass *Arrhenatherum elatius* (L.) P. Beauv. ex J. & C. Presl, soft brome *Bromus hordeaceus* L., smooth brome *Bromus inermis* Leyss., timothy-grass *Phleum pretense* L., and wall barley *Hordeum murinum* L.) of the wheat curl mite were collected in the field from a total of 316 sample sites in Poland (covering an area of 311,888 km^2^) between June and August 2012–2014 (see Additional file 1: Table S1). Plants were examined in the laboratory for the presence of WCM specimens under a stereo-microscope. Individual specimens were collected using insect pin and preserved in Eppendorf tubes with 180 μl of ATL extraction-buffer (Qiagen GmbH, Hilden, Germany) for subsequent DNA extraction and genetic analysis. Altogether 1187 mite specimens were collected (from 1 to 20 specimens per one Eppendorf tube) and subsequently analyzed.

### DNA isolation, amplification and sequencing

DNA was isolated from specimens that had been stored in ATL buffer according to the non-destructive method of Dabert et al. [43]. The exoskeletons of the digested mites were preserved in 70% ethyl alcohol, and later mounted on slides according to Monfreda et al. [44] for morphological WCM identification. A fragment of the mitochondrial cytochrome c oxidase subunit I (Cox1) gene (603 bp) was amplified by PCR using the degenerate primers bcdF01 and bcdR04 [43, 45]. Amplification of the ca. 600 bp D2 region in 28S rDNA was performed using the primers D1D2fw2 [46] and 28SR0990 [47]. Reactions steps and product handling followed protocols described by Szydło et al. [48]. Products were sequenced with the same primers that were used for amplification and additionally D2 amplified fragments were sequenced with specific sequencing primers Er28SF05 and Er28SR05 [48]. Trace files were checked and edited using MEGA 6 [49].

### Mitochondrial and nuclear datasets

To be able to build an alignment consisting of sequences of comparable length (and therefore comprehensive datasets), we obtained sequences of similar length to these downloaded from Genbank. Newly generated 662 mitochondrial and 63 nuclear sequence data from WCM in Poland were combined with previously published 85 Cox1 and 46 28S rDNA D2 regions of WCM and outgroup *Trisetacus* species data [36, 39, 48, 50] for analysis (see Additional file 1: Table S1 for sample details and Genbank accession numbers). *Trisetacus* genus is a taxon belonging to the Eriophyoidae associated with coniferous plants, for which genetic differentiation according to host plants has been recorded [50]. Sequences were aligned using the MAFFT algorithm (https://www.ebi.ac.uk/Tools/msa/mafft/). Altogether 747 Cox1 (662 + 85; 603 bp) and 109 28S D2 (63 + 46; 595 bp) sequences were analyzed and evaluated. They were obtained from mite specimens from 25 plant species (13 plant species collected in Poland in the course of this study plus 12 plant species from earlier sample collections) and eight countries (Additional file 1: Table S1). Sequences from both mitochondrial and nuclear datasets were collapsed to haplotypes using FABOX 1.41 DNA collapse tool [51] resulting in a batch of 291 unique Cox1 haplotypes, 39 unique D2 sequences, and 84 unique concatenated Cox1 + D2 sequences. Alignment reliability and product authenticity of Cox1 sequences was validated by translating aligned DNA sequences into amino acids and assessing the alignments for premature stop codons. A simplified Cox1 sequence dataset with a representative set of 158 sequences (for each WCM lineage a maximum of five sequences from the same country and same host) was used for the construction of phylogenetic trees. This selection was based on preliminary trees and aimed to simplify the phylogenetic tree as well as to keep the general tree structure. Cox1 and D2 sequences obtained from the same DNA isolate were concatenated resulting in a dataset consisting of 109 concatenated haplotypes that were collapsed to 84 unique concatenated sequences. Partition congruence was analyzed using the incongruence length difference test (ILD) with 100 partition homogeneity test replicates implemented in PAUP* 4.0a147 [52].

### Phylogenetic analyses and divergence time estimation

Phylogenetic reconstructions were performed using Bayesian Inference (BI) methods implemented in BEAST 2.3.0 [53]. General Time Reversible model [54] with gamma distribution of rates across sites (GTR + G) was selected as the best fit model of evolution for each of the mtDNA and nuclear genes, based on Akaike Information Criteria (AIC) [55] implemented in JMODELTEST v.0.1.1 [56]. Operators were auto-optimized, and five independent Markov Chain Monte Carlo (MCMC) runs were performed using a Yule (speciation) tree-prior, each running for 5 × 10^6^ generations, sampling every 5000 states. Log files were examined with TRACER v.1.5 [57] to ensure that runs were sampling from the same posterior distribution, to determine appropriate burn-in, and to ensure that effective sample sizes (ESSs) of parameters of interest were greater than 1000. Tree files of independent runs were then combined with LOGCOMBINER v.2.1.3 [58], discarding the first 20% and re-sampling at lower frequency of 15,000. The maximum clade credibility (MCC) tree was recovered from a sample of 10,000 posterior trees, and branch support was annotated, using TREEANNOTATOR v.2.1.3 [58]. All analyses started with a random starting tree and seed with no root specified. Sequence data from *Trisetacus* species was used to estimate the root of the mitochondrial gene tree. Additionally, Maximum Likelihood (ML) analysis was performed in PAUP* with starting trees obtained by Neighbor-joining, and Tree bisection-reconnection (TBR) as branch swapping algorithm. Bootstrap proportions [59] were obtained to access node robustness, using 1000 bootstrap replications.

In order to test the timing of diversification between WCM mitochondrial lineages, the mitochondrial gene tree was time calibrated, with divergence times of nodes being inferred from 95% highest posterior density (HPD) intervals. Substitution rates for the Cox1 locus in mite lineages have been shown to differ by up to four orders of magnitude, and at present there is no calibrated substitution rate for Acari specifically. Consequently, we chose to work with the universal invertebrate substitution rate 0.0115 substitutions/site/million years [60, 61] with relaxed clock log normal priors with standard deviations account allowing for uncertainty (four orders of magnitude) around the mean. Substitution rates were set in BEAUti 1.7.3 [58], and TRACER was then used to obtain parameter estimates for time to the most recent common ancestor (tMRCAs) for nodes within the gene tree.

### DNA sequence-based species delineation

We implemented two DNA taxonomy approaches to evaluate the presence of additional putative mitochondrial lineages using the Cox1 dataset. (1) The general mixed Yule coalescent (GMYC) approach [62, 63] was applied to the ultrametric tree (produced by beast analyses) in R 2.15.3 [64] with the ‘splits package’. The GMYC model is a process-based approach for detecting the threshold in a gene tree at which within-species processes (i.e., coalescence) shift to between-species processes (i.e., speciation and extinction). (2) We applied the combination of the Poisson Tree Processes model for species delimitation (PTP), and a Bayesian implementation of PTP (bPTP) to infer putative lineages boundaries on a given phylogenetic input tree [65]. The PTP/bPTP model, unlike the GMYC model, requires a bifurcated phylogenetic tree, not an ultrametric tree. PTP/bPTP models speciation or branching events in terms of number of substitutions. We used the following parameters: MCMC 500,000 generations; Thinning, 100; Burnin, 0.1; Seed, 123, and assessed convergence in each case to ensure reliability of results. Additionally, the mean uncorrected p-distances with standard error estimates (obtained using a bootstrap procedure with 1000 replicates) within and between WCM lineages were calculated in MEGA6 [49].

### Diversity within WCM lineages and tests for demographic history

Genetic diversity estimates including number of haplotypes (*h*), number of polymorphic sites (*p*), haplotype diversity (*H*_*d*_) and nucleotide diversity (*л*) were calculated with ARLEQUIN v.3.5.2.2 [66] for each major clade inferred from BI analyses. We investigated the historical demography of the WCM lineages with *N* ≥ 15 by calculating Tajima’s D [67] and Fu’s Fs [68]. The significance of Fs statistic was determined with a simulated random distribution produced by 1000 permutations assuming neutrality and population equilibrium. We also analyzed the mismatch distribution of pairwise genetic differences [69]. The fit to models of population expansion was tested with the sum of squared deviations between the observed and expected mismatch (SSD) and the raggedness index (H_Rag_). The significance of H_Rag_ and SSD was determined with 1000 bootstrap replicates. Non-significant values for these statistics signify that the data do not deviate from that expected under the model of expansion. All above analyses were conducted with ARLEQUIN. Estimates of times for lineage expansion were calculated by t = τ/2 μ where τ is the number of generations and μ is the cumulative (across the sequence) probability of substitution, assuming a divergence rate of 0.0115 substitutions per nucleotide per million years and 18 generations per year.

### Tests for host associated genetic structure in multi-host WCM lineages

For WCM lineages with multi-host association, we tested for patterns of host associated genetic structure. An Analysis of Molecular Variation (AMOVA) was performed to test for significant host-associated genetic structuring, indicating ecological isolation and potential incipient speciation. Partitioning of WCM mtDNA variation according to hosts was investigated by AMOVA using pairwise F_ST_ as the distance measure with 10,000 permutations, as implemented in ARLEQUIN. We also tested for host associated structuring by comparing relatedness estimates among haplotypes inferred from Parsimony Median Joining Network analyses [70] implemented in popart 1.7. F_ST_ values between host-associated populations (with *n* ≥ 10) were computed to disclose the level of sharing genetic diversity. Additionally, nucleotide diversity (*л*) and haplotype diversity (*H*_*d*_) were calculated for each host population, within multi-host lineages, and were compared with the estimates for the whole lineage.

## Results

### Divergence between WCM lineages

#### Phylogenetic analyses and molecular dating

**Fig. 1:**
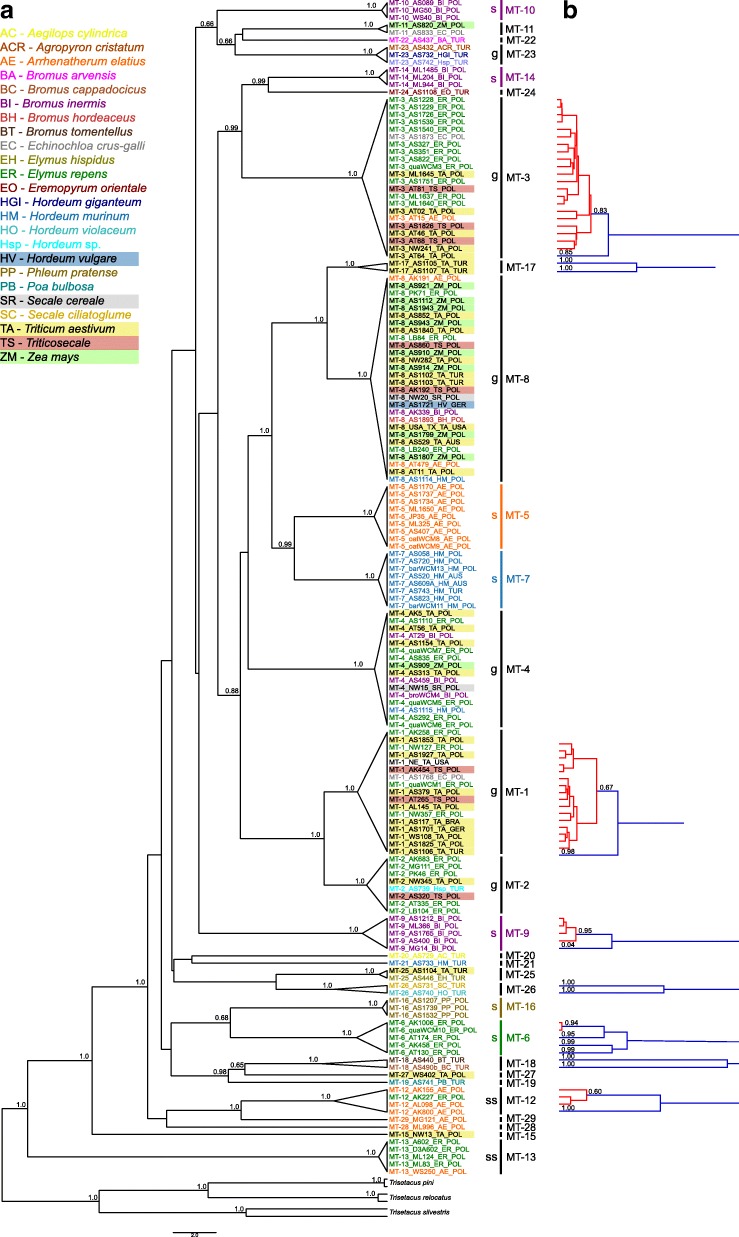
**a** Bayesian inference (BI) tree constructed using the GTR + G model for the cytochrome c oxidase subunit 1 (Cox1) sequences of the wheat curl mite (WCM) species complex and outgroup species. WCM individuals on the tree are colored according to their grass host species; color boxes correspond to cultivated grasses (cereals) and color labels correspond to non-cultivated grasses. Sequence labels correspond to data in Additional file 1: Table S1and include information about the host species and geographic locality. Vertical bars indicate particular WCM lineages, and these of strict specialists (s) are colored according to their specific host plants. Lineages associated with several hosts are designated as generalists (g), and lineages found on two hosts with higher prevalence on one of them are designated as semi-specialists (ss). Numbers above branches are Bayesian posterior probabilities; only values > 0.6 are shown. **b** Results of combined Poisson Tree Processes model for species delimitation (PTP) and a Bayesian implementation of PTP (bPTP) identifying additional putative species groups within MT-1, MT-3, MT-6, MT-9, MT-12, MT-17 MT-18, and MT-26 lineages. Blue lines indicate the unique species groups and red clades indicate species groups contained more than one haplotypes. Numbers above the branches are Bayesian posterior probabilities

The Bayesian Inference (BI) analysis WCM Cox1 sequences revealed a high level of genetic structuring with strong statistical support (posterior probability, PP > 0.95) for the monophyly of 20 major clades (hereafter lineages) and nine lineages represented by single sequences (Fig. 1a and Additional file 2: Figure S1). Trees produced by Maximum Likelihood analysis showed consistent topologies (Additional file 2: Figure S2). Uncorrected pairwise distances between WCM lineages ranged from 6.7 to 28.2% (Additional file 3: Table S2). Seven clades were specialists being associated with a single wild grass species, including lineages associated with smooth-brome (clades: MT-9, MT-10, MT-14, violet color on the Fig. 1a), wall barley (MT-7, light blue), tall oat-grass (MT-5, orange), quackgrass (MT-6, green) and timothy-grass (MT-16, brown) hosts. Two lineages were found on two host species but were mainly associated with one of them, hereafter named ‘semi-specialists’; these included MT-12 and MT-13 (black). Finally, six lineages were found on multiple host species (black, MT-1, MT-2, MT-3, MT-4, MT-8, MT-23), hereafter named as ‘generalists’. Note, that this classification was applied only for lineages with at least three records. The relationships among clades were largely resolved (PP > 0.95). Furthermore, evidence of paraphyly in mites which are specialized toward a particular host was observed, such as in mites associated with smooth brome (i.e. MT-9, MT-10 and MT-14). In contrast, the specialist lineages MT-5 and MT-7 each associated with a different host species (tall oat-grass and wall barley, respectively) formed a well-supported sister-relationship (PP > 0.95). There was also no support for genetic grouping for lineages associated with cereal or wild growing grass hosts, however lineages occurring on cereals were the most polyphagous. Additionally, WCM lineages associated with taxonomically related plant species, namely congeneric species, did not group together (Fig. 1a).

**Fig. 2:**
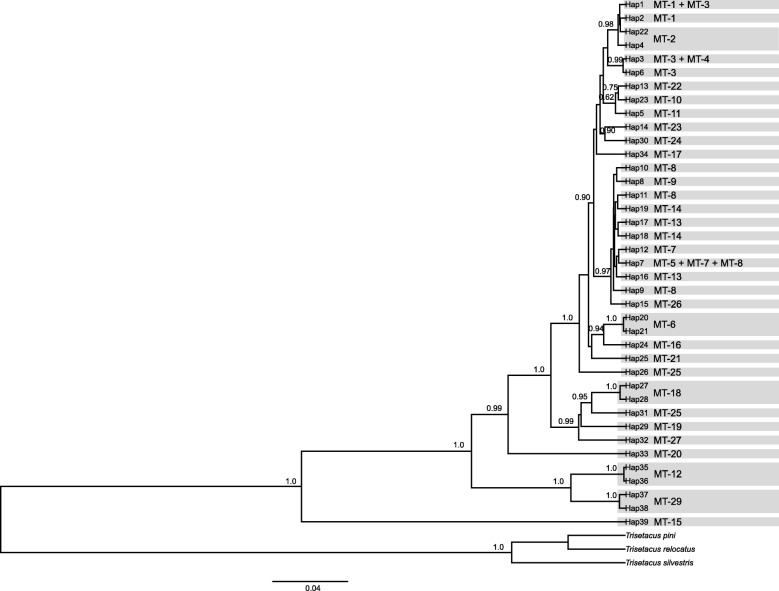
Bayesian inference (BI) tree constructed using the GTR + G model for the haplotypes of 28S rDNA D2 region of the wheat curl mite (WCM) species complex and outgroup species. Numbers above branches are Bayesian posterior probabilities; only values > 0.6 are shown

BI reconstructions of WCM interrelationships based on the D2 dataset (Fig. 2) supported high level of genetic structuring within WCM, however it failed to support some lineage sorting observed in mtDNA analysis. The D2 tree was only partly coinciding with the mitochondrial tree (ILD tests indicated phylogenetic incongruence between the Cox1 and D2, *P* = 0.01): some relationships were similar to those observed in the Cox1 gene tree (e.g. clustering of MT-6 and MT-16; MT-12 and MT-29 with PP > 0.95), but some others were not supported on D2 tree (e.g. between lineages MT-3, MT-13 and MT-24; MT-8 and MT-17). Branch lengths were considerably shorter compared to those within the Cox1 gene tree, but expected due to the comparatively slower evolutionary rate of the locus [71]. The concatenated gene tree, that included a total of 84 unique haplotypes, recovered the major WCM clades established by the mitochondrial tree (PP > 0.95) (Additional file 2: Figure S3).

**Fig. 3:**
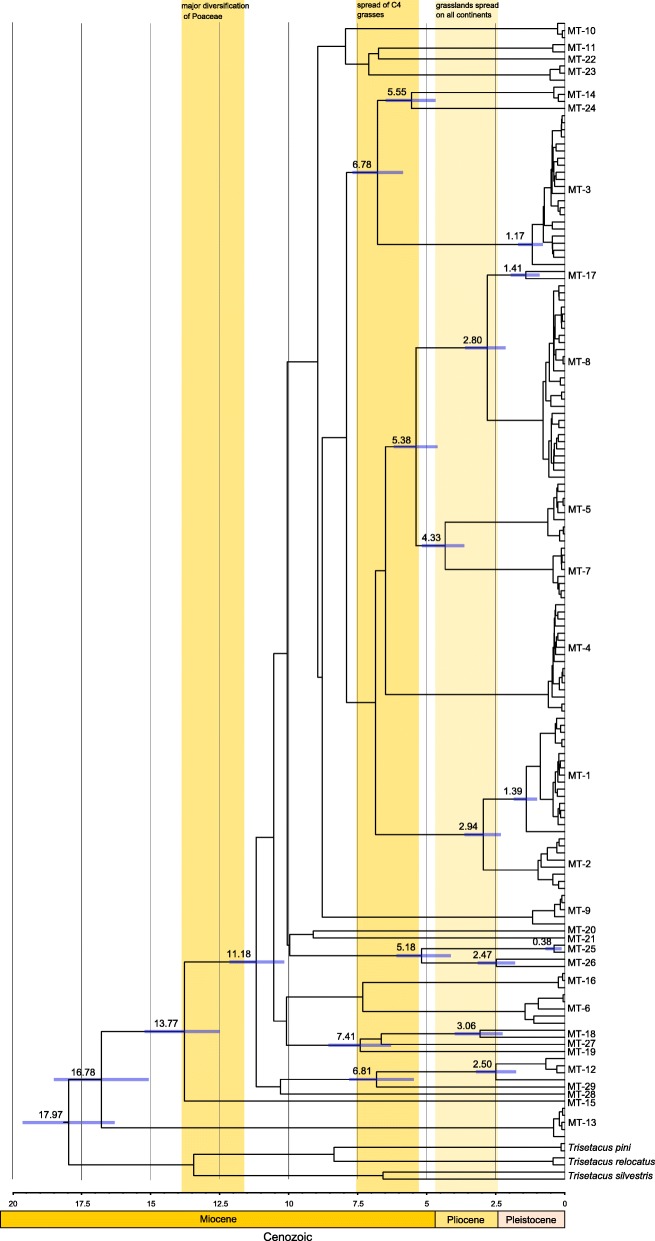
Time calibrated mitochondrial Cox1 gene tree showing mean values in million years (mya) above branches and the 95% HPD bars for divergence time on nodes. Color boxes indicate events that may explain the diversification and divergence of WCM lineages

Our time calibrated Cox1 phylogeny indicates that the tMRCA for all genetic lineages described in this study is approximately 17.97 mya (16.27–19.60 mya). Two of the most genetically distinct WCM lineages (viz. MT-13 and MT-15) diverged approximately 16.78 mya (15.07–18.51 mya) and 13.77 mya (12.50–15.21 mya). Other WCM lineages appear to have started to radiate approximately 11.18 mya (10.15–12.14 mya) in mid-Miocene epoch and continued diversification after ca. 7.5 mya. Interestingly, two of the most polyphagous lineages, associated mostly with cereals (viz. MT-1 and MT-8), diverged approximately at the same time from different ancestors, i.e. ca. 3 mya (2.13–3.62 mya). Two specialist lineages (MT-5 and MT-7) shared a common ancestor with generalists MT-8 at 5.38 mya (4.60–6.19 mya), and they both diverged from their common ancestor at 4.33 mya (3.64–5.17 mya) (Fig. 3). Nevertheless, the above ages of radiation events within the WCM complex should be treated with caution due to the large 95% HPDs.

#### DNA sequence-based species delineation

Species delineation analyses (GYMC and PTP/bPTP methods) (Fig. 1b) were consistent with phylogenetic structuring by identifying each of the 20 major WCM clades as putative species. Additional delineation was detected within eight lineages (MT-1, MT-3, MT-6, MT-9, MT-12, MT-17, MT-18, and MT-26) including specialists, generalists and semi-specialists, in total identifying 45 (41–50) lineages (PTP/bPTP analysis) and 42 (95% CI: 39–50) lineages (GYMC analysis). Lineages assignments were highly consistent between the two methods; however, due to the varying degree of differentiation between lineages, only those identified by PTP/bPTP are shown in Fig. 1b as this is accepted as the most accurate measure when evolutionary distances between lineages are small [65]. Interestingly, lineages within MT-26 and MT-18, both from Turkey, were associated with different grass species. Within the globally distributed MT-1 lineage, one distinct lineage was collected in Turkey, and the second one comprised mites from multiple countries including Poland, USA, Brazil, and Germany. Analyses also identified four distinct lineages within the specialist quackgrass-associated MT-6 lineage in Poland.

### Diversity within WCM lineages and tests for demographic history

The majority of the WCM lineages had high Cox1 haplotype diversity (*H*_*d ≥*_ 0.7), with exception of the MT-13 lineage which had *H*_*d*_ *=* 0.5256. Lineage MT-29 had a haplotype diversity of 0.000; however, this estimate is likely compromised by the small sample size (*N* = 2). Nucleotide Cox1 diversity (*л*) ranged from 0 to 0.0696. Haplotype and nucleotide D2 diversity was in most cases lower when compared to that of Cox1 (Additional file 3: Table S3).

Tajima’s D and Fu’s Fs analyses were negative and significant for the MT-3, MT-4, and MT-8 lineages, and Fu’s Fs was also significantly negative for the MT-5 lineage (Table 1), indicating deviation from neutrality. Mismatch distributions for the WCM lineages MT-1, MT-2, MT-3, MT-4, MT-5, and MT-8 were unimodal with a significant sudden expansion model fit and non-significant raggedness test values (Table 1 and Additional file 4: Figure S4). Whereas, the sudden expansion model was rejected for the MT-7 and MT-9 lineages (Table 1 and Additional file 4: Figure S4).

**Table 1:** Demographic analyses and recent expansion time for WCM lineages (for those with *N* ≥ 15). The estimates of neutrality tests for each lineage were determined by Tajima’s *D* and Fu’s Fs statistics. The validity of sudden expansion model was tested by mismatch distributions: SSD, the sum of square deviations; *H*_Rag_, the raggedness index

WCM lineage	Tajima’s D	Fu’s Fs	SSD (*P*-value)	*H*_Rag_ (P-value)	*expansion time*
MT-1	−1.3236 (0.0760)	−3.7391 (0.0730)	0.0293 (0.062)	0.0428 (0.246)	27,000
MT-2	1.4104 (0.0650)	−1.0015 (0.2720)	0.0332 (0.223)	0.0406 (0.427)	290,000
MT-3	**−2.2957 (0.0000)**	**−25.937 (0.0000)**	0.0003 (0.910)	0.0145 (0.724)	160,000
MT-4	**−2.1122 (0.0030)**	**−8.3451 (0.0000)**	0.0047 (0.901)	0.0231 (0.948)	165,000
MT-5	−0.8571 (0.2170)	**−10.933 (0.0010)**	0.0029 (0.545)	0.0117 (0.651)	440,000
MT-7	1.5945 (0.9500)	2.1540 (0.8730)	**0.0951 (0.038)**	0.2901 (0.063)	390,000
MT-8	**−2.2603 (0.0000)**	**−26.635 (0.0000)**	0.0024 (0.279)	0.0298 (0.298)	86,000
MT-9	1.0643 (0.8900)	1.4746 (0.3410)	**0.0301 (0.005)**	0.0144 (0.186)	995,000

*P* < 0.05 are bolded; all others are not significant

### Genetic structure in multi-host WCM lineages

Haplotype networks for multi-host WCM lineages did not reveal clear host associated structure (Additional file 5: Figures S5-S9). Generalist WCM lineages MT-1, MT-3, and MT-8 had complex networks, showing star-like patterns. Common ancestral haplotypes of MT-1, MT-3 and MT-8 were polyphagous, being present in many hosts (Additional file 5: Figures S5, S7, S9). The majority of MT-1 (65.3%) and MT-8 (75.6%) haplotypes were associated with cereals, whereas the majority of MT-3 haplotypes (76.4%) were quackgrass-associated. Also, the most common ancestral haplotype of lineage MT-3 (h1, 28.5% frequency) was mainly associated with quackgrass (frequency 64.8%) (Additional file 5: Figure S7). The MT-2 network (Additional file 5: Figure S6) revealed a well resolved haplotype structure, lacking a common ancestral haplotype, and displaying two highly divergent haplotypes separated by more than 10 mutations from the main haplotype cluster. In the MT-4 lineage haplotype network (Additional file 5: Figure S8), the most common central haplotype (h2) was associated with five hosts.

Analysis of molecular variance partitioned by host (Table 2) revealed non-significant subdivision for WCM lineages MT-1 (− 5.01, *P* = 0.67644 + − 0.01490), MT-3 (1.69, *P* = 0.14761 + − 0.01375), and MT-4 (7.70, *P* = 0.29228 + − 0.01517). Variation among different host-associated groups was moderate but significant in MT-8 (6.90, *P* = 0.00587 + − 0.002600), and significant and high in MT-2 (44.13, *P* = 0.04301 + − 0.00576), however in the latter one results were based on small sample size.

**Table 2:** Analyses of molecular variance (AMOVA) for generalist WCM lineages with host as a grouping variable

Source of variation	d.f.	Sum of squares	Variance components	% variation	F_ST_
MT-1					
Among hosts	7	14.91	−0.13294	−5.01	
Within hosts	41	113.46	2.76733	105.01	−0.0501
Total	48	128.37	2.63539		
MT-2					
Among hosts	3	21.70	2.42727	44.13	
Within host	10	30.73	3.07273	55.87	0.44132
Total	13	52.43	5.50000		
MT-3					
Among hosts	7	14.01	0.02645	1.69	
Within host	311	479.58	1.54205	98.31	0.01686
Total	318	493.59	1.56850		
MT-4					
Among hosts	7	10.27	0.09861	7.70	
Within host	23	27.20	1.18244	92.30	0.07697
Total	30	37.45	1.28104		
MT-8					
Among hosts	10	23.88	0.09467	6.90	
Within host	154	196.68	1.27714	93.10	0.06901
Total	164	220.56	1.37181		

*d.f* deegres of freedom, *F*_*ST*_ Fixation index

Most pairwise F_ST_ values between host-associated groups within the generalist MT-1, MT-3, MT-8 lineages did not differ significantly from zero, indicating gene flow and shared genetic diversity between lineages. In contrast, F_ST_ between triticale- and maize-associated MT-8 lineages was significantly different from zero (F_ST_ = 0.53; *P* < 0.01) indicating an absence of gene flow (Additional file 3: Table S4).

Haplotype diversity of generalist lineages and host-associated groups within these lineages was high in the majority of cases (Additional file 3: Table S5), and the exceptions with lower *H*_*d*_ values refer to groups with very low Ns.

Comparing the nucleotide diversity of host-associated populations with the whole ancestral lineage, values were similar only for MT-2 and MT-8 lineages, with exception of the MT-8 oats-associated population which had lower nucleotide diversity. For MT-1 lineage only the wheat-associated population had comparable nucleotide diversity to that observed for the whole MT-1 lineage. Other cereal-associated populations had lower nucleotide diversity when compared to the whole MT-1 lineage, whereas, on the contrary, quackgrass-associated population had higher nucleotide diversity than MT-1 lineage. Within MT-3 lineage, barley- and smooth brome-associated populations had lower nucleotide diversity, while others (wheat-, triticale- and quackgrass-associated) had comparable values to that observed for the whole MT-3 lineage. Wheat-associated MT-4 population had much lower nucleotide diversity when compared to the values observed for the whole lineage and the two wild grass-associated populations (quackgrass and smooth brome) (Additional file 3: Table S5).

## Discussion

Results from this study provide new and important insights into the extent and timing of lineage diversification, and the diversity of host usage in the economically important mite taxon, which is recognized as one of the most prolific and damaging pests of cereal crops around the world [72]. Being a complex of genetic lineages with diverse patterns of host usage, it is an ideal model system for studying the mechanisms underlying the diversification processes.

### WCM complex differentiation

The phylogenetic reconstructions performed in this study confirmed deep lineage diversification within the wheat curl mite, indicating a complex of lineages more diverse than previously assumed [36–38]. We identified 29 divergent genetic lineages, with their mitochondrial Cox1 locus differing by 6.7 to 28.2% (uncorrected p-distance), which corresponds or exceeds levels of interspecific variation observed in other animal taxa [27, 73–75]. Moreover, within eight of these lineages additional genetic sub-structuring was detected, suggesting that the WCM could be a complex of more than 40 distinct lineages. It is likely that lineage diversity remains grossly underestimated, there is a need for more extensive global investigations of WCM species complex. This presents a significant challenge for biodiversity assessments and cereal crop protection, as distinct lineages are expected to be biologically different and vary in their ability to vector plant pathogens [37]. Therefore, future studies and control measures will need to account for biological and ecological differences between WCM lineages.

Although the criticism toward mitochondrial COI as a barcode for identifications and as a sequence for measuring divergence of closely related species exists (e.g. [76, 77]) COI reference data availability enables distance comparisons between case studies. Some of the advantages of employing COI for our study are that: (i) it is comparable to data from other eriophyoid mite species and distinguishes them well (e.g. [78, 79]); (ii) it is relatively easy to obtain from even scarce DNA amount extracted out of as little as one specimen [80]; (iii) its resolution is high enough to work on genus, species and population level [80]; (iv) there are known closely related *Aceria* species that do not exhibit such a high variation in COI as WCM, i.e. *A. tulipae* [81], and this supports notion that the variation seen in WCM is related to their overall genetic divergence, and that WCM is not a single evolutionary unit.

We identified some discrepancies in lineage delineation between the mitochondrial and nuclear DNA datasets, a phenomenon frequently reported in many animal taxa [82–86]. The uniparental inheritance and smaller effective population size of mitochondrial DNA suggests that lineage sorting will occur faster in mitochondrial DNA than in nuclear DNA, rendering the locus more reliable for characterizing recent lineage divergences [84].

### Diversity in host use in relation to phylogenetic structuring

Our research has characterized WCM as a complex of at least seven specialist, six generalist, and two semi-specialist taxa, with specificity levels yet to be assigned for several other distinct genetic lineages. We expect that semi-specialist lineages MT-12 and MT-13 are in fact host specialists, given they occur predominantly on single grass host species, and only sporadically on other hosts. This is in concordance with previous research on host-related variation in population density of WCM lineages, which has indicated that MT-12 and MT-13 exhibit preferences toward one host species (tall oat-grass and quackgrass, respectively), although the lineages have been found on the other hosts sporadically [42].

Our phylogenetic reconstructions also indicate that neither host generalization nor specialization is a phylogenetically constrained trait, as the tree topology provides clear evidence of their convergent evolution in unrelated taxa. Shifting patterns of host generalization to specialization between closely-related lineages suggest that host range expansion and contraction is likely to be dynamic through time. The phenomenon has been well documented and can be attributed to environmental changes, including anthropogenic influence [10, 12]. Although it is not clear which factors have influenced diversification of WCM, it can be hypothesized that diversity in host use within WCM complex had been linked to vegetation transitions during the Miocene and Pliocene (discussed in next section), and differences in dispersal and colonization abilities between taxa [87, 88].

Related phytophagous insect species tend to feed on plants from similar taxonomic groupings [89–92]. However, this does not appear to apply to WCM, as evidence of phylogenetic structuring of mite lineages associated with host specificity or host taxonomy was not detected. While this is an uncommon phenomenon, it has been observed previously in butterfly *Vanessa cardui* [93], leaf beetles [89] and monogeneans parasitizing fish [94]. These findings provide new insights into the diversity of host usage, however greater sampling of WCM from a broader range of grass hosts and across multiple continents is needed to gain a more reliable appreciation of absolute host ranges for WCM lineages.

### Timing of WCM lineages diversification

WCM is inherently associated with grass hosts which are intensively farmed across multiple continents. Therefore, the global distribution of WCM has been previously attributed to the extension of agriculture around the globe [39], and here we hypothesized that agricultural practices are also likely to have had an influence on lineage diversity. However, our time calibrated phylogeny suggests that WCM radiations pre-date the origin and expansion of global agriculture, which originated approximately 11,000 year ago in the Near East [95]. Instead, we have shown that mite lineages diverged between 16.5 mya and 19 mya, which overlaps with the timing of major diversification events within Poaceae, including ecological shifts from heavy forestation to savannas during the mid-Miocene (Fig. 3) [96, 97]. The timing of additional divergence also coincides with important events in the history of the grass family, including the expansion of C4 grasses between 7.5 and 5.5 mya, and the worldwide spread of grasslands between ca. 4.0 and 2.5 mya [97–99]. The divergence of cereal-associated MT-1 and MT-8 generalist appears to correspond to the final phase of global grasslands spread around 3 mya, long before the development of agriculture. Therefore, we can conclude that while agricultural practices may have had a significant influence on WCM distributions, these activities have had no detectable impact on WCM mite lineage diversification, and instead this appears to be closely linked with the early diversification of grass hosts.

### Patterns of WCM lineages demographic expansion

Our analyses suggest that some generalist WCM lineages have undergone major historical demographic expansion, but again the timing of these events precedes the potential influence of agriculture. Generalist lineages associated with wild grasses appear to have underwent much earlier demographic expansions during the Pleistocene 160,000–290,000 years ago. In contrast, the most widespread generalists MT-1 and MT-8, which nowadays predominate on cereal hosts, appear to have undergone expansion events more recently, approximately 27,000 and 86,000 years ago, respectively. It is likely that during the time of recent expansion MT-1 and MT-8 lineages infested wild grasses, since many cereal hosts are no older than 11,000 years old [100]. It is possible that these lineages were associated with progenitors of cereals, e.g. quackgrass-related plants, and had some intrinsic characteristics for rapid colonization and proliferation [29].

In contrast, the demographic history of specialists MT-7 (associated with wall-barley) and MT-9 (associated with smooth brome) appears to have been more stable with evidence of expansion events predating all generalist lineages (approximately 390,000 and 950,000 years ago, respectively). Expansions seem to be more likely in generalists rather than in specialists, probably given their enhanced dispersal capacities and abilities to capitalize on host availability on greater geographic scales [24, 101].

### Host-associated structuring within WCM generalist lineages

High host-specificity is a prevalent feeding strategy in eriophyoid mites [102], therefore we expected to observe host-associated structuring in generalist WCM lineages, indicating incipient speciation processes. However, we found no evidence of these within generalist lineages MT-1, MT-3, and MT-4. In contrast, some host structuring was detected within the MT-8 lineage, with an apparent differentiation between mites from triticale and maize hosts, suggesting limited gene flow only between these two host-associated populations. However, this general absence of host-associated genetic structure within host generalist lineages indicates ongoing gene flow assisted by high dispersal and rapid colonization potential [29, 103], and suggests that generalist WCM lineages may be capable of extending their host ranges. These results are consistent with previous observations that MT-1 and MT-8 lineages are the most pestiferous within the WCM complex [39]. This has significant implications for future pest control in cereal crops, as over-summering grass hosts provide green bridge refuges for mite persistence and re-infestation of emerging crops.

## Conclusions

Our research focuses on patterns of genetic diversity and host association in obligatory phytophagous wheat curl mite (WCM), which is a major agricultural pest in cropping regions of the world. We revealed that lineage diversity within the taxon is significantly greater than was assumed, and consists of at least 29 genetically divergent lineages with distinctive host-use patterns, ranges from host generalists to specialists. The timing of WCM diversification events pre-dates agriculture, instead appears to be associated with timing of global diversification events within Poaceae during the mid-Miocene and the Pliocene. These novel findings provide insight into co-evolutionary processes among herbivores and their hosts, and revoke hypothesis that WCM lineage diversity is primarily linked to human activity, similarly like it has been shown for whitefly *Bemisia tabaci* [104].

Furthermore, our results shed new light on the mechanisms associated with specialization and generalization, each appearing to have evolved convergently in unrelated WCM lineages on numerous occasions. Such a pattern supports the notion that host use strategy can be highly dynamic in herbivores [11, 105], although host specificity is also known to be phylogenetically constrained in phytophagous insects [106] and in animal parasites [107]. The demographic histories of generalist mite lineages also appear to be dynamic, with evidence suggesting that many have undergone major demographic expansion events, with ongoing gene flow among populations from different hosts. This is consistent with our knowledge of generalist lineages being the most prolific and damaging in cereal cropping regions of the world. Overall, we demonstrate that WCM diversity is associated with diversity in host use, which is likely to be affected by a complex of factors, including invertebrate life history traits influencing dispersal and colonization abilities, and processes such as secondary contact, host plant diversification, and environmental changes.

## Additional files

Additional file 1: Sequence and sampling information. (DOCX 99 kb)

Additional file 2: Phylogenetic trees for wheat curl mite sequence datasets. (DOCX 776 kb)

Additional file 3: Statistics for genetic diversity of WCM genetic lineages. (DOCX 738 kb)

Additional file 4: Mismatch distributions of WCM genetic lineages. (DOCX 747 kb)

Additional file 5: Haplotype networks for the mtDNA Cox1 wheat curl mite (WCM). (DOCX 950 kb)

## Abbreviations

AIC: Akaike Information Criteria
AMOVA: Analysis of molecular variation
BI: Bayesian inference
bPTP: Bayesian implementation of PTP
Cox1: Cytochrome c oxidase subunit I
d.f.: Deegres of freedom
ESSs: Effective sample sizes
F_ST_: Fixation index
GMYC: General mixed Yule coalescent
*h*: Number of haplotypes
*H*_*d*_: Haplotype diversity
HPD: Highest posterior density
H_Rag_: Raggedness index
MCC: Maximum clade credibility
MCMC: Markov Chain Monte Carlo
ML: Maximum likelihood
*p*: Number of polymorphic sites
PTP: Poisson tree processes model for species delimitation
SSD: Observed and expected mismatch
TBR: Tree bisection-reconnection
tMRCAs: Recent common ancestor
WCM: Wheat curl mite, *Aceria tosichella*
*л*: Nucleotide diversity
