# Supplementary material for: Genetics of lineage diversification and the evolution of host usage in the economically important wheat curl mite, Aceria tosichella Keifer, 1969
Source: BMC Evol Biol. 2018 Aug 7;18:122. doi: 10.1186/s12862-018-1234-x (PMC6081818; doi:10.1186/s12862-018-1234-x)
Supplement: Supplementary file 1 — Sequence and sampling information. (DOCX 99 kb) [file 12862_2018_1234_MOESM1_ESM.docx]

**Additional file 1:** Sequence and sampling information

**Table S1** Sequence and sampling information: WCM lineage, sequence ID, host plant species, locality - country, GPS coordinators, GenBank Accession numbers (sequences obtained from GenBank are marked with an asterisk).

| **No.** | **lineage** | **ID sequence COI** | **ID sequence D2** | **host species latin** | **host species common name** | **locality** | **Coordinates** | **Acc. Number COI** | **Acc. Number D2** |
| --- | --- | --- | --- | --- | --- | --- | --- | --- | --- |
| 1 | MT-1 | MT-1_AL145_TA_POL |  | *Triticum aestivum* | wheat | Poland | 50°07'25"N, 17°42'20"E | MG193929 |  |
| 2 | MT-1 | MT-1_WS108_TA_POL |  | *Triticum aestivum* | wheat | Poland | 50°56'31"N, 23°18'28"E | MG193930 |  |
| 3 | MT-1 | MT-1_WS130_TA_POL |  | *Triticum aestivum* | wheat | Poland | 50°56'31"N, 23°18'28"E | MG193931 |  |
| 4 | MT-1 | MT-1_AS379_TA_POL | MT-1_AS379.D2_TA_POL | *Triticum aestivum* | wheat | Poland | 52°02'36"N, 16°46'02"E | JF920077* | JF920097* |
| 5 | MT-1 | MT-1_NW383_TA_POL |  | *Triticum aestivum* | wheat | Poland | 50°07'01"N, 17°57'27"E | MG193932 |  |
| 6 | MT-1 | MT-1_AS1927_TA_POL |  | *Triticum aestivum* | wheat | Poland | 52°39'11"N, 17°53'54"E | MG193933 |  |
| 7 | MT-1 | MT-1_AS1924_TA_POL |  | *Triticum aestivum* | wheat | Poland | 52°39'11"N, 17°53'54"E | MG193934 |  |
| 8 | MT-1 | MT-1_AS1825_TA_POL | MT-1_AS1825.D2_TA_POL | *Triticum aestivum* | wheat | Poland | 50°56'31"N, 23°18'28"E | MG193935 | MG076809 |
| 9 | MT-1 | MT-1_AS1853_TA_POL | MT-1_AS1853.D2_TA_POL | *Triticum aestivum* | wheat | Poland | 52°02'36"N, 16°46'02"E | MG193936 | MG076810 |
| 10 | MT-1 | MT-1_AK651_TA_POL |  | *Triticum aestivum* | wheat | Poland | 50°19'40"N, 20°30'39"E | MG193937 |  |
| 11 | MT-1 | MT-1_ML1691_TA_POL |  | *Triticum aestivum* | wheat | Poland | 50°19'40"N, 20°30'39"E | MG193938 |  |
| 12 | MT-1 | MT-1_AT225_TA_POL |  | *Triticum aestivum* | wheat | Poland | 51°42'35"N, 15°18'02"E | MG193939 |  |
| 13 | MT-1 | MT-1_NW33_TA_POL |  | *Triticum aestivum* | wheat | Poland | 51°53'26"N, 15°19'45"E | MG193940 |  |
| 14 | MT-1 | MT-1_WS201_TA_POL |  | *Triticum aestivum* | wheat | Poland | 53°07'27"N, 14°33'60"E | MG193941 |  |
| 15 | MT-1 | MT-1_AK454_TS_POL |  | *Triticosecale* | triticale | Poland | 53°08'42"N, 15°09'47"E | MG193942 |  |
| 16 | MT-1 | MT-1_AT265_TS_POL | MT-1_AT265.D2_TS_POL | *Triticosecale* | triticale | Poland | 51°19'50"N, 17°09'18"E | MG193943 | MG076811 |
| 17 | MT-1 | MT-1_ML1666_TS_POL |  | *Triticosecale* | triticale | Poland | 50°41'50"N, 20°28'48"E | MG193944 |  |
| 18 | MT-1 | MT-1_ML1663_TS_POL |  | *Triticosecale* | triticale | Poland | 50°23'45"N, 21°18'04"E | MG193945 |  |
| 19 | MT-1 | MT-1_AK474_TS_POL |  | *Triticosecale* | triticale | Poland | 50°22'44"N, 22°22'11"E | MG193946 |  |
| 20 | MT-1 | MT-1_AK52_HV_POL | MT-1_AK52.D2_HV_POL | *Hordeum vulgare* | barley | Poland | 52°29'38"N, 15°22'48"E | KP973942* | MG076812 |
| 21 | MT-1 | MT-1_WS297_HV_POL |  | *Hordeum vulgare* | barley | Poland | 52°39'11"N, 17°53'54"E | MG193947 |  |
| 22 | MT-1 | MT-1_ML534_ER_POL |  | *Elymus repens* | quackgrass | Poland | 52°57'11"N, 21°10'53"E | MG193948 |  |
| 23 | MT-1 | MT-1_AK258_ER_POL |  | *Elymus repens* | quackgrass | Poland | 50°57'04"N, 17°03'22"E | MG193949 |  |
| 24 | MT-1 | MT-1_AK274_ER_POL |  | *Elymus repens* | quackgrass | Poland | 51°32'49"N, 17°11'22"E | MG193950 |  |
| 25 | MT-1 | MT-1_AL130_ER_POL |  | *Elymus repens* | quackgrass | Poland | 50°07'25"N, 17°42'20"E | MG193951 |  |
| 26 | MT-1 | MT-1_NW127_ER_POL |  | *Elymus repens* | quackgrass | Poland | 50°54'25"N, 23°57'34"E | MG193952 |  |
| 27 | MT-1 | MT-1_NW357_ER_POL |  | *Elymus repens* | quackgrass | Poland | 50°07'01"N, 17°57'27"E | MG193953 |  |
| 28 | MT-1 | MT-1_NW359_ER_POL |  | *Elymus repens* | quackgrass | Poland | 50°07'01"N, 17°57'27"E | MG193954 |  |
| 29 | MT-1 | MT-1_PK68_ER_POL |  | *Elymus repens* | quackgrass | Poland | 51°32'49"N, 17°11'22"E | MG193955 |  |
| 30 | MT-1 | MT-1_AS361_ER_POL |  | *Elymus repens* | quackgrass | Poland | 51°57'30"N, 16°15'13"E | MG193956 |  |
| 31 | MT-1 | MT-1_PK85_ER_POL |  | *Elymus repens* | quackgrass | Poland | 52°39'11"N, 17°53'54"E | MG193957 |  |
| 32 | MT-1 | MT-1_AT209_ER_POL |  | *Elymus repens* | quackgrass | Poland | 51°56'09"N, 14°48'05"E | MG193958 |  |
| 33 | MT-1 | MT-1_PK2_ER_POL |  | *Elymus repens* | quackgrass | Poland | 49°42'27"N, 19°17'54"E | MG193959 |  |
| 34 | MT-1 | MT-1_AS1768_EC_POL | MT-1_AS1768.D2_EC_POL | *Elymus repens* | quackgrass | Poland | 51°50'57"N, 17°10'24"E | MG193960 | MG076813 |
| 35 | MT-1 | MT-1_quaWCM1_ER_POL | MT-1_quaWCM1.D2_ER_POL | *Elymus repens* | quackgrass | Poland | 52°27'55"N, 16°55'55"E | JQ248913* | JQ918879* |
| 36 | MT-1 | MT-1_broWCM1_BI_POL | MT-1_broWCM1.D2_BI_POL | *Bromus inermis* | smooth brome | Poland | 52°28'03"N, 16°54'31"E | JQ248920* | JQ918883* |
| 37 | MT-1 | MT-1_oatWCM1_AE_POL | MT-1_oatWCM1.D2_AE_POL | *Arrhenatherum elatius* | tall oat-grass | Poland | 52°28'03"N, 16°54'31"E | JQ248921* | JQ918884* |
| 38 | MT-1 | MT-1_AS1701_TA_GER | MT-1_AS1701.D2_TA_GER | *Triticum aestivum* | wheat | Germany | 51°47'02"N, 11°17'60"E | MG193961 | MG076814 |
| 39 | MT-1 | MT-1_AS1714_HV_GER | MT-1_AS1714.D2_HV_GER | *Hordeum vulgare* | barley | Germany | 51°47'02"N, 11°17'60"E | MG193962 | MG076815 |
| 40 | MT-1 | MT-1_AS1722_HV_GER |  | *Hordeum vulgare* | barley | Germany | 51°47'02"N, 11°17'60"E | MG193963 |  |
| 41 | MT-1 | MT-1_AS1106_TA_TUR | MT-1_AS1106.D2_TA_TUR | *Triticum aestivum* | wheat | Turkey | 40°32'04"N, 43°06'33"E | KC412865* | KM280954* |
| 42 | MT-1 | MT-1_AS117_TA_BRA |  | *Triticum aestivum* | wheat | Brazil | 28°24'30"S, 54°57'41"W | JF920071* |  |
| 43 | MT-1 | MT-1_AS123_TA_BRA | MT-1_AS123.D2_TA_BRA | *Triticum aestivum* | wheat | Brazil | 28°13'60"S, 52°23'60"W | JF920072* | JF920099* |
| 44 | MT-1 | MT-1_AS119_TA_ARG | MT-1_AS119.D2_TA_ARG | *Triticum aestivum* | wheat | Argentina | 36°46'30"S, 59°51'15"W | JF920073* | JF920098* |
| 45 | MT-1 | MT-1_AS476_TA_ARG |  | *Triticum aestivum* | wheat | Argentina | 38°13'59"S, 59°01'02"W | JF920074* |  |
| 46 | MT-1 | MT-1_AS477_TA_ARG |  | *Triticum aestivum* | wheat | Argentina | 33°17'01"S, 62°11'07"W | JF920075* |  |
| 47 | MT-1 | MT-1_AS478_TA_ARG |  | *Triticum aestivum* | wheat | Argentina | 34°13'49"S, 58°54'11"W | JF920076* |  |
| 48 | MT-1 | MT-1_AS607_BC_AUS | MT-1_AS607.D2_BC_AUS | *Bromus cappadocius* | soft brome | Australia | 33°07'16"S, 148°58'32"E | JF920092* | JF920108* |
| 49 | MT-1 | MT-1_NE_TA_USA |  | *Triticum aestivum* | wheat | USA | n/a | JX102055* |  |
| 50 | MT-2 | MT-2_NW345_TA_POL | MT-2_NW345.D2_TA_POL | *Triticum aestivum* | wheat | Poland | 50°29'26"N, 17°32'26"E | MG208676 | MG076816 |
| 51 | MT-2 | MT-2_AS320_TS_POL | MT-2_AS320.D2_TS_POL | *Triticosecale* | triticale | Poland | 52°02'24"N, 16°46'09"E | MG208677 | MG076817 |
| 52 | MT-2 | MT-2_AT335_ER_POL |  | *Elymus repens* | quackgrass | Poland | 50°32'21"N, 23°28'58"E | MG208678 |  |
| 53 | MT-2 | MT-2_MG111_ER_POL |  | *Elymus repens* | quackgrass | Poland | 51°42'35"N, 15°18'02"E | MG208679 |  |
| 54 | MT-2 | MT-2_PK46_ER_POL |  | *Elymus repens* | quackgrass | Poland | 51°32'50"N, 15°00'38"E | KP973941* |  |
| 55 | MT-2 | MT-2_AK683_ER_POL |  | *Elymus repens* | quackgrass | Poland | 51°32'50"N, 15°00'38"E | MG208680 |  |
| 56 | MT-2 | MT-2_AK807_ER_POL |  | *Elymus repens* | quackgrass | Poland | 50°43'02"N, 16°34'11"E | MG208681 |  |
| 57 | MT-2 | MT-2_LB104_ER_POL |  | *Elymus repens* | quackgrass | Poland | 51°18'21"N, 21°13'54"E | MG208682 |  |
| 58 | MT-2 | MT-2_WS236_ER_POL |  | *Elymus repens* | quackgrass | Poland | 52°30'05"N, 14°45'45"E | MG208683 |  |
| 59 | MT-2 | MT-2_ML1136_ER_POL |  | *Elymus repens* | quackgrass | Poland | 53°26'13"N, 22°59'01"E | MG208684 |  |
| 60 | MT-2 | MT-2_ML1737_ER_POL |  | *Elymus repens* | quackgrass | Poland | 53°07'03"N, 22°50'58"E | MG208685 |  |
| 61 | MT-2 | MT-2_ML1749_ER_POL |  | *Elymus repens* | quackgrass | Poland | 52°57'11"N, 21°10'53"E | MG208686 |  |
| 62 | MT-2 | MT-2_quaWCM2_ER_POL | MT-2_quaWCM2.D2_ER_POL | *Elymus repens* | quackgrass | Poland | 52°27'55"N, 16°55'55"E | JQ248914* | JQ918880* |
| 63 | MT-2 | MT-2_AS739_Hsp_TUR |  | *Hordeum* sp. |  | Turkey | 38°10'37"N, 43°56'58"E | KC412855* |  |
| 64 | MT-3 | MT-3_NW241_TA_POL |  | *Triticum aestivum* | wheat | Poland | 51°38'28"N, 19°28'11"E | MG193964 |  |
| 65 | MT-3 | MT-3_ML1645_TA_POL |  | *Triticum aestivum* | wheat | Poland | 51°53'26"N, 15°19'45"E | MG193965 |  |
| 66 | MT-3 | MT-3_AT02_TA_POL | MT-3_AT02.D2_TA_POL | *Triticum aestivum* | wheat | Poland | 50°07'34"N, 16°42'43"E | MG193966 | MG076818 |
| 67 | MT-3 | MT-3_AT46_TA_POL |  | *Triticum aestivum* | wheat | Poland | 50°43'02"N, 16°34'11"E | MG193967 |  |
| 68 | MT-3 | MT-3_AT123_TA_POL |  | *Triticum aestivum* | wheat | Poland | 52°28'57"N, 16°40'48"E | MG193968 |  |
| 69 | MT-3 | MT-3_AK928_TA_POL |  | *Triticum aestivum* | wheat | Poland | 50°43'02"N, 16°34'11"E | MG193969 |  |
| 70 | MT-3 | MT-3_WS258_TA_POL |  | *Triticum aestivum* | wheat | Poland | 49°48'39"N, 22°23'59"E | MG193970 |  |
| 71 | MT-3 | MT-3_AT288_TA_POL |  | *Triticum aestivum* | wheat | Poland | 54°27'54"N, 16°53'36"E | MG193971 |  |
| 72 | MT-3 | MT-3_AT344_TA_POL |  | *Triticum aestivum* | wheat | Poland | 52°44'22"N, 16°56'52"E | MG193972 |  |
| 73 | MT-3 | MT-3_NW246A_TA_POL |  | *Triticum aestivum* | wheat | Poland | 50°50'41"N, 20°08'38"E | MG193973 |  |
| 74 | MT-3 | MT-3_NW255_TA_POL |  | *Triticum aestivum* | wheat | Poland | 50°41'50"N, 20°28'48"E | MG193974 |  |
| 75 | MT-3 | MT-3_NW247_TA_POL |  | *Triticum aestivum* | wheat | Poland | 50°39'25"N, 20°01'34"E | MG193975 |  |
| 76 | MT-3 | MT-3_NW264_TA_POL |  | *Triticum aestivum* | wheat | Poland | 51°12'33"N, 18°29'30"E | MG193976 |  |
| 77 | MT-3 | MT-3_NW281_TA_POL |  | *Triticum aestivum* | wheat | Poland | 52°44'43"N, 18°10'39"E | MG193977 |  |
| 78 | MT-3 | MT-3_NW291_TA_POL |  | *Triticum aestivum* | wheat | Poland | 53°44'14"N, 18°53'11"E | MG193978 |  |
| 79 | MT-3 | MT-3_NW292_TA_POL |  | *Triticum aestivum* | wheat | Poland | 53°21'01"N, 18°43'19"E | MG193979 |  |
| 80 | MT-3 | MT-3_NW323_TA_POL |  | *Triticum aestivum* | wheat | Poland | 50°29'08"N, 17°55'17"E | MG193980 |  |
| 81 | MT-3 | MT-3_NW343_TA_POL |  | *Triticum aestivum* | wheat | Poland | 50°29'26"N, 17°32'26"E | MG193981 |  |
| 82 | MT-3 | MT-3_NW391_TA_POL |  | *Triticum aestivum* | wheat | Poland | 50°05'35"N, 18°22'40"E | MG193982 |  |
| 83 | MT-3 | MT-3_WS458_TA_POL |  | *Triticum aestivum* | wheat | Poland | 50°49'21"N, 18°32'18"E | MG193983 |  |
| 84 | MT-3 | MT-3_ML1694_TA_POL |  | *Triticum aestivum* | wheat | Poland | 50°29'56"N, 18°38'25"E | MG193984 |  |
| 85 | MT-3 | MT-3_ML496A_TA_POL |  | *Triticum aestivum* | wheat | Poland | 52°08'09"N, 21°05'51"E | MG193985 |  |
| 86 | MT-3 | MT-3_ML500_TA_POL |  | *Triticum aestivum* | wheat | Poland | 52°08'09"N, 21°05'51"E | MG193986 |  |
| 87 | MT-3 | MT-3_ML899_TA_POL |  | *Triticum aestivum* | wheat | Poland | 53°50'17"N, 20°18'54"E | MG193987 |  |
| 88 | MT-3 | MT-3_ML1553_TA_POL |  | *Triticum aestivum* | wheat | Poland | 53°50'17"N, 20°18'54"E | MG193988 |  |
| 89 | MT-3 | MT-3_ML1559_TA_POL |  | *Triticum aestivum* | wheat | Poland | 51°21'60"N, 22°26'31"E | MG193989 |  |
| 90 | MT-3 | MT-3_ML1079_TA_POL |  | *Triticum aestivum* | wheat | Poland | 51°54'26"N, 22°24'37"E | MG193990 |  |
| 91 | MT-3 | MT-3_ML1241_TA_POL |  | *Triticum aestivum* | wheat | Poland | 52°50'01"N, 23°43'50"E | MG193991 |  |
| 92 | MT-3 | MT-3_ML1729_TA_POL |  | *Triticum aestivum* | wheat | Poland | 52°50'01"N, 23°43'50"E | MG193992 |  |
| 93 | MT-3 | MT-3_WS80_TA_POL |  | *Triticum aestivum* | wheat | Poland | 50°43'46"N, 22°56'52"E | MG193993 |  |
| 94 | MT-3 | MT-3_WS82_TA_POL |  | *Triticum aestivum* | wheat | Poland | 50°43'46"N, 22°56'52"E | MG193994 |  |
| 95 | MT-3 | MT-3_AT64_TS_POL |  | *Triticosecale* | triticale | Poland | 50°52'53"N, 15°54'25"E | MG193995 |  |
| 96 | MT-3 | MT-3_AS1826_TS_POL | MT-3_AS1826.D2_TS_POL | *Triticosecale* | triticale | Poland | 52°00'03"N, 16°26'33"E | MG193996 | MG076819 |
| 97 | MT-3 | MT-3_AT66_TS_POL |  | *Triticosecale* | triticale | Poland | 50°52'53"N, 15°54'25"E | MG193997 |  |
| 98 | MT-3 | MT-3_AT68_TS_POL |  | *Triticosecale* | triticale | Poland | 50°52'53"N, 15°54'25"E | MG193998 |  |
| 99 | MT-3 | MT-3_AT81_TS_POL |  | *Triticosecale* | triticale | Poland | 50°56'34"N, 15°30'13"E | MG193999 |  |
| 100 | MT-3 | MT-3_AT61_TS_POL |  | *Triticosecale* | triticale | Poland | 50°43'46"N, 15°56'43"E | MG194000 |  |
| 101 | MT-3 | MT-3_NW170_TS_POL |  | *Triticosecale* | triticale | Poland | 53°14'34"N, 17°32'19"E | MG194001 |  |
| 102 | MT-3 | MT-3_NW234_TS_POL |  | *Triticosecale* | triticale | Poland | 52°12'47"N, 17°57'11"E | MG194002 |  |
| 103 | MT-3 | MT-3_ML1660_TS_POL |  | *Triticosecale* | triticale | Poland | 51°38'28"N, 19°28'11"E | MG194003 |  |
| 104 | MT-3 | MT-3_WS298_TS_POL |  | *Triticosecale* | triticale | Poland | 51°35'35"N, 19°56'45"E | MG194004 |  |
| 105 | MT-3 | MT-3_AT446_TS_POL |  | *Triticosecale* | triticale | Poland | 50°44'34"N, 21°02'04"E | MG194005 |  |
| 106 | MT-3 | MT-3_WS304_TS_POL |  | *Triticosecale* | triticale | Poland | 51°08'40"N, 19°49'20"E | MG194006 |  |
| 107 | MT-3 | MT-3_LB113_TS_POL |  | *Triticosecale* | triticale | Poland | 51°08'49"N, 19°14'09"E | MG194007 |  |
| 108 | MT-3 | MT-3_ML1670_TS_POL |  | *Triticosecale* | triticale | Poland | 51°32'00"N, 18°36'12"E | MG194008 |  |
| 109 | MT-3 | MT-3_NW273_TS_POL |  | *Triticosecale* | triticale | Poland | 52°44'43"N, 18°10'39"E | MG194009 |  |
| 110 | MT-3 | MT-3_NW285_TS_POL |  | *Triticosecale* | triticale | Poland | 53°52'16"N, 18°30'20"E | MG194010 |  |
| 111 | MT-3 | MT-3_WS401_TS_POL |  | *Triticosecale* | triticale | Poland | 54°10'06"N, 18°39'19"E | MG194011 |  |
| 112 | MT-3 | MT-3_ML329_TS_POL |  | *Triticosecale* | triticale | Poland | 54°07'31"N, 22°51'01"E | MG194012 |  |
| 113 | MT-3 | MT-3_ML790_TS_POL |  | *Triticosecale* | triticale | Poland | 51°28'34"N, 22°03'40"E | MG194013 |  |
| 114 | MT-3 | MT-3_ML1084_TS_POL |  | *Triticosecale* | triticale | Poland | 51°54'26"N, 22°24'37"E | MG194014 |  |
| 115 | MT-3 | MT-3_AT529_TS_POL |  | *Triticosecale* | triticale | Poland | 50°21'44"N, 19°44'18"E | MG194015 |  |
| 116 | MT-3 | MT-3_ML176_TS_POL |  | *Triticosecale* | triticale | Poland | 52°40'30"N, 19°33'17"E | MG194016 |  |
| 117 | MT-3 | MT-3_ML390_TS_POL |  | *Triticosecale* | triticale | Poland | 53°50'30"N, 21°16'23"E | MG194017 |  |
| 118 | MT-3 | MT-3_ML397_TS_POL |  | *Triticosecale* | triticale | Poland | 53°50'30"N, 21°16'23"E | MG194018 |  |
| 119 | MT-3 | MT-3_ML793_TS_POL |  | *Triticosecale* | triticale | Poland | 51°25'56"N, 21°38'27"E | MG194019 |  |
| 120 | MT-3 | MT-3_ML794_TS_POL |  | *Triticosecale* | triticale | Poland | 51°25'56"N, 21°38'27"E | MG194020 |  |
| 121 | MT-3 | MT-3_EP2_TS_POL |  | *Triticosecale* | triticale | Poland | 52°50'56"N, 20°18'52"E | MG194021 |  |
| 122 | MT-3 | MT-3_ML868_TS_POL |  | *Triticosecale* | triticale | Poland | 53°45'05"N, 20°45'08"E | MG194022 |  |
| 123 | MT-3 | MT-3_ML869_TS_POL |  | *Triticosecale* | triticale | Poland | 53°45'05"N, 20°45'08"E | MG194023 |  |
| 124 | MT-3 | MT-3_ML917_TS_POL |  | *Triticosecale* | triticale | Poland | 53°43'16"N, 20°18'41"E | MG194024 |  |
| 125 | MT-3 | MT-3_NW11_TS_POL |  | *Triticosecale* | triticale | Poland | 52°01'32"N, 16°45'43"E | MG194025 |  |
| 126 | MT-3 | MT-3_quaWCM03_ER_POL | MT-3_quaWCM03.D2_ER_POL | *Elymus repens* | quackgrass | Poland | 52°27'55"N, 16°55'55"E | JQ248915* | MG076820 |
| 127 | MT-3 | MT-3_AK17_ER_POL |  | *Elymus repens* | quackgrass | Poland | 52°28'57"N, 16°40'48"E | MG194026 |  |
| 128 | MT-3 | MT-3_ML1636_ER_POL |  | *Elymus repens* | quackgrass | Poland | 52°59'14"N, 14°45'35"E | MG194027 |  |
| 129 | MT-3 | MT-3_AK333_ER_POL |  | *Elymus repens* | quackgrass | Poland | 53°24'41"N, 18°18'01"E | MG194028 |  |
| 130 | MT-3 | MT-3_AT219_ER_POL |  | *Elymus repens* | quackgrass | Poland | 51°56'09"N, 14°48'05"E | MG194029 |  |
| 131 | MT-3 | MT-3_AT441_ER_POL |  | *Elymus repens* | quackgrass | Poland | 50°45'58"N, 22°21'18"E | MG194030 |  |
| 132 | MT-3 | MT-3_AT520_ER_POL |  | *Elymus repens* | quackgrass | Poland | 50°26'12"N, 18°53'40"E | MG194031 |  |
| 133 | MT-3 | MT-3_EP112_ER_POL |  | *Elymus repens* | quackgrass | Poland | 51°21'60"N, 22°26'31"E | MG194032 |  |
| 134 | MT-3 | MT-3_LB328_ER_POL |  | *Elymus repens* | quackgrass | Poland | 51°01'16"N, 17°32'17"E | MG194033 |  |
| 135 | MT-3 | MT-3_LB73_ER_POL |  | *Elymus repens* | quackgrass | Poland | 51°37'53"N, 20°34'57"E | MG194034 |  |
| 136 | MT-3 | MT-3_LB91_ER_POL |  | *Elymus repens* | quackgrass | Poland | 50°23'45"N, 21°18'04"E | MG194035 |  |
| 137 | MT-3 | MT-3_WS340_ER_POL |  | *Elymus repens* | quackgrass | Poland | 51°15'33"N, 20°48'49"E | MG194036 |  |
| 138 | MT-3 | MT-3_ML72_ER_POL |  | *Elymus repens* | quackgrass | Poland | 52°34'53"N, 21°19'31"E | MG194037 |  |
| 139 | MT-3 | MT-3_ML241_ER_POL |  | *Elymus repens* | quackgrass | Poland | 53°42'51"N, 21°45'59"E | MG194038 |  |
| 140 | MT-3 | MT-3_ML1350_ER_POL |  | *Elymus repens* | quackgrass | Poland | 52°13'54"N, 19°57'34"E | MG194039 |  |
| 141 | MT-3 | MT-3_ML1637_ER_POL | MT-3_ML1719.D2_ER_POL | *Elymus repens* | quackgrass | Poland | 52°59'14"N, 14°45'35"E | MG194040 | MG076821 |
| 142 | MT-3 | MT-3_ML1640_ER_POL | MT-3_ML1646.D2_ER_POL | *Elymus repens* | quackgrass | Poland | 49°53'46"N, 19°22'59"E | MG194041 | MG076822 |
| 143 | MT-3 | MT-3_AK16_ER_POL |  | *Elymus repens* | quackgrass | Poland | 52°28'57"N, 16°40'48"E | MG194042 |  |
| 144 | MT-3 | MT-3_AK10_ER_POL |  | *Elymus repens* | quackgrass | Poland | 52°28'57"N, 16°40'48"E | MG194043 |  |
| 145 | MT-3 | MT-3_AT23_ER_POL |  | *Elymus repens* | quackgrass | Poland | 50°44'10"N, 16°36'41"E | MG194044 |  |
| 146 | MT-3 | MT-3_AT06_ER_POL |  | *Elymus repens* | quackgrass | Poland | 50°07'34"N, 16°42'43"E | KF975391* |  |
| 147 | MT-3 | MT-3_AS103_ER_POL |  | *Elymus repens* | quackgrass | Poland | 52°33'24"N, 17°06'54"E | MG194045 |  |
| 148 | MT-3 | MT-3_AS285_ER_POL |  | *Elymus repens* | quackgrass | Poland | 52°13'03"N, 17°13'09"E | MG194046 |  |
| 149 | MT-3 | MT-3_AS249_ER_POL |  | *Elymus repens* | quackgrass | Poland | 54°14'55"N, 19°02'14"E | MG194047 |  |
| 150 | MT-3 | MT-3_AS822_ER_POL | MT-3_AS822.D2_ER_POL | *Elymus repens* | quackgrass | Poland | 52°26'46"N, 16°46'02"E | MG194048 | MG076823 |
| 151 | MT-3 | MT-3_AS917_ER_POL |  | *Elymus repens* | quackgrass | Poland | 52°27'27"N, 17°13'18"E | MG194049 |  |
| 152 | MT-3 | MT-3_AS923_ER_POL |  | *Elymus repens* | quackgrass | Poland | 52°26'37"N, 16°45'24"E | MG194050 |  |
| 153 | MT-3 | MT-3_AS1228_ER_POL | MT-3_AS1228.D2_ER_POL | *Elymus repens* | quackgrass | Poland | 52°26'02"N, 16°45'08"E | MG194051 | MG076824 |
| 154 | MT-3 | MT-3_AS1229_ER_POL | MT-3_AS1229.D2_ER_POL | *Elymus repens* | quackgrass | Poland | 52°26'12"N, 16°45'22"E | MG194052 | MG076825 |
| 155 | MT-3 | MT-3_AS1539_ER_POL | MT-3_AS1539.D2_ER_POL | *Elymus repens* | quackgrass | Poland | 52°26'52"N, 16°45'15"E | MG194053 | MG076826 |
| 156 | MT-3 | MT-3_AS1540_ER_POL | MT-3_AS1540.D2_ER_POL | *Elymus repens* | quackgrass | Poland | 52°26'10"N, 16°45'19"E | MG194054 | MG076827 |
| 157 | MT-3 | MT-3_AS1726_ER_POL | MT-3_AS1726.D2_ER_POL | *Elymus repens* | quackgrass | Poland | 52°26'17"N, 17°14'26"E | MG194055 | MG076828 |
| 158 | MT-3 | MT-3_AS1751_ER_POL | MT-3_AS1751.D2_ER_POL | *Elymus repens* | quackgrass | Poland | 52°13'03"N, 17°13'09"E | MG194056 | MG076829 |
| 159 | MT-3 | MT-3_AT493_ER_POL |  | *Elymus repens* | quackgrass | Poland | 50°19'40"N, 20°30'39"E | MG194057 |  |
| 160 | MT-3 | MT-3_AT76_ER_POL |  | *Elymus repens* | quackgrass | Poland | 50°56'34"N, 15°30'13"E | KF975394* |  |
| 161 | MT-3 | MT-3_AT72_ER_POL |  | *Elymus repens* | quackgrass | Poland | 50°52'53"N, 15°54'25"E | MG194058 |  |
| 162 | MT-3 | MT-3_AT40_ER_POL |  | *Elymus repens* | quackgrass | Poland | 50°43'02"N, 16°34'11"E | MG194059 |  |
| 163 | MT-3 | MT-3_AT50_ER_POL |  | *Elymus repens* | quackgrass | Poland | 50°32'02"N, 16°27'43"E | KF975392* |  |
| 164 | MT-3 | MT-3_AT92_ER_POL |  | *Elymus repens* | quackgrass | Poland | 51°11'02"N, 15°13'56"E | KF975393* |  |
| 165 | MT-3 | MT-3_AT120_ER_POL |  | *Elymus repens* | quackgrass | Poland | 52°24'02"N, 16°37'31"E | MG194060 |  |
| 166 | MT-3 | MT-3_AT133_ER_POL |  | *Elymus repens* | quackgrass | Poland | 52°24'02"N, 16°37'31"E | MG194061 |  |
| 167 | MT-3 | MT-3_AT140_ER_POL |  | *Elymus repens* | quackgrass | Poland | 52°20'27"N, 17°20'00"E | MG194062 |  |
| 168 | MT-3 | MT-3_AT146_ER_POL |  | *Elymus repens* | quackgrass | Poland | 52°20'27"N, 17°20'00"E | MG194063 |  |
| 169 | MT-3 | MT-3_AS327_ER_POL | MT-3_AS327.D2_ER_POL | *Elymus repens* | quackgrass | Poland | 52°28'01"N, 16°55'27"E | JF920088* | JF920105* |
| 170 | MT-3 | MT-3_AS351_ER_POL | MT-3_AS351.D2_ER_POL | *Elymus repens* | quackgrass | Poland | 52°27'54"N, 16°55'55"E | JF920089* | JF920104* |
| 171 | MT-3 | MT-3_AT168_ER_POL |  | *Elymus repens* | quackgrass | Poland | 53°02'35"N, 15°39'53"E | MG194064 |  |
| 172 | MT-3 | MT-3_AT169_ER_POL |  | *Elymus repens* | quackgrass | Poland | 53°12'05"N, 16°38'28"E | MG194065 |  |
| 173 | MT-3 | MT-3_AT173_ER_POL |  | *Elymus repens* | quackgrass | Poland | 52°52'10"N, 16°41'20"E | MG194066 |  |
| 174 | MT-3 | MT-3_MG17_ER_POL |  | *Elymus repens* | quackgrass | Poland | 52°38'25"N, 16°28'46"E | MG194067 |  |
| 175 | MT-3 | MT-3_MG41_ER_POL |  | *Elymus repens* | quackgrass | Poland | 52°18'31"N, 15°10'38"E | MG194068 |  |
| 176 | MT-3 | MT-3_AK83_ER_POL |  | *Elymus repens* | quackgrass | Poland | 52°29'42"N, 15°22'56"E | MG194069 |  |
| 177 | MT-3 | MT-3_AK783_ER_POL |  | *Elymus repens* | quackgrass | Poland | 52°29'42"N, 15°22'56"E | MG194070 |  |
| 178 | MT-3 | MT-3_AK39_ER_POL |  | *Elymus repens* | quackgrass | Poland | 52°18'51"N, 16°21'57"E | MG194071 |  |
| 179 | MT-3 | MT-3_MG31_ER_POL |  | *Elymus repens* | quackgrass | Poland | 52°18'19"N, 15°39'43"E | MG194072 |  |
| 180 | MT-3 | MT-3_AT187_ER_POL |  | *Elymus repens* | quackgrass | Poland | 49°51'07"N, 18°57'55"E | MG194073 |  |
| 181 | MT-3 | MT-3_MG65_ER_POL |  | *Elymus repens* | quackgrass | Poland | 49°54'41"N, 19°53'55"E | MG194074 |  |
| 182 | MT-3 | MT-3_PK24_ER_POL |  | *Elymus repens* | quackgrass | Poland | 52°18'19"N, 15°39'43"E | MG194075 |  |
| 183 | MT-3 | MT-3_MG85_ER_POL |  | *Elymus repens* | quackgrass | Poland | 51°26'29"N, 16°21'32"E | MG194076 |  |
| 184 | MT-3 | MT-3_ML1718_ER_POL |  | *Elymus repens* | quackgrass | Poland | 51°18'10"N, 16°36'39"E | MG194077 |  |
| 185 | MT-3 | MT-3_NW24_ER_POL |  | *Elymus repens* | quackgrass | Poland | 51°15'42"N, 16°22'09"E | MG194078 |  |
| 186 | MT-3 | MT-3_PK17_ER_POL |  | *Elymus repens* | quackgrass | Poland | 51°16'23"N, 15°45'06"E | MG194079 |  |
| 187 | MT-3 | MT-3_MG95_ER_POL |  | *Elymus repens* | quackgrass | Poland | 51°35'34"N, 15°35'56"E | MG194080 |  |
| 188 | MT-3 | MT-3_MG88_ER_POL |  | *Elymus repens* | quackgrass | Poland | 51°33'15"N, 15°49'58"E | MG194081 |  |
| 189 | MT-3 | MT-3_ML1761_ER_POL |  | *Elymus repens* | quackgrass | Poland | 51°33'15"N, 15°49'58"E | MG194082 |  |
| 190 | MT-3 | MT-3_AK247_ER_POL |  | *Elymus repens* | quackgrass | Poland | 51°53'26"N, 15°19'45"E | MG194083 |  |
| 191 | MT-3 | MT-3_ML1720_ER_POL |  | *Elymus repens* | quackgrass | Poland | 51°56'09"N, 14°48'05"E | MG194084 |  |
| 192 | MT-3 | MT-3_MG116_ER_POL |  | *Elymus repens* | quackgrass | Poland | 51°49'34"N, 14°45'13"E | MG194085 |  |
| 193 | MT-3 | MT-3_PK54_ER_POL |  | *Elymus repens* | quackgrass | Poland | 51°32'50"N, 15°00'38"E | MG194086 |  |
| 194 | MT-3 | MT-3_PK43_ER_POL |  | *Elymus repens* | quackgrass | Poland | 51°43'02"N, 14°58'52"E | MG194087 |  |
| 195 | MT-3 | MT-3_NW68_ER_POL |  | *Elymus repens* | quackgrass | Poland | 51°19'50"N, 17°09'18"E | MG194088 |  |
| 196 | MT-3 | MT-3_NW81_ER_POL |  | *Elymus repens* | quackgrass | Poland | 51°49'07"N, 16°20'12"E | MG194089 |  |
| 197 | MT-3 | MT-3_PK72_ER_POL |  | *Elymus repens* | quackgrass | Poland | 53°32'53"N, 16°54'32"E | MG194090 |  |
| 198 | MT-3 | MT-3_AT236_ER_POL |  | *Elymus repens* | quackgrass | Poland | 51°51'29"N, 16°57'33"E | MG194091 |  |
| 199 | MT-3 | MT-3_NW103_ER_POL |  | *Elymus repens* | quackgrass | Poland | 52°00'03"N, 16°26'33"E | MG194092 |  |
| 200 | MT-3 | MT-3_AT339_ER_POL |  | *Elymus repens* | quackgrass | Poland | 53°28'21"N, 16°42'06"E | MG194093 |  |
| 201 | MT-3 | MT-3_AT302_ER_POL |  | *Elymus repens* | quackgrass | Poland | 53°45'47"N, 16°23'51"E | MG194094 |  |
| 202 | MT-3 | MT-3_AK387_ER_POL |  | *Elymus repens* | quackgrass | Poland | 53°47'53"N, 16°21'53"E | MG194095 |  |
| 203 | MT-3 | MT-3_NW123_ER_POL |  | *Elymus repens* | quackgrass | Poland | 53°43'20"N, 16°35'49"E | MG194096 |  |
| 204 | MT-3 | MT-3_AK934_ER_POL |  | *Elymus repens* | quackgrass | Poland | 53°47'22"N, 16°49'16"E | MG194097 |  |
| 205 | MT-3 | MT-3_ML1653_ER_POL |  | *Elymus repens* | quackgrass | Poland | 54°04'40"N, 17°25'58"E | MG194098 |  |
| 206 | MT-3 | MT-3_PK77_ER_POL |  | *Elymus repens* | quackgrass | Poland | 54°01'22"N, 17°21'31"E | MG194099 |  |
| 207 | MT-3 | MT-3_WS175_ER_POL |  | *Elymus repens* | quackgrass | Poland | 53°36'57"N, 17°12'29"E | MG194100 |  |
| 208 | MT-3 | MT-3_WS183_ER_POL |  | *Elymus repens* | quackgrass | Poland | 53°36'57"N, 17°12'29"E | MG194101 |  |
| 209 | MT-3 | MT-3_MG175_ER_POL |  | *Elymus repens* | quackgrass | Poland | 53°44'57"N, 17°47'38"E | MG194102 |  |
| 210 | MT-3 | MT-3_AK901_ER_POL |  | *Elymus repens* | quackgrass | Poland | 53°44'57"N, 17°47'38"E | MG194103 |  |
| 211 | MT-3 | MT-3_NW174_ER_POL |  | *Elymus repens* | quackgrass | Poland | 53°25'46"N, 17°22'53"E | MG194104 |  |
| 212 | MT-3 | MT-3_AK418_ER_POL |  | *Elymus repens* | quackgrass | Poland | 53°14'34"N, 17°32'19"E | MG194105 |  |
| 213 | MT-3 | MT-3_NW111_ER_POL |  | *Elymus repens* | quackgrass | Poland | 52°57'12"N, 17°24'51"E | MG194106 |  |
| 214 | MT-3 | MT-3_AK908_ER_POL |  | *Elymus repens* | quackgrass | Poland | 52°44'22"N, 16°56'52"E | MG194107 |  |
| 215 | MT-3 | MT-3_AK470_ER_POL |  | *Elymus repens* | quackgrass | Poland | 49°48'39"N, 22°23'59"E | MG194108 |  |
| 216 | MT-3 | MT-3_NW217_ER_POL |  | *Elymus repens* | quackgrass | Poland | 50°05'12"N, 22°47'48"E | MG194109 |  |
| 217 | MT-3 | MT-3_ML1721_ER_POL |  | *Elymus repens* | quackgrass | Poland | 50°45'58"N, 22°21'18"E | MG194110 |  |
| 218 | MT-3 | MT-3_WS273_ER_POL |  | *Elymus repens* | quackgrass | Poland | 50°48'00"N, 21°44'49"E | MG194111 |  |
| 219 | MT-3 | MT-3_ML1657_ER_POL |  | *Elymus repens* | quackgrass | Poland | 52°11'19"N, 18°19'11"E | MG194112 |  |
| 220 | MT-3 | MT-3_ML1785_ER_POL |  | *Elymus repens* | quackgrass | Poland | 52°08'09"N, 18°35'53"E | MG194113 |  |
| 221 | MT-3 | MT-3_AK496_ER_POL |  | *Elymus repens* | quackgrass | Poland | 52°08'09"N, 18°35'53"E | MG194114 |  |
| 222 | MT-3 | MT-3_PK93_ER_POL |  | *Elymus repens* | quackgrass | Poland | 52°30'18"N, 18°44'32"E | MG194115 |  |
| 223 | MT-3 | MT-3_AK536_ER_POL |  | *Elymus repens* | quackgrass | Poland | 51°38'28"N, 19°28'11"E | MG194116 |  |
| 224 | MT-3 | MT-3_LB99_ER_POL |  | *Elymus repens* | quackgrass | Poland | 51°35'35"N, 19°56'45"E | MG194117 |  |
| 225 | MT-3 | MT-3_NW249_ER_POL |  | *Elymus repens* | quackgrass | Poland | 51°11'45"N, 21°20'27"E | MG194118 |  |
| 226 | MT-3 | MT-3_AK538_ER_POL |  | *Elymus repens* | quackgrass | Poland | 50°24'34"N, 21°02'54"E | MG194119 |  |
| 227 | MT-3 | MT-3_LB80_ER_POL |  | *Elymus repens* | quackgrass | Poland | 50°59'17"N, 20°53'18"E | MG194120 |  |
| 228 | MT-3 | MT-3_NW237_ER_POL |  | *Elymus repens* | quackgrass | Poland | 51°19'54"N, 20°00'44"E | MG194121 |  |
| 229 | MT-3 | MT-3_LB131_ER_POL |  | *Elymus repens* | quackgrass | Poland | 51°08'40"N, 19°49'20"E | MG194122 |  |
| 230 | MT-3 | MT-3_ML1723_ER_POL |  | *Elymus repens* | quackgrass | Poland | 51°08'40"N, 19°49'20"E | MG194123 |  |
| 231 | MT-3 | MT-3_LB169_ER_POL |  | *Elymus repens* | quackgrass | Poland | 51°08'40"N, 19°49'20"E | MG194124 |  |
| 232 | MT-3 | MT-3_LB88_ER_POL |  | *Elymus repens* | quackgrass | Poland | 51°00'04"N, 19°43'12"E | MG194125 |  |
| 233 | MT-3 | MT-3_AK593_ER_POL |  | *Elymus repens* | quackgrass | Poland | 50°50'41"N, 20°08'38"E | MG194126 |  |
| 234 | MT-3 | MT-3_WS387_ER_POL |  | *Elymus repens* | quackgrass | Poland | 50°41'50"N, 20°28'48"E | MG194127 |  |
| 235 | MT-3 | MT-3_AK599_ER_POL |  | *Elymus repens* | quackgrass | Poland | 50°39'25"N, 20°01'34"E | MG194128 |  |
| 236 | MT-3 | MT-3_NW270_ER_POL |  | *Elymus repens* | quackgrass | Poland | 51°06'04"N, 19°29'34"E | MG194129 |  |
| 237 | MT-3 | MT-3_NW269_ER_POL |  | *Elymus repens* | quackgrass | Poland | 51°07'13"N, 18°55'17"E | MG194130 |  |
| 238 | MT-3 | MT-3_AT469_ER_POL |  | *Elymus repens* | quackgrass | Poland | 51°12'33"N, 18°29'30"E | MG194131 |  |
| 239 | MT-3 | MT-3_ML1669_ER_POL |  | *Elymus repens* | quackgrass | Poland | 51°12'33"N, 18°29'30"E | MG194132 |  |
| 240 | MT-3 | MT-3_ML1671_ER_POL |  | *Elymus repens* | quackgrass | Poland | 51°30'58"N, 18°57'50"E | MG194133 |  |
| 241 | MT-3 | MT-3_AT472_ER_POL |  | *Elymus repens* | quackgrass | Poland | 51°42'50"N, 18°50'53"E | MG194134 |  |
| 242 | MT-3 | MT-3_AT475_ER_POL |  | *Elymus repens* | quackgrass | Poland | 53°05'47"N, 18°14'44"E | MG194135 |  |
| 243 | MT-3 | MT-3_AT476_ER_POL |  | *Elymus repens* | quackgrass | Poland | 53°09'02"N, 18°16'28"E | MG194136 |  |
| 244 | MT-3 | MT-3_LB191_ER_POL |  | *Elymus repens* | quackgrass | Poland | 53°21'56"N, 18°09'02"E | MG194137 |  |
| 245 | MT-3 | MT-3_JK934_ER_POL |  | *Elymus repens* | quackgrass | Poland | 54°01'51"N, 18°07'19"E | MG194138 |  |
| 246 | MT-3 | MT-3_ML1725_ER_POL |  | *Elymus repens* | quackgrass | Poland | 54°36'21"N, 17°15'28"E | MG194139 |  |
| 247 | MT-3 | MT-3_ML1726_ER_POL |  | *Elymus repens* | quackgrass | Poland | 54°42'46"N, 17°58'03"E | MG194140 |  |
| 248 | MT-3 | MT-3_NW365_ER_POL |  | *Elymus repens* | quackgrass | Poland | 50°34'29"N, 17°50'55"E | MG194141 |  |
| 249 | MT-3 | MT-3_ML1688_ER_POL |  | *Elymus repens* | quackgrass | Poland | 50°35'06"N, 17°57'41"E | MG194142 |  |
| 250 | MT-3 | MT-3_LB285_ER_POL |  | *Elymus repens* | quackgrass | Poland | 50°29'26"N, 17°32'26"E | MG194143 |  |
| 251 | MT-3 | MT-3_AL134_ER_POL |  | *Elymus repens* | quackgrass | Poland | 50°07'25"N, 17°42'20"E | MG194144 |  |
| 252 | MT-3 | MT-3_LB258_ER_POL |  | *Elymus repens* | quackgrass | Poland | 50°05'35"N, 18°22'40"E | MG194145 |  |
| 253 | MT-3 | MT-3_LB268_ER_POL |  | *Elymus repens* | quackgrass | Poland | 50°05'35"N, 18°22'40"E | MG194146 |  |
| 254 | MT-3 | MT-3_AL155_ER_POL |  | *Elymus repens* | quackgrass | Poland | 50°12'09"N, 18°47'48"E | MG194147 |  |
| 255 | MT-3 | MT-3_ML1730_ER_POL |  | *Elymus repens* | quackgrass | Poland | 50°07'18"N, 19°12'10"E | MG194148 |  |
| 256 | MT-3 | MT-3_NW338_ER_POL |  | *Elymus repens* | quackgrass | Poland | 50°03'23"N, 19°44'51"E | MG194149 |  |
| 257 | MT-3 | MT-3_AT489_ER_POL |  | *Elymus repens* | quackgrass | Poland | 50°11'39"N, 20°24'30"E | MG194150 |  |
| 258 | MT-3 | MT-3_AT490_ER_POL |  | *Elymus repens* | quackgrass | Poland | 50°11'39"N, 20°24'30"E | MG194151 |  |
| 259 | MT-3 | MT-3_LB288_ER_POL |  | *Elymus repens* | quackgrass | Poland | 50°22'34"N, 20°13'51"E | MG194152 |  |
| 260 | MT-3 | MT-3_NW371_ER_POL |  | *Elymus repens* | quackgrass | Poland | 50°21'44"N, 19°44'18"E | MG194153 |  |
| 261 | MT-3 | MT-3_LB299_ER_POL |  | *Elymus repens* | quackgrass | Poland | 50°25'38"N, 19°14'47"E | MG194154 |  |
| 262 | MT-3 | MT-3_AT500_ER_POL |  | *Elymus repens* | quackgrass | Poland | 50°41'18"N, 18°46'50"E | MG194155 |  |
| 263 | MT-3 | MT-3_AT503_ER_POL |  | *Elymus repens* | quackgrass | Poland | 50°50'27"N, 18°39'07"E | MG194156 |  |
| 264 | MT-3 | MT-3_WS440_ER_POL |  | *Elymus repens* | quackgrass | Poland | 50°49'21"N, 18°32'18"E | MG194157 |  |
| 265 | MT-3 | MT-3_ML1763_ER_POL |  | *Elymus repens* | quackgrass | Poland | 50°29'56"N, 18°38'25"E | MG194158 |  |
| 266 | MT-3 | MT-3_ML1696_ER_POL |  | *Elymus repens* | quackgrass | Poland | 50°26'12"N, 18°53'40"E | MG194159 |  |
| 267 | MT-3 | MT-3_LB322_ER_POL |  | *Elymus repens* | quackgrass | Poland | 51°03'44"N, 18°02'11"E | MG194160 |  |
| 268 | MT-3 | MT-3_LB301_ER_POL |  | *Elymus repens* | quackgrass | Poland | 51°11'41"N, 17°46'34"E | MG194161 |  |
| 269 | MT-3 | MT-3_LB347_ER_POL |  | *Elymus repens* | quackgrass | Poland | 51°25'15"N, 17°53'58"E | MG194162 |  |
| 270 | MT-3 | MT-3_AK436_ER_POL |  | *Elymus repens* | quackgrass | Poland | 52°24'22"N, 17°30'27"E | MG194163 |  |
| 271 | MT-3 | MT-3_ML7_ER_POL |  | *Elymus repens* | quackgrass | Poland | 52°08'18"N, 20°18'29"E | MG194164 |  |
| 272 | MT-3 | MT-3_ML8_ER_POL |  | *Elymus repens* | quackgrass | Poland | 52°08'18"N, 20°18'29"E | MG194165 |  |
| 273 | MT-3 | MT-3_ML1312_ER_POL |  | *Elymus repens* | quackgrass | Poland | 52°30'15"N, 20°33'17"E | MG194166 |  |
| 274 | MT-3 | MT-3_ML1767_ER_POL |  | *Elymus repens* | quackgrass | Poland | 52°13'54"N, 19°57'34"E | MG194167 |  |
| 275 | MT-3 | MT-3_AK999_ER_POL |  | *Elymus repens* | quackgrass | Poland | 51°47'26"N, 19°59'37"E | MG194168 |  |
| 276 | MT-3 | MT-3_ML1714_ER_POL |  | *Elymus repens* | quackgrass | Poland | 52°39'40"N, 20°12'19"E | MG194169 |  |
| 277 | MT-3 | MT-3_ML161_ER_POL |  | *Elymus repens* | quackgrass | Poland | 52°42'35"N, 19°44'52"E | MG194170 |  |
| 278 | MT-3 | MT-3_AK247A_ER_POL |  | *Elymus repens* | quackgrass | Poland | 52°40'30"N, 19°33'17"E | MG194171 |  |
| 279 | MT-3 | MT-3_JP7_ER_POL |  | *Elymus repens* | quackgrass | Poland | 52°59'18"N, 19°47'50"E | MG194172 |  |
| 280 | MT-3 | MT-3_JP29_ER_POL |  | *Elymus repens* | quackgrass | Poland | 52°54'31"N, 19°33'28"E | MG194173 |  |
| 281 | MT-3 | MT-3_ML1769_ER_POL |  | *Elymus repens* | quackgrass | Poland | 52°54'31"N, 19°33'28"E | MG194174 |  |
| 282 | MT-3 | MT-3_ML227A_ER_POL |  | *Elymus repens* | quackgrass | Poland | 53°25'01"N, 21°22'14"E | MG194175 |  |
| 283 | MT-3 | MT-3_ML257_ER_POL |  | *Elymus repens* | quackgrass | Poland | 53°52'09"N, 21°41'07"E | MG194176 |  |
| 284 | MT-3 | MT-3_ML262_ER_POL |  | *Elymus repens* | quackgrass | Poland | 53°52'09"N, 21°41'07"E | MG194177 |  |
| 285 | MT-3 | MT-3_ML1711_ER_POL |  | *Elymus repens* | quackgrass | Poland | 53°50'37"N, 22°55'52"E | MG194178 |  |
| 286 | MT-3 | MT-3_ML1712_ER_POL |  | *Elymus repens* | quackgrass | Poland | 54°07'31"N, 22°51'01"E | MG194179 |  |
| 287 | MT-3 | MT-3_ML358_ER_POL |  | *Elymus repens* | quackgrass | Poland | 54°07'31"N, 22°51'01"E | MG194180 |  |
| 288 | MT-3 | MT-3_ML1447_ER_POL |  | *Elymus repens* | quackgrass | Poland | 53°50'30"N, 21°16'23"E | MG194181 |  |
| 289 | MT-3 | MT-3_ML1453_ER_POL |  | *Elymus repens* | quackgrass | Poland | 53°43'10"N, 21°04'09"E | MG194182 |  |
| 290 | MT-3 | MT-3_ML1454_ER_POL |  | *Elymus repens* | quackgrass | Poland | 53°43'10"N, 21°04'09"E | MG194183 |  |
| 291 | MT-3 | MT-3_JP52_ER_POL |  | *Elymus repens* | quackgrass | Poland | 53°52'22"N, 22°15'50"E | MG194184 |  |
| 292 | MT-3 | MT-3_ML1747_ER_POL |  | *Elymus repens* | quackgrass | Poland | 53°57'49"N, 22°32'44"E | MG194185 |  |
| 293 | MT-3 | MT-3_ML585_ER_POL |  | *Elymus repens* | quackgrass | Poland | 52°59'22"N, 21°36'02"E | MG194186 |  |
| 294 | MT-3 | MT-3_ML598_ER_POL |  | *Elymus repens* | quackgrass | Poland | 53°21'29"N, 21°36'29"E | MG194187 |  |
| 295 | MT-3 | MT-3_ML613_ER_POL |  | *Elymus repens* | quackgrass | Poland | 49°34'34"N, 21°45'38"E | MG194188 |  |
| 296 | MT-3 | MT-3_ML627_ER_POL |  | *Elymus repens* | quackgrass | Poland | 53°06'23"N, 21°58'53"E | MG194189 |  |
| 297 | MT-3 | MT-3_ML1516_ER_POL |  | *Elymus repens* | quackgrass | Poland | 52°50'25"N, 21°49'46"E | MG194190 |  |
| 298 | MT-3 | MT-3_ML682_ER_POL |  | *Elymus repens* | quackgrass | Poland | 52°28'05"N, 21°58'03"E | MG194191 |  |
| 299 | MT-3 | MT-3_ML687_ER_POL |  | *Elymus repens* | quackgrass | Poland | 52°21'52"N, 22°28'29"E | MG194192 |  |
| 300 | MT-3 | MT-3_ML1702_ER_POL |  | *Elymus repens* | quackgrass | Poland | 52°29'51"N, 22°49'28"E | MG194193 |  |
| 301 | MT-3 | MT-3_ML708_ER_POL |  | *Elymus repens* | quackgrass | Poland | 52°29'51"N, 22°49'28"E | MG194194 |  |
| 302 | MT-3 | MT-3_ML716_ER_POL |  | *Elymus repens* | quackgrass | Poland | 52°32'44"N, 23°15'24"E | MG194195 |  |
| 303 | MT-3 | MT-3_ML724_ER_POL |  | *Elymus repens* | quackgrass | Poland | 52°18'58"N, 23°07'54"E | MG194196 |  |
| 304 | MT-3 | MT-3_ML759_ER_POL |  | *Elymus repens* | quackgrass | Poland | 52°08'06"N, 21°58'28"E | MG194197 |  |
| 305 | MT-3 | MT-3_ML772_ER_POL |  | *Elymus repens* | quackgrass | Poland | 52°18'09"N, 20°05'35"E | MG194198 |  |
| 306 | MT-3 | MT-3_ML803_ER_POL |  | *Elymus repens* | quackgrass | Poland | 51°42'59"N, 21°23'48"E | MG194199 |  |
| 307 | MT-3 | MT-3_ML810_ER_POL |  | *Elymus repens* | quackgrass | Poland | 53°09'43"N, 20°26'38"E | MG194200 |  |
| 308 | MT-3 | MT-3_ML1752_ER_POL |  | *Elymus repens* | quackgrass | Poland | 53°23'50"N, 20°18'59"E | MG194201 |  |
| 309 | MT-3 | MT-3_ML827_ER_POL |  | *Elymus repens* | quackgrass | Poland | 53°23'50"N, 20°18'59"E | MG194202 |  |
| 310 | MT-3 | MT-3_ML1755_ER_POL |  | *Elymus repens* | quackgrass | Poland | 53°19'52"N, 20°35'05"E | MG194203 |  |
| 311 | MT-3 | MT-3_EP37_ER_POL |  | *Elymus repens* | quackgrass | Poland | 53°38'11"N, 19°40'24"E | MG194204 |  |
| 312 | MT-3 | MT-3_ML882_ER_POL |  | *Elymus repens* | quackgrass | Poland | 53°45'05"N, 20°45'08"E | MG194205 |  |
| 313 | MT-3 | MT-3_ML884_ER_POL |  | *Elymus repens* | quackgrass | Poland | 53°50'36"N, 20°36'14"E | MG194206 |  |
| 314 | MT-3 | MT-3_ML902_ER_POL |  | *Elymus repens* | quackgrass | Poland | 53°50'17"N, 20°18'54"E | MG194207 |  |
| 315 | MT-3 | MT-3_ML1554_ER_POL |  | *Elymus repens* | quackgrass | Poland | 53°50'17"N, 20°18'54"E | MG194208 |  |
| 316 | MT-3 | MT-3_ML923_ER_POL |  | *Elymus repens* | quackgrass | Poland | 53°54'19"N, 19°49'47"E | MG194209 |  |
| 317 | MT-3 | MT-3_ML945_ER_POL |  | *Elymus repens* | quackgrass | Poland | 54°08'59"N, 19°30'47"E | MG194210 |  |
| 318 | MT-3 | MT-3_ML972_ER_POL |  | *Elymus repens* | quackgrass | Poland | 54°13'54"N, 20°27'56"E | MG194211 |  |
| 319 | MT-3 | MT-3_ML986_ER_POL |  | *Elymus repens* | quackgrass | Poland | 54°18'42"N, 20°32'43"E | MG194212 |  |
| 320 | MT-3 | MT-3_ML1555_ER_POL |  | *Elymus repens* | quackgrass | Poland | 54°18'42"N, 20°32'43"E | MG194213 |  |
| 321 | MT-3 | MT-3_EP42_ER_POL |  | *Elymus repens* | quackgrass | Poland | 52°06'03"N, 23°21'37"E | MG194214 |  |
| 322 | MT-3 | MT-3_ML1610_ER_POL |  | *Elymus repens* | quackgrass | Poland | 52°02'50"N, 23°35'19"E | MG194215 |  |
| 323 | MT-3 | MT-3_ML1556_ER_POL |  | *Elymus repens* | quackgrass | Poland | 51°22'31"N, 23°30'24"E | MG194216 |  |
| 324 | MT-3 | MT-3_EP102_ER_POL |  | *Elymus repens* | quackgrass | Poland | 51°05'40"N, 23°19'19"E | MG194217 |  |
| 325 | MT-3 | MT-3_ML1034_ER_POL |  | *Elymus repens* | quackgrass | Poland | 51°04'59"N, 22°35'32"E | MG194218 |  |
| 326 | MT-3 | MT-3_ML1738_ER_POL |  | *Elymus repens* | quackgrass | Poland | 51°04'59"N, 22°35'32"E | MG194219 |  |
| 327 | MT-3 | MT-3_ML1561_ER_POL |  | *Elymus repens* | quackgrass | Poland | 51°27'30"N, 22°40'45"E | MG194220 |  |
| 328 | MT-3 | MT-3_ML1075_ER_POL |  | *Elymus repens* | quackgrass | Poland | 51°44'31"N, 22°49'05"E | MG194221 |  |
| 329 | MT-3 | MT-3_ML1564_ER_POL |  | *Elymus repens* | quackgrass | Poland | 53°26'13"N, 22°59'01"E | MG194222 |  |
| 330 | MT-3 | MT-3_ML1178_ER_POL |  | *Elymus repens* | quackgrass | Poland | 53°31'09"N, 22°56'46"E | MG194223 |  |
| 331 | MT-3 | MT-3_ML1567_ER_POL |  | *Elymus repens* | quackgrass | Poland | 53°31'09"N, 22°56'46"E | MG194224 |  |
| 332 | MT-3 | MT-3_ML1566_ER_POL |  | *Elymus repens* | quackgrass | Poland | 53°45'16"N, 23°25'25"E | MG194225 |  |
| 333 | MT-3 | MT-3_ML1191_ER_POL |  | *Elymus repens* | quackgrass | Poland | 53°18'32"N, 23°32'34"E | MG194226 |  |
| 334 | MT-3 | MT-3_ML1568_ER_POL |  | *Elymus repens* | quackgrass | Poland | 53°18'32"N, 23°32'34"E | MG194227 |  |
| 335 | MT-3 | MT-3_ML1198_ER_POL |  | *Elymus repens* | quackgrass | Poland | 53°18'40"N, 23°43'24"E | MG194228 |  |
| 336 | MT-3 | MT-3_ML1727_ER_POL |  | *Elymus repens* | quackgrass | Poland | 53°18'40"N, 23°43'24"E | MG194229 |  |
| 337 | MT-3 | MT-3_ML1722_ER_POL |  | *Elymus repens* | quackgrass | Poland | 52°59'31"N, 23°21'37"E | MG194230 |  |
| 338 | MT-3 | MT-3_ML1728_ER_POL |  | *Elymus repens* | quackgrass | Poland | 52°58'20"N, 23°51'54"E | MG194231 |  |
| 339 | MT-3 | MT-3_ML1254_ER_POL |  | *Elymus repens* | quackgrass | Poland | 52°50'01"N, 23°43'50"E | MG194232 |  |
| 340 | MT-3 | MT-3_ML1275_ER_POL |  | *Elymus repens* | quackgrass | Poland | 52°46'24"N, 23°25'38"E | MG194233 |  |
| 341 | MT-3 | MT-3_ML1595_ER_POL |  | *Elymus repens* | quackgrass | Poland | 52°50'56"N, 23°04'42"E | MG194234 |  |
| 342 | MT-3 | MT-3_ML1286_ER_POL |  | *Elymus repens* | quackgrass | Poland | 52°50'56"N, 23°04'42"E | MG194235 |  |
| 343 | MT-3 | MT-3_WS03_ER_POL |  | *Elymus repens* | quackgrass | Poland | 52°08'19"N, 17°03'48"E | MG194236 |  |
| 344 | MT-3 | MT-3_WS65_ER_POL |  | *Elymus repens* | quackgrass | Poland | 51°42'31"N, 17°52'41"E | MG194237 |  |
| 345 | MT-3 | MT-3_AK183_ER_POL |  | *Elymus repens* | quackgrass | Poland | 53°51'37"N, 14°45'47"E | MG194238 |  |
| 346 | MT-3 | MT-3_AK845_ER_POL |  | *Elymus repens* | quackgrass | Poland | 52°01'32"N, 16°45'43"E | MG194239 |  |
| 347 | MT-3 | MT-3_WS140_ER_POL |  | *Elymus repens* | quackgrass | Poland | 50°56'31"N, 23°18'28"E | MG194240 |  |
| 348 | MT-3 | MT-3_WS150_ER_POL |  | *Elymus repens* | quackgrass | Poland | 50°56'31"N, 23°18'28"E | MG194241 |  |
| 349 | MT-3 | MT-3_NW142_ER_POL |  | *Elymus repens* | quackgrass | Poland | 50°28'15"N, 23°37'02"E | MG194242 |  |
| 350 | MT-3 | MT-3_ML1733_ER_POL |  | *Elymus repens* | quackgrass | Poland | 50°28'15"N, 23°37'02"E | MG194243 |  |
| 351 | MT-3 | MT-3_AT329_ER_POL |  | *Elymus repens* | quackgrass | Poland | 50°17'58"N, 23°09'04"E | MG194244 |  |
| 352 | MT-3 | MT-3_NW133_ER_POL |  | *Elymus repens* | quackgrass | Poland | 50°50'49"N, 22°39'33"E | MG194245 |  |
| 353 | MT-3 | MT-3_NW177_ER_POL |  | *Elymus repens* | quackgrass | Poland | 53°17'44"N, 15°22'30"E | MG194246 |  |
| 354 | MT-3 | MT-3_AK874_ER_POL |  | *Elymus repens* | quackgrass | Poland | 53°27'49"N, 15°35'12"E | MG194247 |  |
| 355 | MT-3 | MT-3_MG206_ER_POL |  | *Elymus repens* | quackgrass | Poland | 53°35'09"N, 15°27'21"E | MG194248 |  |
| 356 | MT-3 | MT-3_AT399_ER_POL |  | *Elymus repens* | quackgrass | Poland | 53°26'04"N, 14°56'28"E | MG194249 |  |
| 357 | MT-3 | MT-3_AT378_ER_POL |  | *Elymus repens* | quackgrass | Poland | 53°40'18"N, 15°02'23"E | MG194250 |  |
| 358 | MT-3 | MT-3_WS220_ER_POL |  | *Elymus repens* | quackgrass | Poland | 52°59'14"N, 14°45'35"E | MG194251 |  |
| 359 | MT-3 | MT-3_WS226_ER_POL |  | *Elymus repens* | quackgrass | Poland | 52°59'14"N, 14°45'35"E | MG194252 |  |
| 360 | MT-3 | MT-3_NW205_ER_POL |  | *Elymus repens* | quackgrass | Poland | 52°44'38"N, 15°00'21"E | MG194253 |  |
| 361 | MT-3 | MT-3_AT351_ER_POL |  | *Elymus repens* | quackgrass | Poland | 52°50'30"N, 14°28'36"E | MG194254 |  |
| 362 | MT-3 | MT-3_NW211_ER_POL |  | *Elymus repens* | quackgrass | Poland | 53°08'42"N, 15°09'47"E | MG194255 |  |
| 363 | MT-3 | MT-3_AT15_AE_POL | MT-3_AT15.D2_AE_POL | *Arrhenatherum elatius* | tall oat-grass | Poland | 50°29'20"N, 16°51'49"E | MG194256 | MG076831 |
| 364 | MT-3 | MT-3_AS1873_EC_POL | MT-3_AS1873.D2_EC_POL | *Echinochloa crus-galli* | cockspur | Poland | 52°02'42"N, 16°45'55"E | MG194257 | MG076830 |
| 365 | MT-3 | MT-3_broWCM3_BI_POL | MT-3_broWCM3.D2_BI_POL | *Bromus inermis* | smooth brome | Poland | 52°28'03"N, 16°54'31"E | JQ248921* | JQ918884* |
| 366 | MT-3 | MT-3_AS25_BI_POL |  | *Bromus inermis* | smooth brome | Poland | 52°22'60"N, 16°54'60"E | FJ387564* |  |
| 367 | MT-3 | MT-3_ML207_BI_POL |  | *Bromus inermis* | smooth brome | Poland | 52°59'18"N, 19°47'50"E | MG194258 |  |
| 368 | MT-3 | MT-3_ML925_BI_POL |  | *Bromus inermis* | smooth brome | Poland | 54°01'27"N, 19°27'57"E | MG194259 |  |
| 369 | MT-3 | MT-3_ML011_BI_POL |  | *Bromus inermis* | smooth brome | Poland | 51°09'24"N, 23°48'00"E | MG194260 |  |
| 370 | MT-3 | MT-3_MZ254_AS_POL |  | *Avena sativa* | oat | Poland | 53°07'03"N, 22°50'58"E | MG194261 |  |
| 371 | MT-3 | MT-3_ML228A_HV_POL |  | *Hordeum vulgare* | barley | Poland | 53°42'51"N, 21°45'59"E | MG194262 |  |
| 372 | MT-3 | MT-3_ML379_HV_POL |  | *Hordeum vulgare* | barley | Poland | 53°50'30"N, 21°16'23"E | MG194263 |  |
| 373 | MT-4 | MT-4_AK5_TA_POL |  | *Triticum aestivum* | wheat | Poland | 52°28'57"N, 16°40'48"E | MG208687 |  |
| 374 | MT-4 | MT-4_AS1154_TA_POL |  | *Triticum aestivum* | wheat | Poland | 51°51'41"N, 17°10'08"E | MG208688 |  |
| 375 | MT-4 | MT-4_AT56_TA_POL |  | *Triticum aestivum* | wheat | Poland | 50°32'02"N, 16°27'43"E | MG208689 |  |
| 376 | MT-4 | MT-4_AS313_TA_POL |  | *Triticum aestivum* | wheat | Poland | 52°02'24"N, 16°46'09"E | MG208690 |  |
| 377 | MT-4 | MT-4_AT04_TA_POL |  | *Triticum aestivum* | wheat | Poland | 50°07'34"N, 16°42'43"E | MG208691 |  |
| 378 | MT-4 | MT-4_NW15_SR_POL | MT-4_NW15.D2_SR_POL | *Secale cereale* | rye | Poland | 51°56'14"N, 16°01'27"E | MG208692 | MG193928 |
| 379 | MT-4 | MT-4_AS1115_HM_POL | MT-4_AS1115.D2_HM_POL | *Hordeum murinum* | wall barley | Poland | 52°27'47"N, 16°55'36"E | MG208693 | MG076832 |
| 380 | MT-4 | MT-4_AS909_ZM_POL | MT-4_AS909.D2_ZM_POL | *Zea mays* | maize | Poland | 52°26'02"N, 16°45'08"E | MG208694 | MG076833 |
| 381 | MT-4 | MT-4_AT94_ER_POL |  | *Elymus repens* | quackgrass | Poland | 51°11'02"N, 15°13'55"E | MG208695 |  |
| 382 | MT-4 | MT-4_AT29A_ER_POL |  | *Elymus repens* | quackgrass | Poland | 50°44'10"N, 16°36'41"E | MG208696 |  |
| 383 | MT-4 | MT-4_AT36_ER_POL |  | *Elymus repens* | quackgrass | Poland | 50°43'02"N, 16°34'11"E | MG208697 |  |
| 384 | MT-4 | MT-4_AT37_ER_POL |  | *Elymus repens* | quackgrass | Poland | 50°43'02"N, 16°34'11"E | MG208698 |  |
| 385 | MT-4 | MT-4_AT41_ER_POL |  | *Elymus repens* | quackgrass | Poland | 50°43'02"N, 16°34'11"E | MG208699 |  |
| 386 | MT-4 | MT-4_AT87_ER_POL |  | *Elymus repens* | quackgrass | Poland | 51°11'02"N, 15°13'55"E | MG208700 |  |
| 387 | MT-4 | MT-4_AK98_ER_POL |  | *Elymus repens* | quackgrass | Poland | 52°29'42"N, 15°22'56"E | MG208701 |  |
| 388 | MT-4 | MT-4_AK755_ER_POL |  | *Elymus repens* | quackgrass | Poland | 50°55'59"N, 16°34'22"E | MG208702 |  |
| 389 | MT-4 | MT-4_AS236_ER_POL |  | *Elymus repens* | quackgrass | Poland | 53°12'29"N, 18°59'47"E | MG208703 |  |
| 390 | MT-4 | MT-4_AS835_ER_POL | MT-4_AS835.D2_ER_POL | *Elymus repens* | quackgrass | Poland | 52°12'33"N, 17°26'37"E | MG208704 | MG076834 |
| 391 | MT-4 | MT-4_AS1110_ER_POL | MT-4_AS1110.D2_ER_POL | *Elymus repens* | quackgrass | Poland | 52°27'53"N, 16°55'54"E | MG208705 | MG076835 |
| 392 | MT-4 | MT-4_AS1174_ER_POL |  | *Elymus repens* | quackgrass | Poland | 50°38'47"N, 23°09'13"E | MG208706 |  |
| 393 | MT-4 | MT-4_AT70_ER_POL |  | *Elymus repens* | quackgrass | Poland | 50°52'53"N, 15°54'25"E | MG208707 |  |
| 394 | MT-4 | MT-4_AS292_ER_POL | MT-4_AS292.D2_ER_POL | *Elymus repens* | quackgrass | Poland | 52°28'41"N, 16°56'04"E | JF920090* | JF920106* |
| 395 | MT-4 | MT-4_quaWCM5_ER_POL | MT-4_quaWCM5.D2_ER_POL | *Elymus repens* | quackgrass | Poland | 52°27'55"N, 16°55'55"E | JQ248916* | JF920105* |
| 396 | MT-4 | MT-4_quaWCM6_ER_POL | MT-4_quaWCM6.D2_ER_POL | *Elymus repens* | quackgrass | Poland | 52°27'55"N, 16°55'55"E | JQ248917* | JF920106* |
| 397 | MT-4 | MT-4_quaWCM7_ER_POL | MT-4_quaWCM7.D2_ER_POL | *Elymus repens* | quackgrass | Poland | 52°27'55"N, 16°55'55"E | JQ248918* | JQ918881* |
| 398 | MT-4 | MT-4_AS827_EC_POL | MT-4_AS827.D2_EC_POL | *Echinochloa crus-galli* | cockspur | Poland | 52°12'33"N, 17°26'37"E | MG208708 | MG076836 |
| 399 | MT-4 | MT-4_broWCM4_BI_POL | MT-4_broWCM4.D2_BI_POL | *Bromus inermis* | smooth brome | Poland | 52°28'03"N, 16°54'31"E | JQ248922* | JQ918885* |
| 400 | MT-4 | MT-4_AS459_BI_POL |  | *Bromus inermis* | smooth brome | Poland | 52°22'60"N, 16°54'60"E | MG208709 |  |
| 401 | MT-4 | MT-4_AT29_BI_POL |  | *Bromus inermis* | smooth brome | Poland | 50°44'10"N, 16°36'41"E | KF975396* |  |
| 402 | MT-4 | MT-4_AT16_AE_POL |  | *Arrhenatherum elatius* | tall oat-grass | Poland | 50°29'20"N, 16°51'49"E | MG208710 |  |
| 403 | MT-4 | MT-4_AS483_TA_FRA | MT-4_AS483.D2_TA_FRA | *Triticum aestivum* | wheat | France | 47°54'20"N, 01°33'11"E | JF920081* | JF920101* |
| 404 | MT-5 | MT-5_AT129_AE_POL |  | *Arrhenatherum elatius* | tall oat-grass | Poland | 52°28'57"N, 16°40'48"E | KF975395* |  |
| 405 | MT-5 | MT-5_AT127_AE_POL |  | *Arrhenatherum elatius* | tall oat-grass | Poland | 52°28'57"N, 16°40'48"E | MG208711 |  |
| 406 | MT-5 | MT-5_ML1650_AE_POL |  | *Arrhenatherum elatius* | tall oat-grass | Poland | 50°55'59"N, 16°34'22"E | MG208712 |  |
| 407 | MT-5 | MT-5_ML325_AE_POL |  | *Arrhenatherum elatius* | tall oat-grass | Poland | 53°50'37"N, 22°55'52"E | MG208713 |  |
| 408 | MT-5 | MT-5_AK511_AE_POL |  | *Arrhenatherum elatius* | tall oat-grass | Poland | 52°35'03"N, 18°17'02"E | MG208714 |  |
| 409 | MT-5 | MT-5_AT444_AE_POL |  | *Arrhenatherum elatius* | tall oat-grass | Poland | 50°15'35"N, 21°46'18"E | MG208715 |  |
| 410 | MT-5 | MT-5_AK20_AE_POL |  | *Arrhenatherum elatius* | tall oat-grass | Poland | 52°47'26"N, 15°38'23"E | MG208716 |  |
| 411 | MT-5 | MT-5_AK221_AE_POL |  | *Arrhenatherum elatius* | tall oat-grass | Poland | 51°15'42"N, 16°22'09"E | MG208717 |  |
| 412 | MT-5 | MT-5_AK230_AE_POL |  | *Arrhenatherum elatius* | tall oat-grass | Poland | 51°42'35"N, 15°18'02"E | MG208718 |  |
| 413 | MT-5 | MT-5_AK372_AE_POL |  | *Arrhenatherum elatius* | tall oat-grass | Poland | 53°45'47"N, 16°23'51"E | MG208719 |  |
| 414 | MT-5 | MT-5_AT198_AE_POL |  | *Arrhenatherum elatius* | tall oat-grass | Poland | 49°33'35"N, 19°58'31"E | MG208720 |  |
| 415 | MT-5 | MT-5_AT386_AE_POL |  | *Arrhenatherum elatius* | tall oat-grass | Poland | 53°26'04"N, 14°56'28"E | MG208721 |  |
| 416 | MT-5 | MT-5_ML477A_AE_POL |  | *Arrhenatherum elatius* | tall oat-grass | Poland | 53°42'57"N, 22°24'12"E | MG208722 |  |
| 417 | MT-5 | MT-5_AT454_AE_POL |  | *Arrhenatherum elatius* | tall oat-grass | Poland | 51°37'53"N, 20°34'57"E | MG208723 |  |
| 418 | MT-5 | MT-5_ML1661_AE_POL |  | *Arrhenatherum elatius* | tall oat-grass | Poland | 51°37'53"N, 20°34'57"E | MG208724 |  |
| 419 | MT-5 | MT-5_AT480_AE_POL |  | *Arrhenatherum elatius* | tall oat-grass | Poland | 50°19'40"N, 20°30'39"E | MG208725 |  |
| 420 | MT-5 | MT-5_MG71_AE_POL |  | *Arrhenatherum elatius* | tall oat-grass | Poland | 49°34'15"N, 20°35'53"E | MG208726 |  |
| 421 | MT-5 | MT-5_ML657_AE_POL |  | *Arrhenatherum elatius* | tall oat-grass | Poland | 52°50'25"N, 21°49'46"E | MG208727 |  |
| 422 | MT-5 | MT-5_WS93_AE_POL |  | *Arrhenatherum elatius* | tall oat-grass | Poland | 50°43'46"N, 22°56'52"E | MG208728 |  |
| 423 | MT-5 | MT-5_WS404_AE_POL |  | *Arrhenatherum elatius* | tall oat-grass | Poland | 54°01'51"N, 18°07'19"E | MG208729 |  |
| 424 | MT-5 | MT-5_JP35_AE_POL |  | *Arrhenatherum elatius* | tall oat-grass | Poland | 52°54'31"N, 19°33'28"E | MG208730 |  |
| 425 | MT-5 | MT-5_LB9_AE_POL |  | *Arrhenatherum elatius* | tall oat-grass | Poland | 52°11'19"N, 18°19'11"E | MG208731 |  |
| 426 | MT-5 | MT-5_NW7_AE_POL |  | *Arrhenatherum elatius* | tall oat-grass | Poland | 49°50'00"N, 18°40'23"E | MG208732 |  |
| 427 | MT-5 | MT-5_NW181_AE_POL |  | *Arrhenatherum elatius* | tall oat-grass | Poland | 53°40'18"N, 15°02'23"E | MG208733 |  |
| 428 | MT-5 | MT-5_AS109_AE_POL |  | *Arrhenatherum elatius* | tall oat-grass | Poland | 52°34'10"N, 17°06'38"E | MG208734 |  |
| 429 | MT-5 | MT-5_AS1170_AE_POL | MT-5_AS1170.D2_AE_POL | *Arrhenatherum elatius* | tall oat-grass | Poland | 50°42'42"N, 22°59'06"E | MG208735 | MG076837 |
| 430 | MT-5 | MT-5_AS072_AE_POL |  | *Arrhenatherum elatius* | tall oat-grass | Poland | 53°15'16"N, 16°01'22"E | MG208736 |  |
| 431 | MT-5 | MT-5_AS073_AE_POL |  | *Arrhenatherum elatius* | tall oat-grass | Poland | 53°02'49"N, 15°50'53"E | MG208737 |  |
| 432 | MT-5 | MT-5_NW206_AE_POL |  | *Arrhenatherum elatius* | tall oat-grass | Poland | 53°08'42"N, 15°09'47"E | MG208738 |  |
| 433 | MT-5 | MT-5_oatWCM08_AE_POL | MT-5_oatWCM08.D2_AE_POL | *Arrhenatherum elatius* | tall oat-grass | Poland | 52°27'59"N, 16°56'03"E | JQ248924* | MG076838 |
| 434 | MT-5 | MT-5_oatWCM09_AE_POL | MT-5_oatWCM09.D2_AE_POL | *Arrhenatherum elatius* | tall oat-grass | Poland | 52°28'08"N, 16°56'09"E | JQ248925* | JQ918887* |
| 435 | MT-5 | MT-5_AS399_AE_POL |  | *Arrhenatherum elatius* | tall oat-grass | Poland | 52°28'40"N, 16°56'11"E | JF920093* |  |
| 436 | MT-5 | MT-5_AS407_AE_POL | MT-5_AS407.D2_AE_POL | *Arrhenatherum elatius* | tall oat-grass | Poland | 52°27'58"N, 16°56'02"E | JF920094* | JF920109* |
| 437 | MT-5 | MT-5_AS1737_AE_POL | MT-5_AS1737.D2_AE_POL | *Arrhenatherum elatius* | tall oat-grass | Poland | 52°02'23"N, 16°46'09"E | KF975397* | MG076839 |
| 438 | MT-5 | MT-5_AS1734_AE_POL | MT-5_AS1734.D2_AE_POL | *Arrhenatherum elatius* | tall oat-grass | Poland | 52°27'01"N, 17°14'53"E | KF975398* | MG076840 |
| 439 | MT-5 | MT-5_MG101_AE_POL |  | *Arrhenatherum elatius* | tall oat-grass | Poland | 51°53'26"N, 15°19'45"E | MG208739 |  |
| 440 | MT-5 | MT-5_WS247_AE_POL |  | *Arrhenatherum elatius* | tall oat-grass | Poland | 49°41'01"N, 22°44'15"E | MG208740 |  |
| 441 | MT-5 | MT-5_WS269_AE_POL |  | *Arrhenatherum elatius* | tall oat-grass | Poland | 50°45'58"N, 22°21'18"E | MG208741 |  |
| 442 | MT-5 | MT-5_AK544_AE_POL |  | *Arrhenatherum elatius* | tall oat-grass | Poland | 50°50'41"N, 20°08'38"E | MG208742 |  |
| 443 | MT-5 | MT-5_ML1667_AE_POL |  | *Arrhenatherum elatius* | tall oat-grass | Poland | 50°39'25"N, 20°01'34"E | MG208743 |  |
| 444 | MT-5 | MT-5_NW260_AE_POL |  | *Arrhenatherum elatius* | tall oat-grass | Poland | 50°39'25"N, 20°01'34"E | MG208744 |  |
| 445 | MT-5 | MT-5_WS347_AE_POL |  | *Arrhenatherum elatius* | tall oat-grass | Poland | 51°06'04"N, 19°29'34"E | MG208745 |  |
| 446 | MT-5 | MT-5_WS365_AE_POL |  | *Arrhenatherum elatius* | tall oat-grass | Poland | 51°07'13"N, 18°55'17"E | MG208746 |  |
| 447 | MT-5 | MT-5_WS395_AE_POL |  | *Arrhenatherum elatius* | tall oat-grass | Poland | 52°44'43"N, 18°10'39"E | MG208747 |  |
| 448 | MT-5 | MT-5_WS393_AE_POL |  | *Arrhenatherum elatius* | tall oat-grass | Poland | 52°44'43"N, 18°10'39"E | MG208748 |  |
| 449 | MT-5 | MT-5_WS434_AE_POL |  | *Arrhenatherum elatius* | tall oat-grass | Poland | 50°26'12"N, 18°53'40"E | MG208749 |  |
| 450 | MT-5 | MT-5_ML966_AE_POL |  | *Arrhenatherum elatius* | tall oat-grass | Poland | 54°25'02"N, 19°54'06"E | MG208750 |  |
| 451 | MT-5 | MT-5_ML1154_AE_POL |  | *Arrhenatherum elatius* | tall oat-grass | Poland | 53°26'13"N, 22°59'01"E | MG208751 |  |
| 452 | MT-5 | MT-5_ML1565_AE_POL |  | *Arrhenatherum elatius* | tall oat-grass | Poland | 53°26'13"N, 22°59'01"E | MG208752 |  |
| 453 | MT-5 | MT-5_ML1262_AE_POL |  | *Arrhenatherum elatius* | tall oat-grass | Poland | 52°50'01"N, 23°43'50"E | MG208753 |  |
| 454 | MT-5 | MT-5_NW61_AE_POL |  | *Arrhenatherum elatius* | tall oat-grass | Poland | 51°19'50"N, 17°09'18"E | MG208754 |  |
| 455 | MT-5 | MT-5_NW226_AE_POL |  | *Arrhenatherum elatius* | tall oat-grass | Poland | 52°09'49"N, 18°59'34"E | MG208755 |  |
| 456 | MT-5 | MT-5_A641_AE_POL |  | *Arrhenatherum elatius* | tall oat-grass | Poland | 50°21'44"N, 19°44'18"E | MG208756 |  |
| 457 | MT-5 | MT-5_WS431_AE_POL |  | *Arrhenatherum elatius* | tall oat-grass | Poland | 50°41'18"N, 18°46'50"E | MG208757 |  |
| 458 | MT-5 | MT-5_ML330_AE_POL |  | *Arrhenatherum elatius* | tall oat-grass | Poland | 54°07'31"N, 22°51'01"E | MG208758 |  |
| 459 | MT-5 | MT-5_ML491_AE_POL |  | *Arrhenatherum elatius* | tall oat-grass | Poland | 53°57'49"N, 22°32'44"E | MG208759 |  |
| 460 | MT-5 | MT-5_ML605_AE_POL |  | *Arrhenatherum elatius* | tall oat-grass | Poland | 49°34'34"N, 21°45'38"E | MG208760 |  |
| 461 | MT-5 | MT-5_ML841_AE_POL |  | *Arrhenatherum elatius* | tall oat-grass | Poland | 53°23'50"N, 20°18'59"E | MG208761 |  |
| 462 | MT-5 | MT-5_MG77_AE_POL |  | *Arrhenatherum elatius* | tall oat-grass | Poland | 53°48'37"N, 15°08'53"E | MG208762 |  |
| 463 | MT-6 | MT-6_AT130_ER_POL |  | *Elymus repens* | quackgrass | Poland | 52°28'57"N, 16°40'48"E | MG208763 |  |
| 464 | MT-6 | MT-6_AT174_ER_POL |  | *Elymus repens* | quackgrass | Poland | 52°52'10"N, 16°41'20"E | MG208764 |  |
| 465 | MT-6 | MT-6_AK1006_ER_POL |  | *Elymus repens* | quackgrass | Poland | 52°18'17"N, 22°56'24"E | MG208765 |  |
| 466 | MT-6 | MT-6_ML549_ER_POL |  | *Elymus repens* | quackgrass | Poland | 52°52'36"N, 21°24'02"E | MG208766 |  |
| 467 | MT-6 | MT-6_AK458_ER_POL | MT-6_AK458.D2_ER_POL | *Elymus repens* | quackgrass | Poland | 53°21'44"N, 14°26'12"E | MG208767 | MG076841 |
| 468 | MT-6 | MT-6_quaWCM10_ER_POL | MT-6_quaWCM10.D2_ER_POL | *Elymus repens* | quackgrass | Poland | 52°27'55"N, 16°55'55"E | JQ248919* | JQ918882* |
| 469 | MT-6 | MT-6_ML1750_ER_POL |  | *Elymus repens* | quackgrass | Poland | 52°10'08"N, 22°03'52"E | MG208768 |  |
| 470 | MT-6 | MT-6_ML468A_ER_POL |  | *Elymus repens* | quackgrass | Poland | 53°42'57"N, 22°24'12"E | MG208769 |  |
| 471 | MT-6 | MT-6_ML723_ER_POL |  | *Elymus repens* | quackgrass | Poland | 52°18'58"N, 23°07'54"E | MG208770 |  |
| 472 | MT-7 | MT-7_AS823_HM_POL | MT-7_AS823.D2_HM_POL | *Hordeum murinum* | wall barley | Poland | 52°13'45"N, 17°16'34"E | MG208771 | MG076842 |
| 473 | MT-7 | MT-7_AS058_HM_POL | MT-7_AS058.D2_HM_POL | *Hordeum murinum* | wall barley | Poland | 52°22'54"N, 16°56'06"E | JF920086* | JF920103* |
| 474 | MT-7 | MT-7_AS364_HM_POL |  | *Hordeum murinum* | wall barley | Poland | 51°57'39"N, 16°15'07"E | JF920087* |  |
| 475 | MT-7 | MT-7_AS720_HM_POL |  | *Hordeum murinum* | wall barley | Poland | 52°27'37"N, 16°55'25"E | KF975399* |  |
| 476 | MT-7 | MT-7_AS753_HM_POL |  | *Hordeum murinum* | wall barley | Poland | 52°23'53"N, 16°56'24"E | KF975400* |  |
| 477 | MT-7 | MT-7_AS1080_HM_POL |  | *Hordeum murinum* | wall barley | Poland | 52°22'53"N, 16°56'07"E | KF975401* |  |
| 478 | MT-7 | MT-7_barWCM11_HM_POL | MT-7_barWCM11.D2_HM_POL | *Hordeum murinum* | wall barley | Poland | 52°27'46"N, 16°55'29"E | JQ248926* | JQ918888* |
| 479 | MT-7 | MT-7_barWCM12_HM_POL | MT-7_barWCM12.D2_HM_POL | *Hordeum murinum* | wall barley | Poland | 52°27'52"N, 16°55'31"E | JQ248927* | JQ918889* |
| 480 | MT-7 | MT-7_barWCM13_HM_POL | MT-7_barWCM13.D2_HM_POL | *Hordeum murinum* | wall barley | Poland | 52°27'12"N, 16°55'42"E | JQ248928* | JQ918890* |
| 481 | MT-7 | MT-7_AS743_HM_TUR |  | *Hordeum murinum* | wall barley | Turkey | 38°00'29"N, 43°03'40"E | KC412857* |  |
| 482 | MT-7 | MT-7_AS609A_HM_AUS |  | *Hordeum murinum* | wall barley | Australia | 33°14'41"S, 148°48'54"E | MG208772 |  |
| 483 | MT-7 | MT-7_AS520_HM_AUS |  | *Hordeum murinum* | wall barley | Australia | 33°12'15"S, 148°50'32"E | JF920082* |  |
| 484 | MT-7 | MT-7_AS523_HM_AUS | MT-7_AS523.D2_HM_AUS | *Hordeum murinum* | wall barley | Australia | 33°13'37"S, 148°50'32"E | JF920083* | JF920102* |
| 485 | MT-7 | MT-7_AS528_HM_AUS |  | *Hordeum murinum* | wall barley | Australia | 33°12'36"S, 148°45'38"E | JF920084* |  |
| 486 | MT-7 | MT-7_AS532_HM_AUS |  | *Hordeum murinum* | wall barley | Australia | 33°07'16"S, 148°58'32"E | JF920085* |  |
| 487 | MT-8 | MT-8_AT11_TA_POL |  | *Triticum aestivum* | wheat | Poland | 50°29'20"N, 16°51'49"E | KC422631* |  |
| 488 | MT-8 | MT-8_AT98_TA_POL |  | *Triticum aestivum* | wheat | Poland | 51°11'02"N, 15°13'55"E | KC422632* |  |
| 489 | MT-8 | MT-8_NW293_TA_POL |  | *Triticum aestivum* | wheat | Poland | 52°30'05"N, 14°45'45"E | MG194264 |  |
| 490 | MT-8 | MT-8_NW223_TA_POL |  | *Triticum aestivum* | wheat | Poland | 52°09'35"N, 18°02'32"E | MG194265 |  |
| 491 | MT-8 | MT-8_AK314_TA_POL |  | *Triticum aestivum* | wheat | Poland | 51°32'49"N, 17°11'22"E | MG194266 |  |
| 492 | MT-8 | MT-8_AK672_TA_POL |  | *Triticum aestivum* | wheat | Poland | 51°14'60"N, 17°53'21"E | MG194267 |  |
| 493 | MT-8 | MT-8_AL121_TA_POL |  | *Triticum aestivum* | wheat | Poland | 50°03'23"N, 19°44'51"E | MG194268 |  |
| 494 | MT-8 | MT-8_A3A386_TA_POL |  | *Triticum aestivum* | wheat | Poland | 52°09'35"N, 18°02'32"E | MG194269 |  |
| 495 | MT-8 | MT-8_AL014_TA_POL |  | *Triticum aestivum* | wheat | Poland | 53°10'47"N, 18°58'39"E | MG194270 |  |
| 496 | MT-8 | MT-8_AT487_TA_POL |  | *Triticum aestivum* | wheat | Poland | 50°11'39"N, 20°24'30"E | MG194271 |  |
| 497 | MT-8 | MT-8_AT491_TA_POL |  | *Triticum aestivum* | wheat | Poland | 50°11'39"N, 20°24'30"E | MG194272 |  |
| 498 | MT-8 | MT-8_ML1322_TA_POL |  | *Triticum aestivum* | wheat | Poland | 52°43'26"N, 20°54'12"E | MG194273 |  |
| 499 | MT-8 | MT-8_NW282_TA_POL |  | *Triticum aestivum* | wheat | Poland | 52°53'23"N, 18°16'44"E | MG194274 |  |
| 500 | MT-8 | MT-8_NW294_TA_POL |  | *Triticum aestivum* | wheat | Poland | 52°30'05"N, 14°45'45"E | MG194275 |  |
| 501 | MT-8 | MT-8_NW303_TA_POL |  | *Triticum aestivum* | wheat | Poland | 52°27'42"N, 14°28'36"E | MG194276 |  |
| 502 | MT-8 | MT-8_NW308_TA_POL |  | *Triticum aestivum* | wheat | Poland | 52°27'42"N, 14°28'36"E | MG194277 |  |
| 503 | MT-8 | MT-8_NW325_TA_POL |  | *Triticum aestivum* | wheat | Poland | 50°29'08"N, 17°55'17"E | MG194278 |  |
| 504 | MT-8 | MT-8_NW349_TA_POL |  | *Triticum aestivum* | wheat | Poland | 50°40'06"N, 17°23'36"E | MG194279 |  |
| 505 | MT-8 | MT-8_NW352_TA_POL |  | *Triticum aestivum* | wheat | Poland | 50°40'06"N, 17°23'36"E | MG194280 |  |
| 506 | MT-8 | MT-8_NW381_TA_POL |  | *Triticum aestivum* | wheat | Poland | 50°31'20"N, 17°13'44"E | MG194281 |  |
| 507 | MT-8 | MT-8_NW385_TA_POL |  | *Triticum aestivum* | wheat | Poland | 50°07'01"N, 17°57'27"E | MG194282 |  |
| 508 | MT-8 | MT-8_AK636_TA_POL |  | *Triticum aestivum* | wheat | Poland | 50°35'06"N, 17°57'41"E | MG194283 |  |
| 509 | MT-8 | MT-8_AK637_TA_POL |  | *Triticum aestivum* | wheat | Poland | 50°35'06"N, 17°57'41"E | MG194284 |  |
| 510 | MT-8 | MT-8_AK639_TA_POL |  | *Triticum aestivum* | wheat | Poland | 50°35'06"N, 17°57'41"E | MG194285 |  |
| 511 | MT-8 | MT-8_AK760_TA_POL |  | *Triticum aestivum* | wheat | Poland | 54°27'54"N, 16°53'36"E | MG194286 |  |
| 512 | MT-8 | MT-8_AK766_TA_POL |  | *Triticum aestivum* | wheat | Poland | 50°32'21"N, 23°28'58"E | MG194287 |  |
| 513 | MT-8 | MT-8_AK788_TA_POL |  | *Triticum aestivum* | wheat | Poland | 50°57'04"N, 17°03'22"E | MG194288 |  |
| 514 | MT-8 | MT-8_AK939_TA_POL |  | *Triticum aestivum* | wheat | Poland | 50°57'04"N, 17°03'22"E | MG194289 |  |
| 515 | MT-8 | MT-8_NW390_TA_POL |  | *Triticum aestivum* | wheat | Poland | 50°05'35"N, 18°22'40"E | MG194290 |  |
| 516 | MT-8 | MT-8_NW394_TA_POL |  | *Triticum aestivum* | wheat | Poland | 50°05'35"N, 18°22'40"E | MG194291 |  |
| 517 | MT-8 | MT-8_WS417_TA_POL |  | *Triticum aestivum* | wheat | Poland | 50°22'34"N, 20°13'51"E | MG194292 |  |
| 518 | MT-8 | MT-8_WS427_TA_POL |  | *Triticum aestivum* | wheat | Poland | 50°22'34"N, 20°13'51"E | MG194293 |  |
| 519 | MT-8 | MT-8_AT10_TA_POL |  | *Triticum aestivum* | wheat | Poland | 50°29'20"N, 16°51'49"E | MG194294 |  |
| 520 | MT-8 | MT-8_AT60_TA_POL |  | *Triticum aestivum* | wheat | Poland | 50°32'02"N, 16°27'43"E | MG194295 |  |
| 521 | MT-8 | MT-8_AT100_TA_POL |  | *Triticum aestivum* | wheat | Poland | 51°11'02"N, 15°13'55"E | MG194296 |  |
| 522 | MT-8 | MT-8_AT104_TA_POL |  | *Triticum aestivum* | wheat | Poland | 51°11'02"N, 15°13'55"E | MG194297 |  |
| 523 | MT-8 | MT-8_AK309_TA_POL |  | *Triticum aestivum* | wheat | Poland | 50°57'04"N, 17°03'22"E | MG194298 |  |
| 524 | MT-8 | MT-8_AK350_TA_POL |  | *Triticum aestivum* | wheat | Poland | 50°28'15"N, 23°37'02"E | MG194299 |  |
| 525 | MT-8 | MT-8_AK362_TA_POL |  | *Triticum aestivum* | wheat | Poland | 50°32'21"N, 23°28'58"E | MG194300 |  |
| 526 | MT-8 | MT-8_AT292_TA_POL |  | *Triticum aestivum* | wheat | Poland | 54°27'54"N, 16°53'36"E | MG194301 |  |
| 527 | MT-8 | MT-8_AT318_TA_POL |  | *Triticum aestivum* | wheat | Poland | 53°19'30"N, 17°00'36"E | MG194302 |  |
| 528 | MT-8 | MT-8_AT321_TA_POL |  | *Triticum aestivum* | wheat | Poland | 53°19'30"N, 17°00'36"E | MG194303 |  |
| 529 | MT-8 | MT-8_WS165_TA_POL |  | *Triticum aestivum* | wheat | Poland | 50°42'18"N, 23°23'07"E | MG194304 |  |
| 530 | MT-8 | MT-8_WS196_TA_POL |  | *Triticum aestivum* | wheat | Poland | 53°07'27"N, 14°33'60"E | MG194305 |  |
| 531 | MT-8 | MT-8_WS204_TA_POL |  | *Triticum aestivum* | wheat | Poland | 53°07'27"N, 14°33'60"E | MG194306 |  |
| 532 | MT-8 | MT-8_WS210_TA_POL |  | *Triticum aestivum* | wheat | Poland | 53°07'27"N, 14°33'60"E | MG194307 |  |
| 533 | MT-8 | MT-8_AT413_TA_POL |  | *Triticum aestivum* | wheat | Poland | 53°08'42"N, 15°09'47"E | MG194308 |  |
| 534 | MT-8 | MT-8_AT409_TA_POL |  | *Triticum aestivum* | wheat | Poland | 53°08'42"N, 15°09'47"E | MG194309 |  |
| 535 | MT-8 | MT-8_ML53_TA_POL |  | *Triticum aestivum* | wheat | Poland | 52°43'26"N, 20°54'12"E | MG194310 |  |
| 536 | MT-8 | MT-8_NW47_TA_POL |  | *Triticum aestivum* | wheat | Poland | 51°49'34"N, 14°45'13"E | MG194311 |  |
| 537 | MT-8 | MT-8_NW72_TA_POL |  | *Triticum aestivum* | wheat | Poland | 51°49'07"N, 16°20'12"E | MG194312 |  |
| 538 | MT-8 | MT-8_PK40_TA_POL |  | *Triticum aestivum* | wheat | Poland | 51°16'23"N, 15°45'06"E | MG194313 |  |
| 539 | MT-8 | MT-8_AT125_TA_POL |  | *Triticum aestivum* | wheat | Poland | 52°28'57"N, 16°40'48"E | MG194314 |  |
| 540 | MT-8 | MT-8_AS1155_TA_POL |  | *Triticum aestivum* | wheat | Poland | 51°51'41"N, 17°10'08"E | MG194315 |  |
| 541 | MT-8 | MT-8_AS1133_TA_POL |  | *Triticum aestivum* | wheat | Poland | 51°51'27"N, 17°10'11"E | MG194316 |  |
| 542 | MT-8 | MT-8_AS852_TA_POL | MT-8_AS852.D2_TA_POL | *Triticum aestivum* | wheat | Poland | 51°50'17"N, 17°08'44"E | MG194317 | MG076843 |
| 543 | MT-8 | MT-8_AS1840_TA_POL | MT-8_AS1840.D2_TA_POL | *Triticum aestivum* | wheat | Poland | 52°23'36"N, 16°51'30"E | MG194318 | MG076844 |
| 544 | MT-8 | MT-8_MG7_TA_POL |  | *Triticum aestivum* | wheat | Poland | 51°11'02"N, 15°13'55"E | MG194319 |  |
| 545 | MT-8 | MT-8_AS855_TA_POL |  | *Triticum aestivum* | wheat | Poland | 51°51'36"N, 17°09'26"E | MG194320 |  |
| 546 | MT-8 | MT-8_AK4_TA_POL |  | *Triticum aestivum* | wheat | Poland | 52°28'57"N, 16°40'48"E | MG194321 |  |
| 547 | MT-8 | MT-8_ML1673_TA_POL |  | *Triticum aestivum* | wheat | Poland | 51°32'49"N, 17°11'22"E | MG194322 |  |
| 548 | MT-8 | MT-8_ML1664_TA_POL |  | *Triticum aestivum* | wheat | Poland | 51°38'28"N, 19°28'11"E | MG194323 |  |
| 549 | MT-8 | MT-8_WS315_TA_POL |  | *Triticum aestivum* | wheat | Poland | 50°23'45"N, 21°18'04"E | MG194324 |  |
| 550 | MT-8 | MT-8_WS350_TA_POL |  | *Triticum aestivum* | wheat | Poland | 51°30'58"N, 18°57'50"E | MG194325 |  |
| 551 | MT-8 | MT-8_WS380_TA_POL |  | *Triticum aestivum* | wheat | Poland | 51°42'50"N, 18°50'53"E | MG194326 |  |
| 552 | MT-8 | MT-8_PK94_TA_POL |  | *Triticum aestivum* | wheat | Poland | 53°05'47"N, 18°14'44"E | MG194327 |  |
| 553 | MT-8 | MT-8_ML1685_TA_POL |  | *Triticum aestivum* | wheat | Poland | 52°30'05"N, 14°45'45"E | MG194328 |  |
| 554 | MT-8 | MT-8_AK629_TA_POL |  | *Triticum aestivum* | wheat | Poland | 52°30'05"N, 14°45'45"E | MG194329 |  |
| 555 | MT-8 | MT-8_AT478_TA_POL |  | *Triticum aestivum* | wheat | Poland | 50°34'29"N, 17°50'55"E | MG194330 |  |
| 556 | MT-8 | MT-8_WS445_TA_POL |  | *Triticum aestivum* | wheat | Poland | 50°29'56"N, 18°38'25"E | MG194331 |  |
| 557 | MT-8 | MT-8_ML1699_TA_POL |  | *Triticum aestivum* | wheat | Poland | 51°14'60"N, 17°53'21"E | MG194332 |  |
| 558 | MT-8 | MT-8_ML1716_TA_POL |  | *Triticum aestivum* | wheat | Poland | 51°33'46"N, 17°41'38"E | MG194333 |  |
| 559 | MT-8 | MT-8_ML912_TA_POL |  | *Triticum aestivum* | wheat | Poland | 53°43'16"N, 20°18'41"E | MG194334 |  |
| 560 | MT-8 | MT-8_ML1059_TA_POL |  | *Triticum aestivum* | wheat | Poland | 51°44'31"N, 22°49'05"E | MG194335 |  |
| 561 | MT-8 | MT-8_ML1560_TA_POL |  | *Triticum aestivum* | wheat | Poland | 51°44'31"N, 22°49'05"E | MG194336 |  |
| 562 | MT-8 | MT-8_A512_TS_POL |  | *Triticosecale* | triticale | Poland | 51°30'58"N, 18°57'50"E | MG194337 |  |
| 563 | MT-8 | MT-8_A512A_TS_POL |  | *Triticosecale* | triticale | Poland | 51°30'58"N, 18°57'50"E | MG194338 |  |
| 564 | MT-8 | MT-8_AK570_TS_POL |  | *Triticosecale* | triticale | Poland | 50°49'22"N, 19°23'26"E | MG194339 |  |
| 565 | MT-8 | MT-8_AK749_TS_POL |  | *Triticosecale* | triticale | Poland | 51°32'49"N, 17°11'22"E | MG194340 |  |
| 566 | MT-8 | MT-8_AK970_TS_POL |  | *Triticosecale* | triticale | Poland | 53°26'04"N, 14°56'28"E | MG194341 |  |
| 567 | MT-8 | MT-8_AK192_TS_POL |  | *Triticosecale* | triticale | Poland | 51°33'15"N, 15°49'58"E | MG194342 |  |
| 568 | MT-8 | MT-8_AK289_TS_POL |  | *Triticosecale* | triticale | Poland | 51°35'45"N, 16°53'03"E | MG194343 |  |
| 569 | MT-8 | MT-8_NW164_TS_POL |  | *Triticosecale* | triticale | Poland | 54°01'22"N, 17°21'31"E | MG194344 |  |
| 570 | MT-8 | MT-8_AS1723_TS_POL | MT-8_AS1723.D2_TS_POL | *Triticosecale* | triticale | Poland | 52°26'17"N, 17°14'26"E | MG194345 | MG076845 |
| 571 | MT-8 | MT-8_AT62_TS_POL |  | *Triticosecale* | triticale | Poland | 50°43'46"N, 15°56'43"E | MG194346 |  |
| 572 | MT-8 | MT-8_AS860_TS_POL | MT-8_AS860.D2_TS_POL | *Triticosecale* | triticale | Poland | 51°50'53"N, 17°09'06"E | KC422635* | MG076846 |
| 573 | MT-8 | MT-8_AT121_TS_POL |  | *Triticosecale* | triticale | Poland | 52°28'57"N, 16°40'48"E | MG194347 |  |
| 574 | MT-8 | MT-8_PK42_TS_POL |  | *Triticosecale* | triticale | Poland | 51°15'42"N, 16°22'09"E | MG194348 |  |
| 575 | MT-8 | MT-8_NW167_TS_POL |  | *Triticosecale* | triticale | Poland | 53°14'34"N, 17°32'19"E | MG194349 |  |
| 576 | MT-8 | MT-8_AK528_TS_POL |  | *Triticosecale* | triticale | Poland | 51°18'21"N, 21°13'54"E | MG194350 |  |
| 577 | MT-8 | MT-8_ML1662_TS_POL |  | *Triticosecale* | triticale | Poland | 51°18'21"N, 21°13'54"E | MG194351 |  |
| 578 | MT-8 | MT-8_AT459_TS_POL |  | *Triticosecale* | triticale | Poland | 50°41'50"N, 20°28'48"E | MG194352 |  |
| 579 | MT-8 | MT-8_AK565_TS_POL |  | *Triticosecale* | triticale | Poland | 51°51'26"N, 18°38'39"E | MG194353 |  |
| 580 | MT-8 | MT-8_EP6_TS_POL |  | *Triticosecale* | triticale | Poland | 53°10'32"N, 19°59'43"E | MG194354 |  |
| 581 | MT-8 | MT-8_EP61_TS_POL |  | *Triticosecale* | triticale | Poland | 52°06'03"N, 23°21'37"E | MG194355 |  |
| 582 | MT-8 | MT-8_ML1557_TS_POL |  | *Triticosecale* | triticale | Poland | 51°09'24"N, 23°48'00"E | MG194356 |  |
| 583 | MT-8 | MT-8_ML998_TS_POL |  | *Triticosecale* | triticale | Poland | 51°48'17"N, 22°31'59"E | MG194357 |  |
| 584 | MT-8 | MT-8_NW20_SR_POL |  | *Secale cereale* | rye | Poland | 51°56'14"N, 16°01'27"E | KP973943* |  |
| 585 | MT-8 | MT-8_ML99_ER_POL |  | *Elymus repens* | quackgrass | Poland | 52°12'29"N, 20°30'02"E | MG194358 |  |
| 586 | MT-8 | MT-8_LB84_ER_POL |  | *Elymus repens* | quackgrass | Poland | 50°59'17"N, 20°53'18"E | MG194359 |  |
| 587 | MT-8 | MT-8_LB240_ER_POL |  | *Elymus repens* | quackgrass | Poland | 50°31'20"N, 17°13'44"E | MG194360 |  |
| 588 | MT-8 | MT-8_PK71_ER_POL |  | *Elymus repens* | quackgrass | Poland | 51°32'49"N, 17°11'22"E | MG194361 |  |
| 589 | MT-8 | MT-8_AK275_ER_POL |  | *Elymus repens* | quackgrass | Poland | 51°32'49"N, 17°11'22"E | MG194362 |  |
| 590 | MT-8 | MT-8_NW23_ER_POL |  | *Elymus repens* | quackgrass | Poland | 51°15'42"N, 16°22'09"E | MG194363 |  |
| 591 | MT-8 | MT-8_AK464_ER_POL |  | *Elymus repens* | quackgrass | Poland | 49°47'30"N, 20°52'59"E | MG194364 |  |
| 592 | MT-8 | MT-8_AT431_ER_POL |  | *Elymus repens* | quackgrass | Poland | 49°43'25"N, 21°58'38"E | MG194365 |  |
| 593 | MT-8 | MT-8_AK472_ER_POL |  | *Elymus repens* | quackgrass | Poland | 50°22'44"N, 22°22'11"E | MG194366 |  |
| 594 | MT-8 | MT-8_AK608_ER_POL |  | *Elymus repens* | quackgrass | Poland | 50°39'25"N, 20°01'34"E | MG194367 |  |
| 595 | MT-8 | MT-8_AT471_ER_POL |  | *Elymus repens* | quackgrass | Poland | 50°37'27"N, 19°34'22"E | MG194368 |  |
| 596 | MT-8 | MT-8_AL003_ER_POL |  | *Elymus repens* | quackgrass | Poland | 53°52'16"N, 18°30'20"E | MG194369 |  |
| 597 | MT-8 | MT-8_AL006_ER_POL |  | *Elymus repens* | quackgrass | Poland | 53°52'16"N, 18°30'20"E | MG194370 |  |
| 598 | MT-8 | MT-8_LB198_ER_POL |  | *Elymus repens* | quackgrass | Poland | 54°36'21"N, 17°15'28"E | MG194371 |  |
| 599 | MT-8 | MT-8_LB211_ER_POL |  | *Elymus repens* | quackgrass | Poland | 53°21'01"N, 18°43'19"E | MG194372 |  |
| 600 | MT-8 | MT-8_LB235_ER_POL |  | *Elymus repens* | quackgrass | Poland | 52°27'42"N, 14°28'36"E | MG194373 |  |
| 601 | MT-8 | MT-8_NW342_ER_POL |  | *Elymus repens* | quackgrass | Poland | 50°40'06"N, 17°23'36"E | MG194374 |  |
| 602 | MT-8 | MT-8_AT508_ER_POL |  | *Elymus repens* | quackgrass | Poland | 50°29'56"N, 18°38'25"E | MG194375 |  |
| 603 | MT-8 | MT-8_ML1741_ER_POL |  | *Elymus repens* | quackgrass | Poland | 51°01'16"N, 17°32'17"E | MG194376 |  |
| 604 | MT-8 | MT-8_LB335_ER_POL |  | *Elymus repens* | quackgrass | Poland | 51°14'60"N, 17°53'21"E | MG194377 |  |
| 605 | MT-8 | MT-8_ML858_ER_POL |  | *Elymus repens* | quackgrass | Poland | 53°01'07"N, 21°01'14"E | MG194378 |  |
| 606 | MT-8 | MT-8_EP11_ER_POL |  | *Elymus repens* | quackgrass | Poland | 53°10'32"N, 19°59'43"E | MG194379 |  |
| 607 | MT-8 | MT-8_EP26_ER_POL |  | *Elymus repens* | quackgrass | Poland | 53°24'05"N, 19°51'54"E | MG194380 |  |
| 608 | MT-8 | MT-8_ML1091_ER_POL |  | *Elymus repens* | quackgrass | Poland | 51°27'30"N, 22°40'45"E | MG194381 |  |
| 609 | MT-8 | MT-8_ML1115_ER_POL |  | *Elymus repens* | quackgrass | Poland | 53°07'03"N, 22°50'58"E | MG194382 |  |
| 610 | MT-8 | MT-8_NW155_ER_POL |  | *Elymus repens* | quackgrass | Poland | 50°42'18"N, 23°23'07"E | MG194383 |  |
| 611 | MT-8 | MT-8_MG140_AE_POL |  | *Arrhenatherum elatius* | tall oat-grass | Poland | 50°35'18"N, 24°02'20"E | MG194384 |  |
| 612 | MT-8 | MT-8_AT479_AE_POL |  | *Arrhenatherum elatius* | tall oat-grass | Poland | 50°19'40"N, 20°30'39"E | MG194385 |  |
| 613 | MT-8 | MT-8_AK191_AE_POL |  | *Arrhenatherum elatius* | tall oat-grass | Poland | 51°16'23"N, 15°45'06"E | MG194386 |  |
| 614 | MT-8 | MT-8_ML1687_AE_POL |  | *Arrhenatherum elatius* | tall oat-grass | Poland | 52°27'42"N, 14°28'36"E | MG194387 |  |
| 615 | MT-8 | MT-8_NW319_AE_POL |  | *Arrhenatherum elatius* | tall oat-grass | Poland | 52°27'42"N, 14°28'36"E | MG194388 |  |
| 616 | MT-8 | MT-8_AL092_AE_POL |  | *Arrhenatherum elatius* | tall oat-grass | Poland | 50°35'06"N, 17°57'41"E | MG194389 |  |
| 617 | MT-8 | MT-8_NW330_AE_POL |  | *Arrhenatherum elatius* | tall oat-grass | Poland | 50°29'08"N, 17°55'17"E | MG194390 |  |
| 618 | MT-8 | MT-8_NW396_AE_POL |  | *Arrhenatherum elatius* | tall oat-grass | Poland | 50°29'56"N, 18°38'25"E | MG194391 |  |
| 619 | MT-8 | MT-8_AT205_AS_POL |  | *Avena sativa* | oat | Poland | 51°32'49"N, 17°11'22"E | MG194392 |  |
| 620 | MT-8 | MT-8_AT206_AS_POL |  | *Avena sativa* | oat | Poland | 51°32'49"N, 17°11'22"E | MG194393 |  |
| 621 | MT-8 | MT-8_WS76_AS_POL |  | *Avena sativa* | oat | Poland | 50°43'46"N, 22°56'52"E | MG194394 |  |
| 622 | MT-8 | MT-8_AT105_BI_POL |  | *Bromus inermis* | smooth brome | Poland | 52°24'02"N, 16°37'31"E | KC430098* |  |
| 623 | MT-8 | MT-8_AK339_BI_POL |  | *Bromus inermis* | smooth brome | Poland | 50°56'31"N, 23°18'28"E | MG194395 |  |
| 624 | MT-8 | MT-8_NW321_BI_POL |  | *Bromus inermis* | smooth brome | Poland | 52°27'42"N, 14°28'36"E | MG194396 |  |
| 625 | MT-8 | MT-8_EP63_BI_POL |  | *Bromus inermis* | smooth brome | Poland | 52°02'50"N, 23°35'19"E | MG194397 |  |
| 626 | MT-8 | MT-8_AS1499_ZM_POL |  | *Zea mays* | maize | Poland | 51°50'46"N, 17°09'15"E | MG194398 |  |
| 627 | MT-8 | MT-8_AS1792_ZM_POL |  | *Zea mays* | maize | Poland | 52°26'17"N, 17°14'26"E | MG194399 |  |
| 628 | MT-8 | MT-8_AS1337_ZM_POL |  | *Zea mays* | maize | Poland | 51°50'46"N, 17°09'15"E | MG194400 |  |
| 629 | MT-8 | MT-8_AS1799_ZM_POL | MT-8_AS1799.D2_ZM_POL | *Zea mays* | maize | Poland | 52°02'40"N, 16°46'02"E | MG194401 | MG076847 |
| 630 | MT-8 | MT-8_AS1893_BH_POL | MT-8_AS1893.D2_BH_POL | *Bromus hordeaceus* | soft brome | Poland | 52°23'36"N, 16°51'30"E | MG194402 | MG076848 |
| 631 | MT-8 | MT-8_AS1807_ZM_POL | MT-8_AS1807.D2_ZM_POL | *Zea mays* | maize | Poland | 51°50'57"N, 17°10'24"E | MG194403 | MG076849 |
| 632 | MT-8 | MT-8_AS1112_ZM_POL | MT-8_AS1112.D2_ZM_POL | *Zea mays* | maize | Poland | 52°26'02"N, 16°45'08"E | MG194404 | MG076850 |
| 633 | MT-8 | MT-8_AS1943_ZM_POL | MT-8_AS1943.D2_ZM_POL | *Zea mays* | maize | Poland | 51°15'14"N, 22°16'31"E | MG194405 | MG076851 |
| 634 | MT-8 | MT-8_AS921_ZM_POL | MT-8_AS921.D2_ZM_POL | *Zea mays* | maize | Poland | 52°26'06"N, 16°45'11"E | MG194406 | MG076852 |
| 635 | MT-8 | MT-8_AS943_ZM_POL | MT-8_AS944.D2_ZM_POL | *Zea mays* | maize | Poland | 51°41'54"N, 17°10'55"E | MG194407 | MG076853 |
| 636 | MT-8 | MT-8_AS910_ZM_POL | MT-8_AS910.D2_ZM_POL | *Zea mays* | maize | Poland | 52°26'09"N, 16°45'00"E | MG194408 | MG076854 |
| 637 | MT-8 | MT-8_AS848_ZM_POL |  | *Zea mays* | maize | Poland | 51°50'57"N, 17°09'15"E | MG194409 |  |
| 638 | MT-8 | MT-8_AS914_ZM_POL | MT-8_AS914.D2_ZM_POL | *Zea mays* | maize | Poland | 52°27'27"N, 17°13'18"E | KC422636* | MG076855 |
| 639 | MT-8 | MT-8_AS1778_ZM_POL |  | *Zea mays* | maize | Poland | 52°26'55"N, 16°45'20"E | MG194410 |  |
| 640 | MT-8 | MT-8_AS1114_HM_POL | MT-8_AS1114.D2_HM_POL | *Hordeum murinum* | wall barley | Poland | 52°27'47"N, 16°55'36"E | MG194411 | MG076856 |
| 641 | MT-8 | MT-8_AT153A_HV_POL |  | *Hordeum vulgare* | barley | Poland | 52°20'27"N, 17°20'00"E | MG194412 |  |
| 642 | MT-8 | MT-8_AT155A_HV_POL |  | *Hordeum vulgare* | barley | Poland | 52°20'27"N, 17°20'00"E | MG194413 |  |
| 643 | MT-8 | MT-8_NW14_HV_POL |  | *Hordeum vulgare* | barley | Poland | 51°26'29"N, 16°21'32"E | MG194414 |  |
| 644 | MT-8 | MT-8_AS1721_HV_GER |  | *Hordeum vulgare* | barley | Germany | 51°47'02"N, 11°17'60"E | MG194415 |  |
| 645 | MT-8 | MT-8_AS1719_HV_GER |  | *Hordeum vulgare* | barley | Germany | 51°47'02"N, 11°17'60"E | MG194416 |  |
| 646 | MT-8 | MT-8_AS529_TA_AUS |  | *Triticum aestivum* | wheat | Australia | 33°17'47"S, 148°54'57"E | JF920078* |  |
| 647 | MT-8 | MT-8_AS596_TA_AUS | MT-8_AS596.D2_TA_AUS | *Triticum aestivum* | wheat | Australia | 33°12'48"S, 148°45'37"E | JF920079* | JF920100* |
| 648 | MT-8 | MT-8_AS1102_TA_TUR | MT-8_AS1102.D2_TA_TUR | *Triticum aestivum* | wheat | Turkey | 39°01'41"N, 43°21'42"E | KC412861 * | KM280951* |
| 649 | MT-8 | MT-8_AS1103_TA_TUR | MT-8_AS1103.D2_TA_TUR | *Triticum aestivum* | wheat | Turkey | 39°01'41"N, 43°21'42"E | KC412862* | KM280952* |
| 650 | MT-8 | MT-8_USA.TX_TA_USA |  | *Triticum aestivum* | wheat | USA | n/a | JX102049* |  |
| 651 | MT-9 | MT-9_MG14_BI_POL |  | *Bromus inermis* | smooth brome | Poland | 52°24'02"N, 16°37'31"E | KC422633* |  |
| 652 | MT-9 | MT-9_AT108_BI_POL |  | *Bromus inermis* | smooth brome | Poland | 52°24'02"N, 16°37'31"E | KC422634* |  |
| 653 | MT-9 | MT-9_ML1372_BI_POL |  | *Bromus inermis* | smooth brome | Poland | 53°25'01"N, 21°22'14"E | MG208773 |  |
| 654 | MT-9 | MT-9_ML217_BI_POL |  | *Bromus inermis* | smooth brome | Poland | 53°25'01"N, 21°22'14"E | MG208774 |  |
| 655 | MT-9 | MT-9_ML1306_BI_POL |  | *Bromus inermis* | smooth brome | Poland | 52°51'26"N, 22°32'38"E | MG208775 |  |
| 656 | MT-9 | MT-9_AT161_BI_POL |  | *Bromus inermis* | smooth brome | Poland | 52°20'27"N, 17°20'00"E | MG208776 |  |
| 657 | MT-9 | MT-9_AT167_BI_POL |  | *Bromus inermis* | smooth brome | Poland | 52°20'27"N, 17°20'00"E | MG208777 |  |
| 658 | MT-9 | MT-9_AT242_BI_POL |  | *Bromus inermis* | smooth brome | Poland | 51°51'29"N, 16°57'33"E | MG208778 |  |
| 659 | MT-9 | MT-9_AT429_BI_POL |  | *Bromus inermis* | smooth brome | Poland | 51°35'45"N, 16°53'03"E | MG208779 |  |
| 660 | MT-9 | MT-9_MG142_BI_POL |  | *Bromus inermis* | smooth brome | Poland | 50°54'25"N, 23°57'34"E | MG208780 |  |
| 661 | MT-9 | MT-9_ML366_BI_POL |  | *Bromus inermis* | smooth brome | Poland | 54°07'31"N, 22°51'01"E | MG208781 |  |
| 662 | MT-9 | MT-9_JP38_BI_POL |  | *Bromus inermis* | smooth brome | Poland | 52°54'31"N, 19°33'28"E | MG208782 |  |
| 663 | MT-9 | MT-9_JP73_BI_POL |  | *Bromus inermis* | smooth brome | Poland | 52°08'09"N, 21°05'51"E | MG208783 |  |
| 664 | MT-9 | MT-9_ML191_BI_POL |  | *Bromus inermis* | smooth brome | Poland | 52°40'30"N, 19°33'17"E | MG208784 |  |
| 665 | MT-9 | MT-9_ML457A_BI_POL |  | *Bromus inermis* | smooth brome | Poland | 53°57'49"N, 22°32'44"E | MG208785 |  |
| 666 | MT-9 | MT-9_ML818_BI_POL |  | *Bromus inermis* | smooth brome | Poland | 53°09'43"N, 20°26'38"E | MG208786 |  |
| 667 | MT-9 | MT-9_ML1412_BI_POL |  | *Bromus inermis* | smooth brome | Poland | 54°04'50"N, 23°21'60"E | MG208787 |  |
| 668 | MT-9 | MT-9_ML1462_BI_POL |  | *Bromus inermis* | smooth brome | Poland | 53°43'10"N, 21°04'09"E | MG208788 |  |
| 669 | MT-9 | MT-9_ML1715_BI_POL |  | *Bromus inermis* | smooth brome | Poland | 53°43'10"N, 21°04'09"E | MG208789 |  |
| 670 | MT-9 | MT-9_ML1542_BI_POL |  | *Bromus inermis* | smooth brome | Poland | 52°18'09"N, 20°05'35"E | MG208790 |  |
| 671 | MT-9 | MT-9_NW311_BI_POL |  | *Bromus inermis* | smooth brome | Poland | 54°02'34"N, 18°52'13"E | MG208791 |  |
| 672 | MT-9 | MT-9_NW312_BI_POL |  | *Bromus inermis* | smooth brome | Poland | 54°02'34"N, 18°52'13"E | MG208792 |  |
| 673 | MT-9 | MT-9_WS07_BI_POL |  | *Bromus inermis* | smooth brome | Poland | 52°08'19"N, 17°03'48"E | MG208793 |  |
| 674 | MT-9 | MT-9_WS08_BI_POL |  | *Bromus inermis* | smooth brome | Poland | 52°08'19"N, 17°03'48"E | MG208794 |  |
| 675 | MT-9 | MT-9_WS101_BI_POL |  | *Bromus inermis* | smooth brome | Poland | 50°43'46"N, 22°56'52"E | MG208795 |  |
| 676 | MT-9 | MT-9_WS279_BI_POL |  | *Bromus inermis* | smooth brome | Poland | 52°21'07"N, 19°18'57"E | MG208796 |  |
| 677 | MT-9 | MT-9_WS324_BI_POL |  | *Bromus inermis* | smooth brome | Poland | 51°37'53"N, 20°34'57"E | MG208797 |  |
| 678 | MT-9 | MT-9_MG37_BI_POL |  | *Bromus inermis* | smooth brome | Poland | 52°20'27"N, 17°20'00"E | MG208798 |  |
| 679 | MT-9 | MT-9_AL085_BI_POL |  | *Bromus inermis* | smooth brome | Poland | 53°21'01"N, 18°43'19"E | MG208799 |  |
| 680 | MT-9 | MT-9_AS113_BI_POL |  | *Bromus inermis* | smooth brome | Poland | 52°33'41"N, 17°05'51"E | MG208800 |  |
| 681 | MT-9 | MT-9_AS338_BI_POL |  | *Bromus inermis* | smooth brome | Poland | 52°28'01"N, 16°32'24"E | MG208801 |  |
| 682 | MT-9 | MT-9_AS339_BI_POL |  | *Bromus inermis* | smooth brome | Poland | 52°28'11"N, 16°32'40"E | MG208802 |  |
| 683 | MT-9 | MT-9_AS1765_BI_POL | MT-9_AS1765.D2_BI_POL | *Bromus inermis* | smooth brome | Poland | 52°13'03"N, 17°13'09"E | MG208803 | MG076857 |
| 684 | MT-9 | MT-9_AS1212_BI_POL | MT-9_AS1212.D2_BI_POL | *Bromus inermis* | smooth brome | Poland | 51°58'56"N, 23°15'54"E | MG208804 | MG076858 |
| 685 | MT-9 | MT-9_AS400_BI_POL | MT-9_AS400.D2_BI_POL | *Bromus inermis* | smooth brome | Poland | 52°27'01"N, 16°36'44"E | JF920091* | JF920107* |
| 686 | MT-9 | MT-9_ML1402_BI_POL |  | *Bromus inermis* | smooth brome | Poland | 54°12'32"N, 21°46'07"E | MG208805 |  |
| 687 | MT-9 | MT-9_ML1602_BI_POL |  | *Bromus inermis* | smooth brome | Poland | 52°51'26"N, 22°32'38"E | MG208806 |  |
| 688 | MT-9 | MT-9_ML1562_BI_POL |  | *Bromus inermis* | smooth brome | Poland | 51°27'30"N, 22°40'45"E | MG208807 |  |
| 689 | MT-9 | MT-9_ML1748_BI_POL |  | *Bromus inermis* | smooth brome | Poland | 53°57'49"N, 22°32'44"E | MG208808 |  |
| 690 | MT-9 | MT-9_AK342_BI_POL |  | *Bromus inermis* | smooth brome | Poland | 50°56'31"N, 23°18'28"E | MG208809 |  |
| 691 | MT-9 | MT-9_ML470_BI_POL |  | *Bromus inermis* | smooth brome | Poland | 53°52'22"N, 22°15'50"E | MG208810 |  |
| 692 | MT-9 | MT-9_JP43_BI_POL |  | *Bromus inermis* | smooth brome | Poland | 54°11'08"N, 21°09'14"E | MG208811 |  |
| 693 | MT-9 | MT-9_MG135_BI_POL |  | *Bromus inermis* | smooth brome | Poland | 50°42'18"N, 23°23'07"E | MG208812 |  |
| 694 | MT-9 | MT-9_ML481A_BI_POL |  | *Bromus inermis* | smooth brome | Poland | 53°42'57"N, 22°24'12"E | MG208813 |  |
| 695 | MT-10 | MT-10_MG50_BI_POL | MT-10_MG50.D2_BI_POL | *Bromus inermis* | smooth brome | Poland | 52°18'51"N, 16°21'57"E | MG208814 | MG076859 |
| 696 | MT-10 | MT-10_WS40_BI_POL |  | *Bromus inermis* | smooth brome | Poland | 51°42'31"N, 17°52'41"E | MG208815 |  |
| 697 | MT-10 | MT-10_WS38_BI_POL |  | *Bromus inermis* | smooth brome | Poland | 51°42'31"N, 17°52'41"E | MG208816 |  |
| 698 | MT-10 | MT-10_AS089_BI_POL |  | *Bromus inermis* | smooth brome | Poland | 52°40'10"N, 17°30'01"E | MG208817 |  |
| 699 | MT-10 | MT-10_ML556_BI_POL |  | *Bromus inermis* | smooth brome | Poland | 52°52'36"N, 21°24'02"E | MG208818 |  |
| 700 | MT-11 | MT-11_AS820_ZM_POL | MT-11_AS819.D2_ZM_POL | *Zea mays* | maize | Poland | 52°26'53"N, 16°45'33"E | MG208819 | MG076860 |
| 701 | MT-11 | MT-11_AS833_EC_POL | MT-11_AS833.D2_EC_POL | *Echinochloa crus-galli* | cockspur | Poland | 52°12'33"N, 17°26'37"E | MG208820 | MG076861 |
| 702 | MT-12 | MT-12_AK155_AE_POL | MT-12_AK155.D2_AE_POL | *Arrhenatherum elatius* | tall oat-grass | Poland | 52°01'32"N, 16°45'43"E | MG208821 | MG076862 |
| 703 | MT-12 | MT-12_AL098_AE_POL |  | *Arrhenatherum elatius* | tall oat-grass | Poland | 50°05'35"N, 18°22'40"E | MG208822 |  |
| 704 | MT-12 | MT-12_AK800_AE_POL |  | *Arrhenatherum elatius* | tall oat-grass | Poland | 53°43'20"N, 16°35'49"E | MG208823 |  |
| 705 | MT-12 | MT-12_ML787_AE_POL |  | *Arrhenatherum elatius* | tall oat-grass | Poland | 52°18'09"N, 20°05'35"E | MG208824 |  |
| 706 | MT-12 | MT-12_MG29_ER_POL | MT-12_MG29.D2_ER_POL | *Elymus repens* | quackgrass | Poland | 52°18'19"N, 15°39'43"E | MG208825 | MG076863 |
| 707 | MT-12 | MT-12_AK227_ER_POL |  | *Elymus repens* | quackgrass | Poland | 51°18'10"N, 16°36'39"E | MG208826 |  |
| 708 | MT-13 | MT-13_ML83_ER_POL |  | *Elymus repens* | quackgrass | Poland | 52°25'17"N, 21°13'43"E | MG208827 |  |
| 709 | MT-13 | MT-13_ML79_ER_POL |  | *Elymus repens* | quackgrass | Poland | 52°25'17"N, 21°13'43"E | MG208828 |  |
| 710 | MT-13 | MT-13_A602_ER_POL | MT-13_A602.D2_ER_POL | *Elymus repens* | quackgrass | Poland | 50°29'08"N, 17°55'17"E | MG208829 | MG076864 |
| 711 | MT-13 | MT-13_ML124_ER_POL |  | *Elymus repens* | quackgrass | Poland | 52°13'54"N, 19°57'34"E | MG208830 |  |
| 712 | MT-13 | MT-13_PK87_ER_POL |  | *Elymus repens* | quackgrass | Poland | 52°39'11"N, 17°53'54"E | MG208831 |  |
| 713 | MT-13 | MT-13_WS250_AE_POL | MT-13_WS250.D2_AE_POL | *Arrhenatherum elatius* | tall oat-grass | Poland | 49°41'01"N, 22°44'15"E | MG208832 | MG076865 |
| 714 | MT-13 | MT-13_D3A602_ER_POL |  | *Elymus repens* | quackgrass | Poland | 50°29'08"N, 17°55'17"E | MG208833 |  |
| 715 | MT-13 | MT-13_E3A470_ER_POL | MT-13_E3A470.D2_ER_POL | *Elymus repens* | quackgrass | Poland | 51°08'40"N, 19°49'20"E | MG208834 | MG076866 |
| 716 | MT-13 | MT-13_ML9_ER_POL |  | *Elymus repens* | quackgrass | Poland | 52°08'18"N, 20°18'29"E | MG208835 |  |
| 717 | MT-13 | MT-13_ML33_ER_POL |  | *Elymus repens* | quackgrass | Poland | 52°30'15"N, 20°33'17"E | MG208836 |  |
| 718 | MT-13 | MT-13_ML71_ER_POL |  | *Elymus repens* | quackgrass | Poland | 52°34'53"N, 21°19'31"E | MG208837 |  |
| 719 | MT-13 | MT-13_ML78_ER_POL |  | *Elymus repens* | quackgrass | Poland | 52°25'17"N, 21°13'43"E | MG208838 |  |
| 720 | MT-13 | MT-13_ML932_ER_POL |  | *Elymus repens* | quackgrass | Poland | 53°06'25"N, 19°25'33"E | MG208839 |  |
| 721 | MT-14 | MT-14_ML204_BI_POL | MT-14_ML204.D2_BI_POL | *Bromus inermis* | smooth brome | Poland | 52°59'18"N, 19°47'50"E | MG208840 | MG076867 |
| 722 | MT-14 | MT-14_ML1485_BI_POL | MT-14_ML1485.D2_BI_POL | *Bromus inermis* | smooth brome | Poland | 53°57'49"N, 22°32'44"E | MG208841 | MG076868 |
| 723 | MT-14 | MT-14_ML944_BI_POL |  | *Bromus inermis* | smooth brome | Poland | 53°06'25"N, 19°25'33"E | MG208842 |  |
| 724 | MT-15 | MT-15_NW13_TA_POL | MT-15_NW13.D2_TA_POL | *Triticum aestivum* | wheat | Poland | 51°38'45"N, 15°53'55"E | MG208843 |  |
| 725 | MT-16 | MT-16_AS1207_PP_POL | MT-16_AS1206.D2_PP_POL | *Phleum pratense* | timothy-grass | Poland | 51°51'31"N, 17°09'35"E | MG208844 |  |
| 726 | MT-16 | MT-16_AS1532_PP_POL | MT-16_AS1532.D2_PP_POL | *Phleum pratense* | timothy-grass | Poland | 51°51'31"N, 17°09'35"E | MG208845 |  |
| 727 | MT-16 | MT-16_AS1739_PP_POL | MT-16_AS1739.D2_PP_POL | *Phleum pratense* | timothy-grass | Poland | 50°43'45"N, 22°56'50"E | MG208846 |  |
| 728 | MT-17 | MT-17_AS1105_TA_TUR |  | *Triticum aestivum* | wheat | Turkey | 39°56'47"N, 43°58'06"E | KC412863* |  |
| 729 | MT-17 | MT-17_AS1107_TA_TUR | MT-17_AS1107.D2_TA_TUR | *Triticum aestivum* | wheat | Turkey | 39°33'37"N, 44°05'58"E | KC412866* | KM280955* |
| 730 | MT-18 | MT-18_AS490b_BC_TUR | MT-18_AS490b.D2_BC_TUR | *Bromus cappadocius* | soft brome | Turkey | 37°19'00"N, 43°28'00"E | KC412849* | KM280946* |
| 731 | MT-18 | MT-18_AS440_BT_TUR | MT-18_AS440.D2_BT_TUR | *Bromus tomentellus* |  | Turkey | 38°18'60"N, 43°48'03"E | KC412850* | KM280945* |
| 732 | MT-19 | MT-19_AS741_PB_TUR | MT-19_AS741.D2_PB_TUR | *Poa bulbosa* | bulbous bluegrass | Turkey | 38°10'37"N, 43°56'58"E | KC412860* | KM280958* |
| 733 | MT-20 | MT-20_AS729_AC_TUR | MT-20_AS729.D2_AC_TUR | *Aegilops cylindrica* | jointed goatgrass | Turkey | 38°30'30"N, 43°22'32"E | KC412858* | KM280947* |
| 734 | MT-21 | MT-21_AS733_HM_TUR | MT-21_AS733.D2_HM_TUR | *Hordeum murinum* | wall barley | Turkey | 38°19'60"N, 43°25'60"E | KC412854* | KM280950* |
| 735 | MT-22 | MT-22_AS437_BA_TUR | MT-22_AS437.D2_BA_TUR | *Bromus arvensis* | field brome | Turkey | 38°21'20"N, 43°39'25"E | KC412848* | KM280943* |
| 736 | MT-23 | MT-23_AS742_Hsp_TUR | MT-23_AS742.D2_Hsp_TUR | *Hordeum* sp. |  | Turkey | 39°33'37"N, 44°05'58"E | KC412856* | KM280959* |
| 737 | MT-23 | MT-23_AS432_ACR_TUR | MT-23_AS432.D2_ACR_TUR | *Agropyron cristatum* | crested wheat grass | Turkey | 38°19'32"N, 43°24'27"E | KC412847* | KM280942* |
| 738 | MT-23 | MT-23_AS732_HGI_TUR | MT-23_AS732.D2_HGI_TUR | *Hordeum giganteum* | giant barley | Turkey | 38°19'60"N, 43°25'60"E | KC412853* | KM280949* |
| 739 | MT-24 | MT-24_AS1108_EO_TUR | MT-24_AS1108.D2_EO_TUR | *Eremopyrum orientale* | oriental false wheatgrass | Turkey | 40°30'44"N, 43°34'22"E | KM280939* | KM280956* |
| 740 | MT-25 | MT-25_AS446_EH_TUR | MT-25_AS446.D2_EH_TUR | *Elymus hispidus* | wheat grass | Turkey | 39°33'37"N, 44°05'58"E | KC412851* | KM280941* |
| 741 | MT-25 | MT-25_AS1104_TA_TUR | MT-25_AS1104.D2_TA_TUR | *Triticum aestivum* | wheat | Turkey | 38°25'16"N, 42°08'25"E | KC412864* | KM280953* |
| 742 | MT-26 | MT-26_AS731_SC_TUR | MT-26_AS731.D2_SC_TUR | *Secale ciliatoglume* |  | Turkey | 39°01'41"N, 43°21'42"E | KC412859* | KM280948* |
| 743 | MT-26 | MT-26_AS740_HO_TUR | MT-26_AS740.D2_HO_TUR | *Hordeum violaceum* |  | Turkey | 38°23'16"N, 42°47'32"E | KC412852* | KM280957* |
| 744 | MT-27 | MT-27_WS402_TA_POL | MT-27_WS402.D2_TA_POL | *Triticum aestivum* | wheat | Poland | 54°10'06"N, 18°39'19"E | MG208847 | MG076873 |
| 745 | MT-28 | MT-28_ML996_AE_POL |  | *Arrhenatherum elatius* | tall oat-grass | Poland | 51°29'30"N, 23°17'14"E | MG208848 |  |
| 746 | MT-29 | MT-29_MG121_AE_POL | MT-29_MG121.D2_AE_POL | *Arrhenatherum elatius* | tall oat-grass | Poland | 53°43'20"N, 16°35'49"E | MG208849 | MG076874 |
| 747 | MT-29 | MT-29_ML1652_AE_POL | MT-29_ML1652.D2_AE_POL | *Arrhenatherum elatius* | tall oat-grass | Poland | 53°43'20"N, 16°35'49"E | MG208850 | MG076875 |
