# Supplementary material for: Genetics of lineage diversification and the evolution of host usage in the economically important wheat curl mite, Aceria tosichella Keifer, 1969
Source: BMC Evol Biol. 2018 Aug 7;18:122. doi: 10.1186/s12862-018-1234-x (PMC6081818; doi:10.1186/s12862-018-1234-x)
Supplement: Supplementary file 3 — Statistics for genetic diversity of WCM genetic lineages. (DOCX 738 kb) [file 12862_2018_1234_MOESM3_ESM.docx]

**Additional file 3:** Phylogenetic trees for wheat curl mite sequence datasets

**
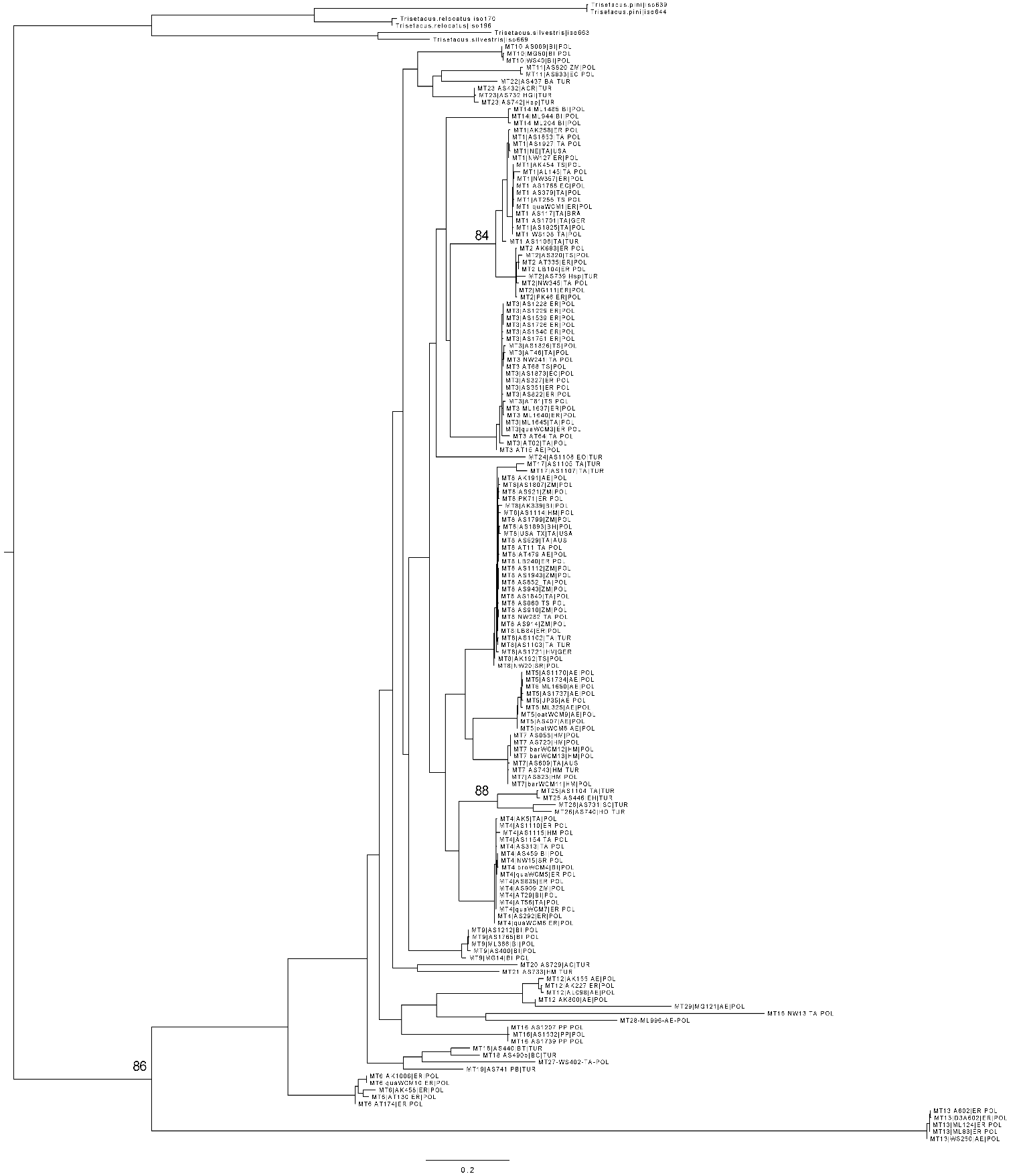
**

**Figure S1** Maximum Likelihood (ML) tree constructed using GTR+G model for the mtDNA Cox1 sequences of the wheat curl mite (WCM) species complex and outgroup species.

**
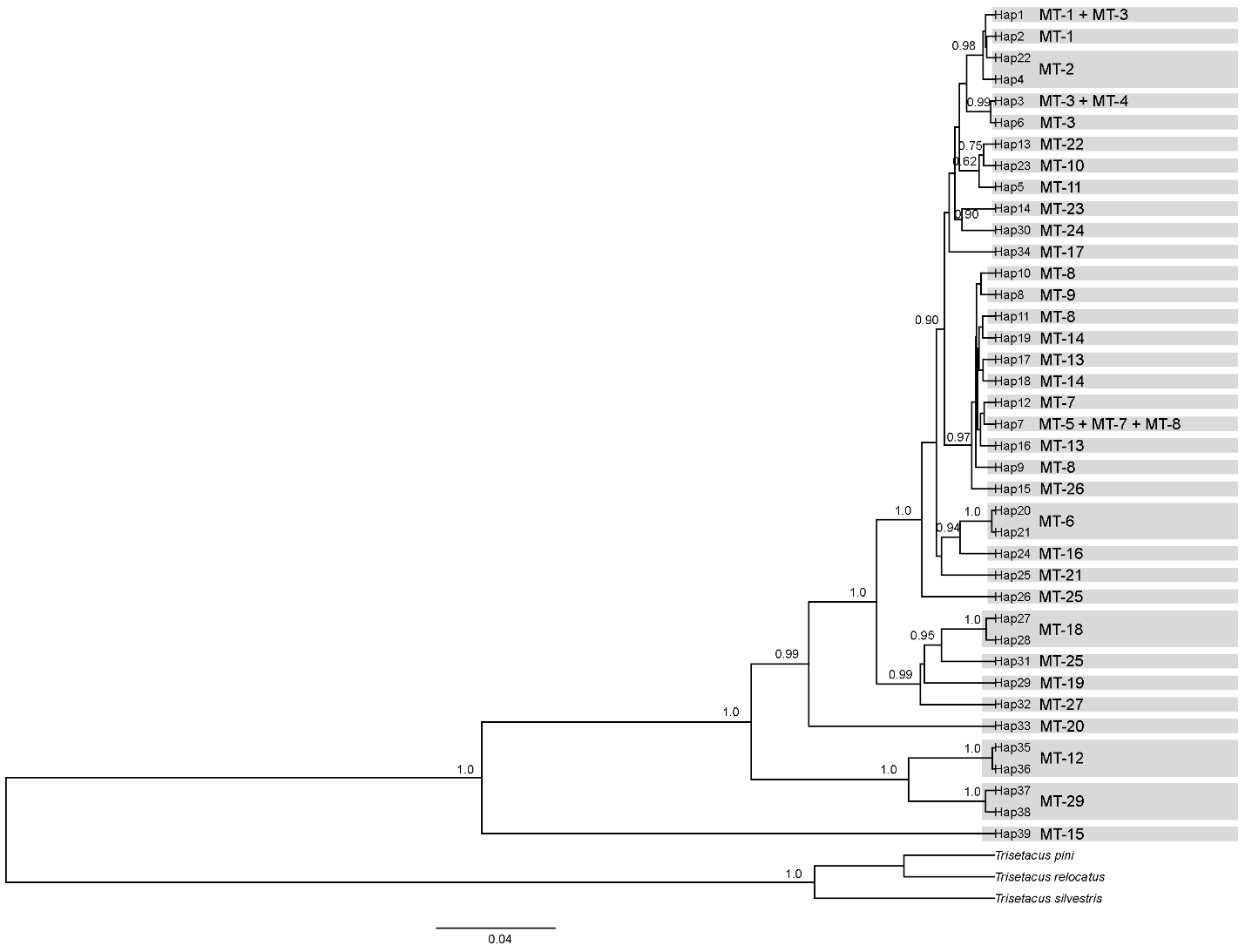
**

**Figure S2** Bayesian inference (BI) tree constructed using the GTR+G model for the haplotypes of 28S rDNA D2 region of the wheat curl mite (WCM) species complex and outgroup species. Numbers above branches are Bayesian posterior probabilities; only values > 0.6 are shown.

**
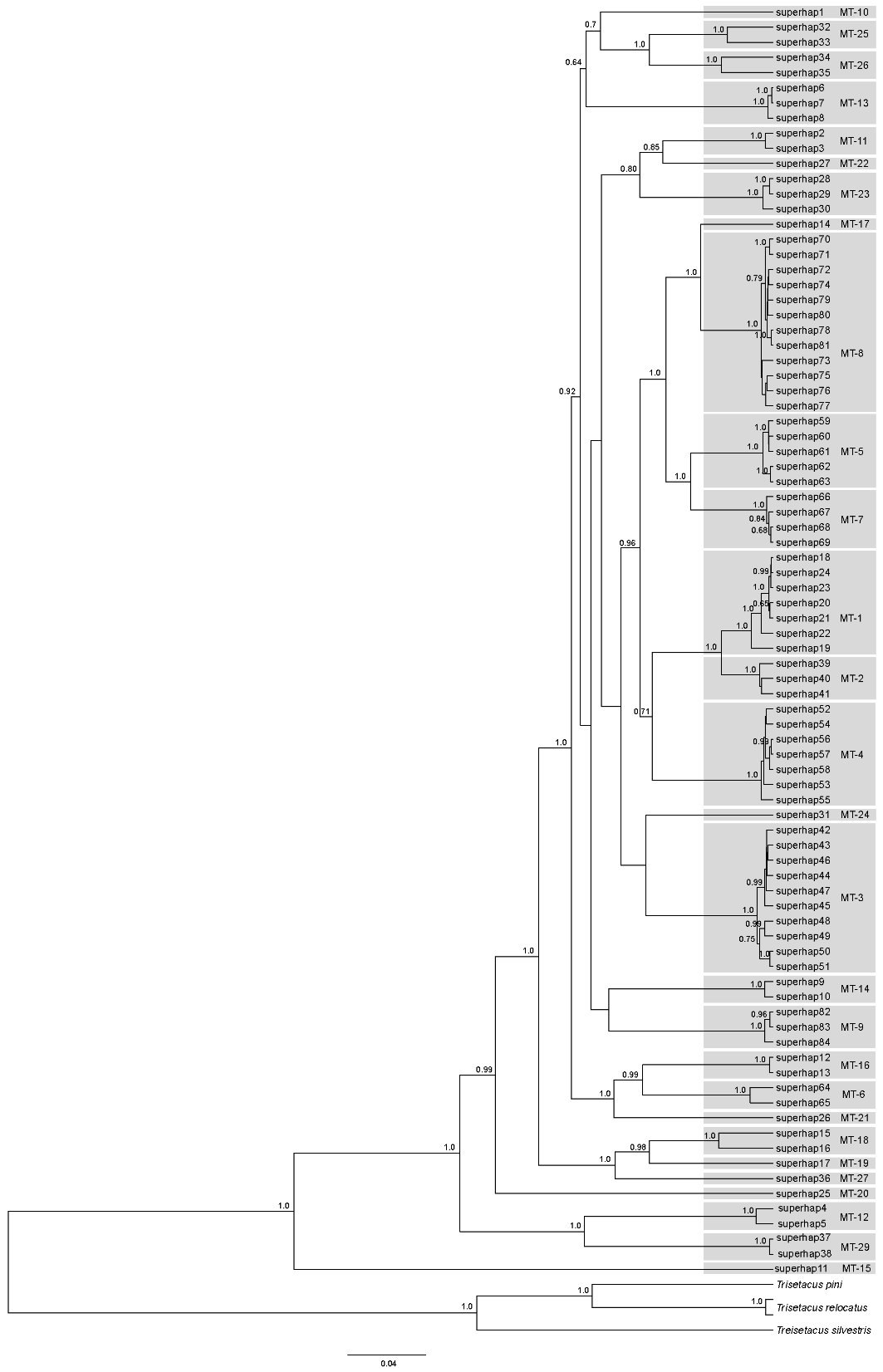
**

**Figure S3** Bayesian inference (BI) tree constructed using the GTR+G model for the combined mtDNA Cox1 and 28S rDNA D2 gene fragments of the wheat curl mite (WCM) species complex and outgroup species. Numbers above branches are Bayesian posterior probabilities; only values > 0.6 are shown.
