# Supplementary material for: Genetics of lineage diversification and the evolution of host usage in the economically important wheat curl mite, Aceria tosichella Keifer, 1969
Source: BMC Evol Biol. 2018 Aug 7;18:122. doi: 10.1186/s12862-018-1234-x (PMC6081818; doi:10.1186/s12862-018-1234-x)
Supplement: Supplementary file 4 — Mismatch distributions of WCM genetic lineages. (DOCX 747 kb) [file 12862_2018_1234_MOESM4_ESM.docx]

**Additional file 4:** Mismatch distributions of WCM genetic lineages


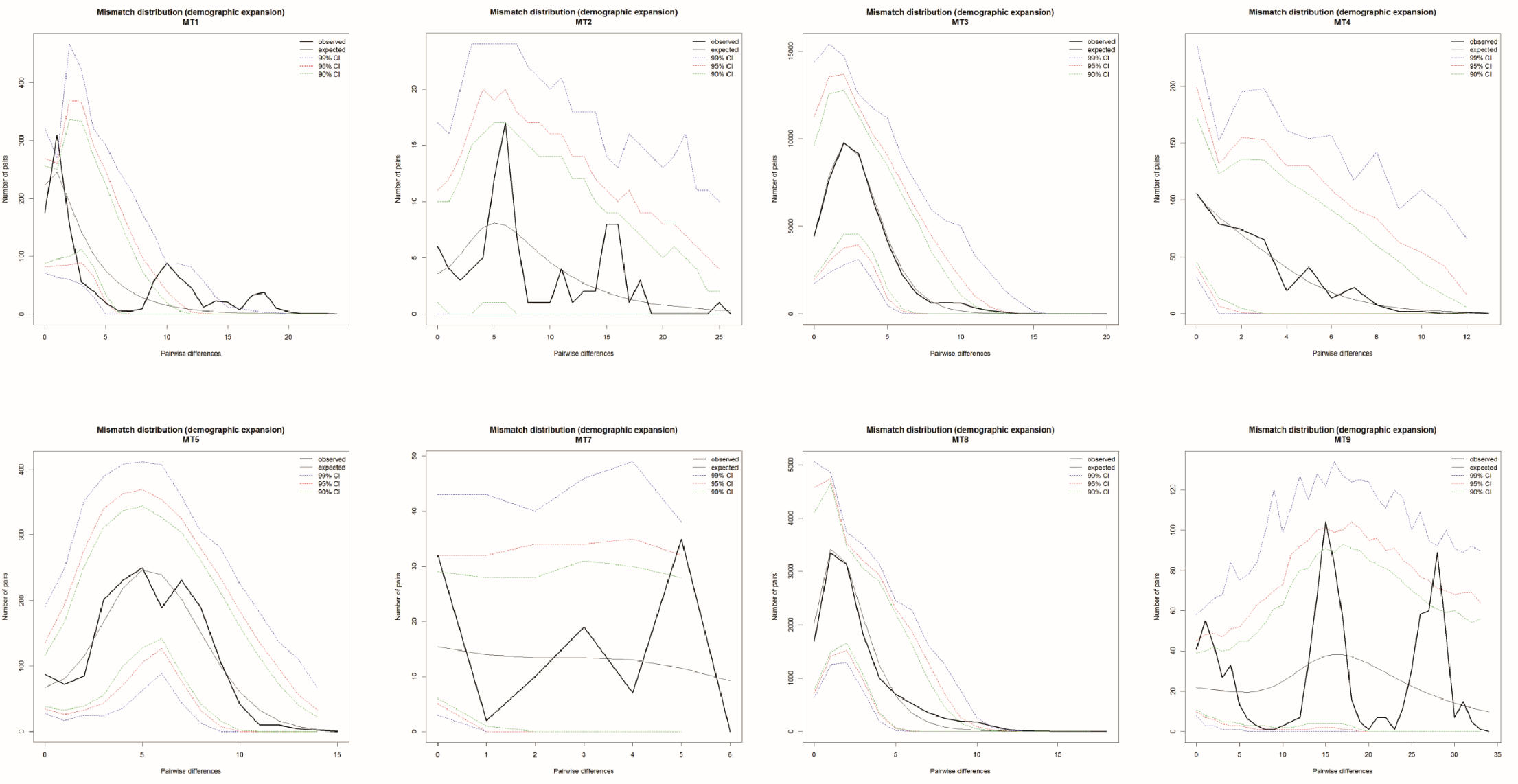


**Figure S4** Mismatch distributions of WCM genetic lineages under the demographic expansion model inferred from the mtDNA Cox1.
