# Supplementary material for: Genetics of lineage diversification and the evolution of host usage in the economically important wheat curl mite, Aceria tosichella Keifer, 1969
Source: BMC Evol Biol. 2018 Aug 7;18:122. doi: 10.1186/s12862-018-1234-x (PMC6081818; doi:10.1186/s12862-018-1234-x)
Supplement: Supplementary file 5 — Haplotype networks for the mtDNA Cox1 wheat curl mite (WCM). (DOCX 950 kb) [file 12862_2018_1234_MOESM5_ESM.docx]

**Additional file 5:** Haplotype networks for the mtDNA Cox1 wheat curl mite (WCM)

**
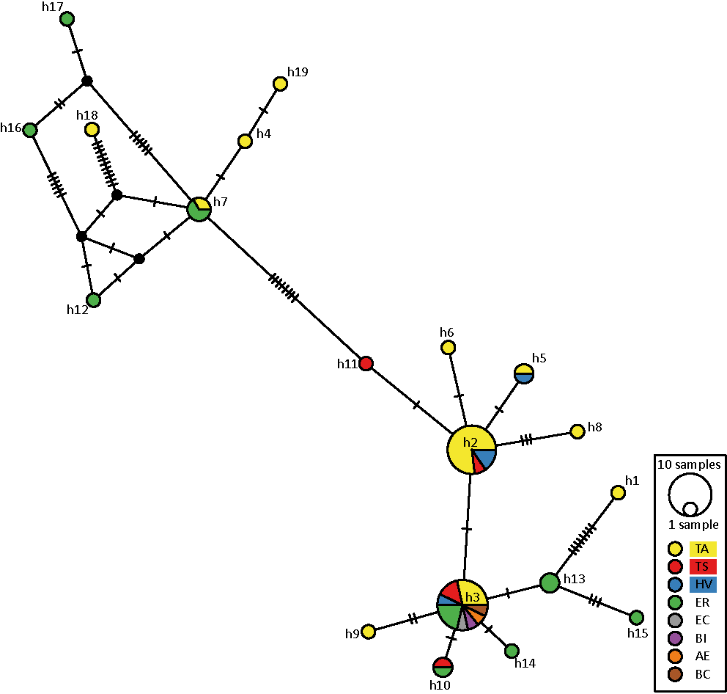
**

**Figure S5** Median-joining haplotype networks for the mtDNA Cox1 wheat curl mite (WCM) lineage MT-1. The size of each circle is proportional to the haplotypes frequency, with the numbers indicating particular haplotypes. Each vertical line indicates one mutational step; small black circles represent intermediate haplotypes not observed. The edge colors of the circles indicate the host plants from which WCM specimens were collected, which are abbreviated in the legend (TA: wheat, TS: triticale, HV: barley, ER: quackgrass, EC: cockspur, BI: smooth brome, AE: tall oat-grass, BC: *Bromus cappadocicus*). Color boxes in the legend indicate cereal hosts.

**
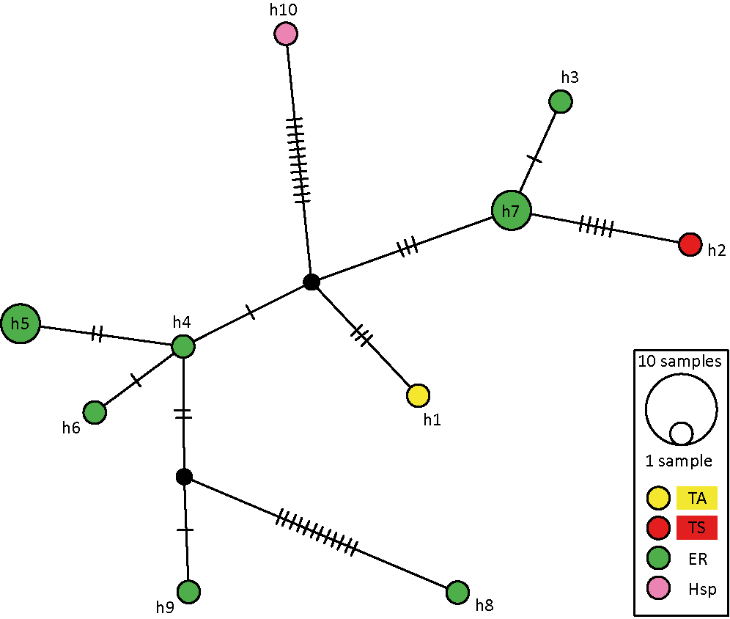
**

**Figure S6** Median-joining haplotype networks for the mtDNA Cox1 wheat curl mite (WCM) lineage MT-2. The size of each circle is proportional to the haplotypes frequency, with the numbers indicating particular haplotypes. Each vertical line indicates one mutational step; small black circles represent intermediate haplotypes not observed. The edge colors of the circles indicate the host plants from which WCM specimens were collected, which are abbreviated in the legend (TA: wheat, TS: triticale, ER: quackgrass, Hsp: *Hordeum* sp.). Color boxes in the legend indicate cereal hosts.

**
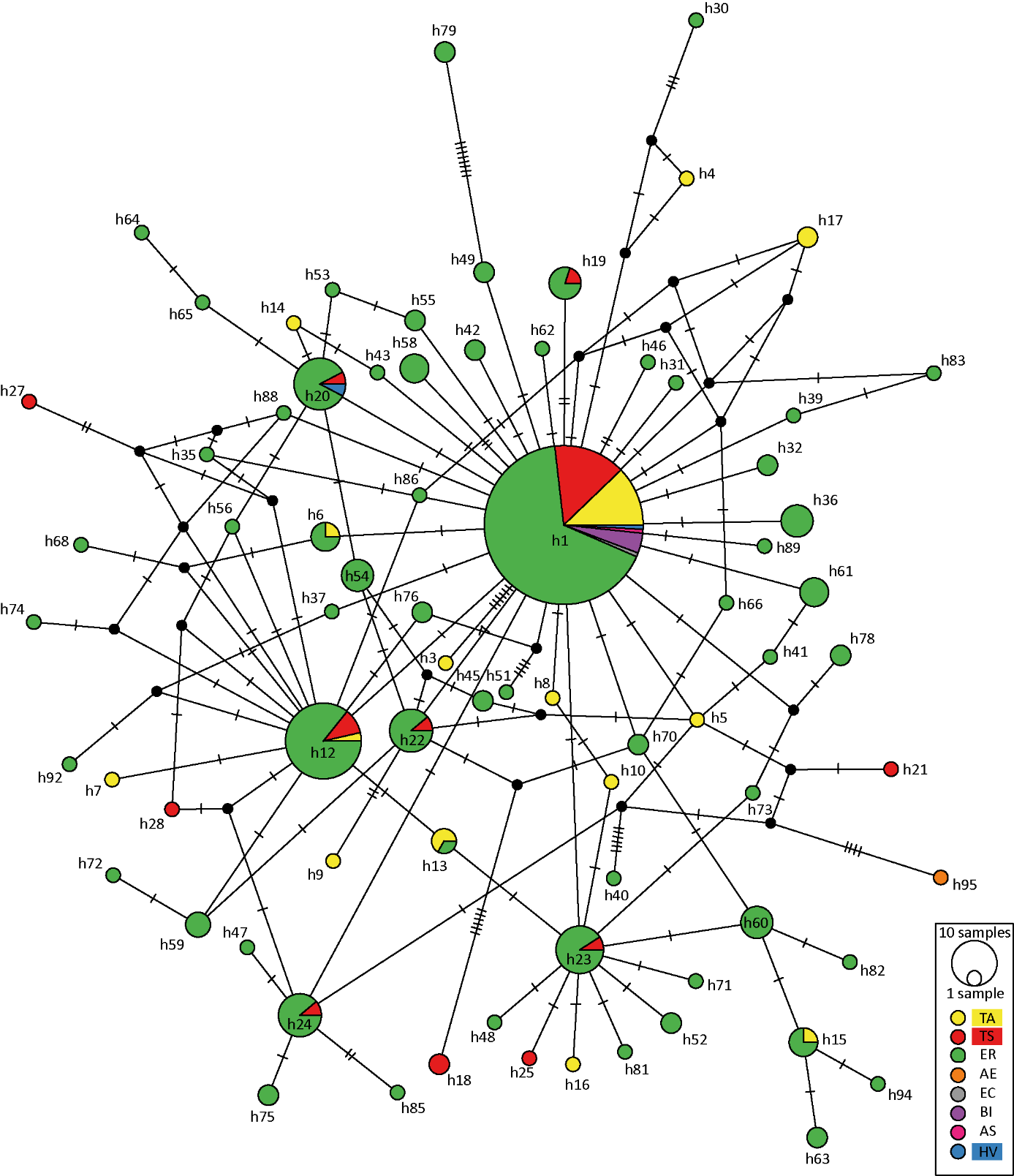
**

**Figure S7** Median-joining haplotype networks for the mtDNA Cox1 wheat curl mite (WCM) lineage MT-3. The size of each circle is proportional to the haplotypes frequency, with the numbers indicating particular haplotypes. Each vertical line indicates one mutational step; small black circles represent intermediate haplotypes not observed. The edge colors of the circles indicate the host plants from which WCM specimens were collected, which are abbreviated in the legend (TA: wheat, TS: triticale, ER: quackgrass, AE: tall oat-grass, EC: cockspur, BI: smooth brome, AS: oat, HV: barley). Color boxes in the legend indicate cereal hosts.

**
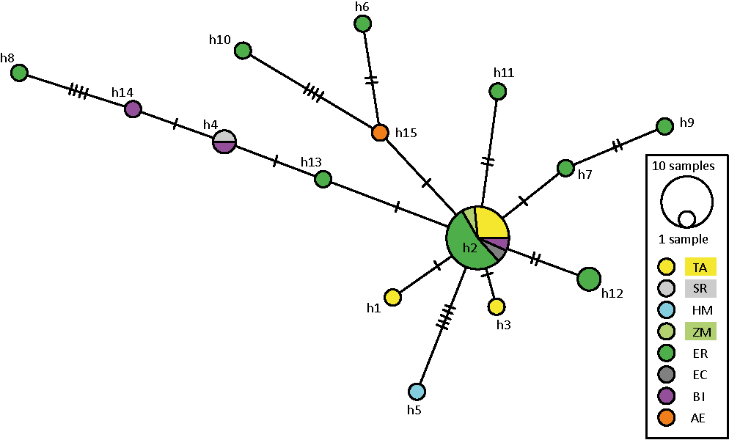
**

**Figure S8** Median-joining haplotype networks for the mtDNA Cox1 wheat curl mite (WCM) lineage MT-4. The size of each circle is proportional to the haplotypes frequency, with the numbers indicating particular haplotypes. Each vertical line indicates one mutational step; small black circles represent intermediate haplotypes not observed. The edge colors of the circles indicate the host plants from which WCM specimens were collected, which are abbreviated in the legend (TA: wheat, SR: rye, HM: wall barley, ZM: maize, ER: quackgrass, EC: cockspur, BI: smooth brome, AE: tall oat-grass). Color boxes in the legend indicate cereal hosts.

**
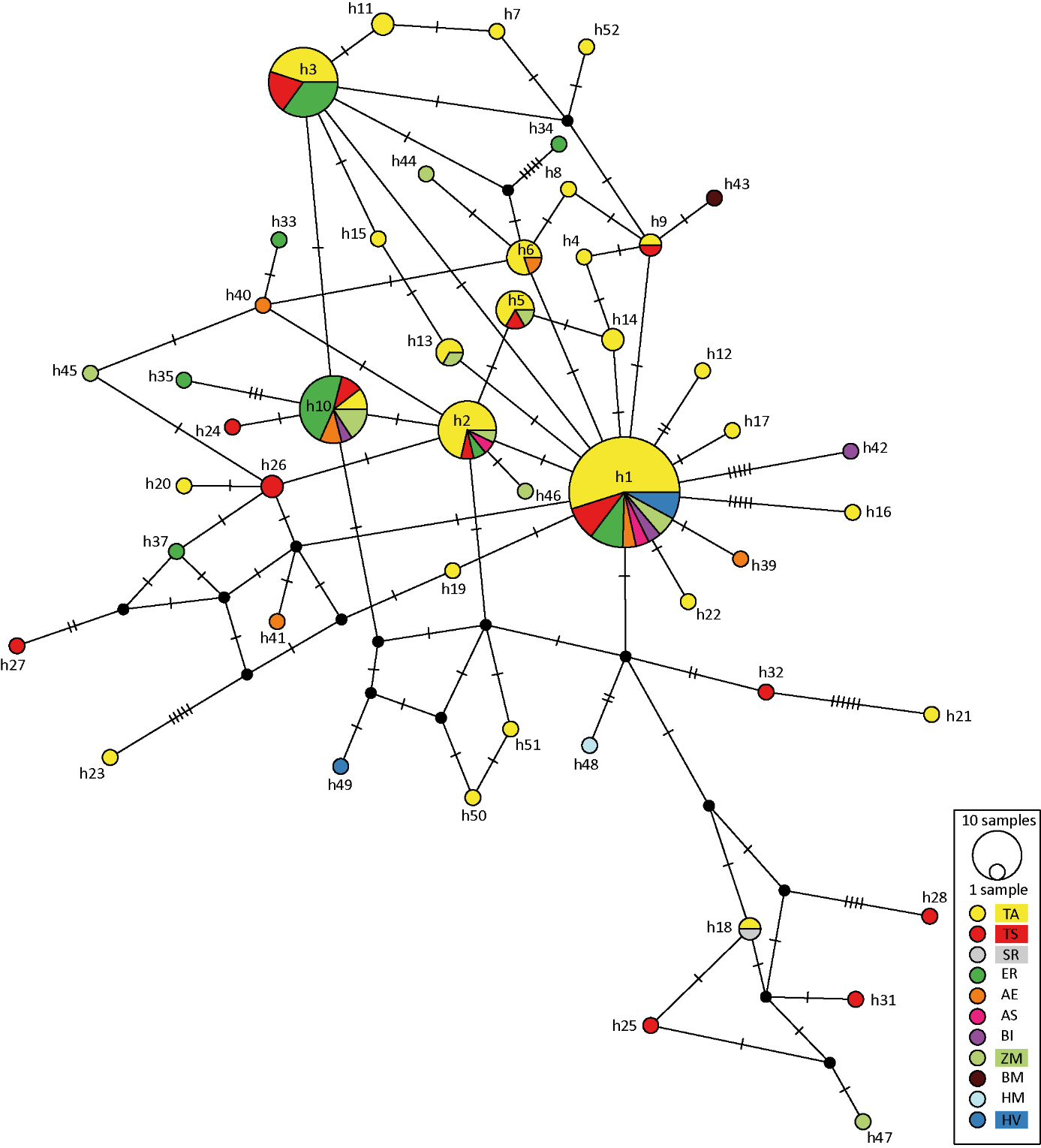
**

**Figure S9** Median-joining haplotype networks for the mtDNA Cox1 wheat curl mite (WCM) lineage MT-8. The size of each circle is proportional to the haplotypes frequency, with the numbers indicating particular haplotypes. Each vertical line indicates one mutational step; small black circles represent intermediate haplotypes not observed. The edge colors of the circles indicate the host plants from which WCM specimens were collected, which are abbreviated in the legend (TA: wheat, TS: triticale, SR: rye, ER: quackgrass, AE: tall oat-grass, AS: oat, BI: smooth brome, ZM: maize, BH: soft brome, HM: wall barley, HV: barley). Color boxes in the legend indicate cereal hosts.
